# Sequential Optimization Approach Toward an Azapeptide‐Based SARS‐CoV‐2 Main Protease Inhibitor

**DOI:** 10.1002/ardp.70175

**Published:** 2025-12-23

**Authors:** Rabea Voget, Victoria Steiger, Julian Breidenbach, Katharina Sylvester, Christin Müller‐Ruttloff, Chun‐Chiao Yang, John Ziebuhr, Norbert Sträter, Christa E. Müller, Michael Gütschow

**Affiliations:** ^1^ Pharmaceutical Institute, Pharmaceutical & Medicinal Chemistry University of Bonn Bonn Germany; ^2^ Institute of Medical Virology Justus Liebig University Giessen Giessen Germany; ^3^ Institute of Bioanalytical Chemistry, Center for Biotechnology and Biomedicine Leipzig University Leipzig Germany

**Keywords:** azapeptides, chloroacetohydrazides, peptidomimetics, protease inhibitors, SARS‐CoV‐2 main protease

## Abstract

The severe acute respiratory syndrome coronavirus 2 (SARS‐CoV‐2), causative agent of the coronavirus disease 2019 (COVID‐19), is still circulating and posing a health threat to the global population. Its main protease (M^pro^) constitutes an excellent target for the development of antivirals due to its indispensable role in the viral replication cycle. In this work, we employed a sequential approach to identify a potent azapeptide‐based M^pro^ inhibitor. Starting from a series of small‐molecule peptidomimetics, identical in their scaffold but equipped with different cysteine‐reactive groups, we identified auspicious warheads. The combination of selected moieties with an optimized, previously described P1–P4 azapeptide structure resulted in a potent M^pro^ inactivator (**12**) with a *k*_inac_/*K*_i_ value of 78,900 M^–1^s^–1^. The chloracetohydrazide derivative **12** exhibited antiviral activity (EC_50_ = 0.47 µM), no cytotoxicity, and plasma stability. The molecular interaction of **12** with M^pro^ was elucidated by an X‐ray crystal structure. A thioether linkage was generated through a nucleophilic substitution of chloride by the active‐site thiolate, giving rise to irreversible inhibition.

## Graphical abstract

A series of small‐molecule azapeptides with various cysteine‐reactive electrophilic groups was designed, synthesized, and investigated for M^pro^ inhibition. Particularly suitable warheads were identified, and their linkage to a structurally expanded peptidomimetic scaffold afforded the potent lead compound **12**, with *k*_inac_/*K*_i_ = 78,900 M^–1^s^–1^ and an antiviral EC_50_ of 0.47 µM. The covalent M^pro^‐inhibitor complex was elucidated by X‐ray crystallography.

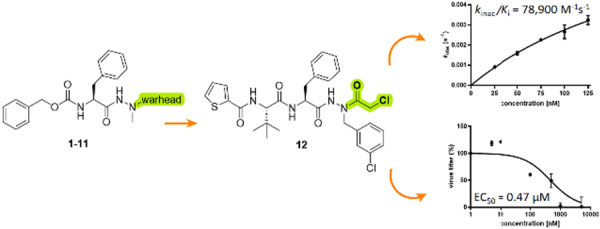

## 1 Introduction

**Figure 1:**
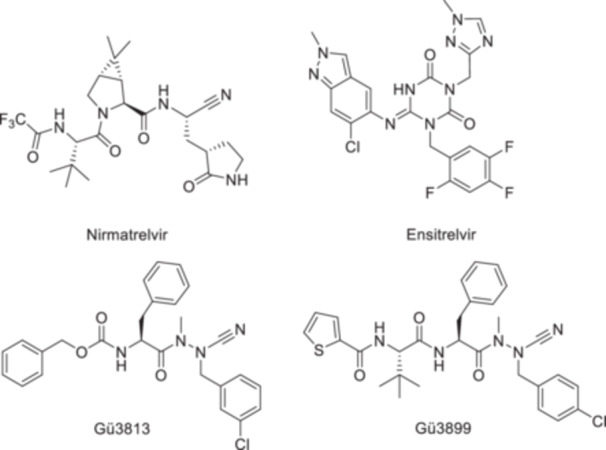
Top. Structures of the approved SARS‐CoV‐2 M^pro^ inhibitors nirmatrelvir, a covalent, reversible inhibitor, and ensitrelvir, a non‐covalent inhibitor. Bottom. Structures of the M^pro^ inhibiting azadipeptide nitrile Gü3813 and the azatripeptide nitrile Gü3899.

The severe acute respiratory syndrome coronavirus 2 (SARS‐CoV‐2) caused the pandemic of coronavirus disease 2019 (COVID‐19), whereupon research in the field of antiviral drug discovery intensified considerably. Global efforts led to the approval of effective vaccines and small‐molecule inhibitors of the main protease (M^pro^) of SARS‐CoV‐2. However, viral evolution resulting in the emergence of variants and the elevated risk of drug resistance requires the continuous discovery of new antiviral drugs [1, 2, 3]. M^pro^ (also designated as 3CL^pro^) is an attractive target due to its crucial role in the viral replication process, its comprehensive biochemical characterization, and the lack of a human analog [4, 5]. Significant efforts have been dedicated to developing M^pro^ inhibitors as an antiviral medication, and a small number of drugs have been approved, including nirmatrelvir and ensitrelvir (Figure 1), along with the clinical evaluation of numerous inhibitor candidates [1, 6, 7].

The cysteine protease M^pro^ is autocatalytically released from the two polyproteins pp1a and pp1ab encoded by open reading frames 1a and 1b of the SARS‐CoV‐2 genome. To produce the multi‐subunit complexes required for viral replication and transcription in infected host cells, the polyproteins are further processed by M^pro^ and a papain‐like protease (PL^pro^) into 16 non‐structural proteins (nsps) [4, 5, 7, 8]. M^pro^ contains three domains, of which domains I and II form the active site pocket, while domain III facilitates homodimerization, which is crucial for proteolytic activity [4]. Substrate cleavage is catalyzed in the course of an acyl transfer, initiated by the nucleophilic attack of the catalytic Cys145 on the carbonyl carbon of the scissile peptide bond, forming a covalent *S*‐acyl enzyme intermediate, followed by its rapid hydrolysis. This mechanism serves as the basis for developing covalently acting peptidomimetic protease inhibitors with electrophilic warheads [9, 10]. The design of such M^pro^ inhibitors followed the established concepts to generate peptidomimetics and is typically guided by the M^pro^ substrate specificity, with glutamine as the preferred amino acid in the P1 position, representing a completely conserved feature in the M^pro^ specificity profile [5, 11]. A wide range of inhibitors bear (*S*)‐3‐methylpyrrolidine‐2‐on in the side chain of the P1 amino acid, featuring the most frequently used glutamine mimetic in peptidic M^pro^ inhibitors [4, 10, 12]. This strategy enabled the development of nirmatrelvir (Figure 1) [6], a key advancement in covalent drug research. Its peptidic composition, incorporating three non‐natural amino acids, reflects a pronounced promiscuity for interaction with high‐affinity drugs along the S4–S1 binding region of M^pro^, caused by a significant structural plasticity of the enzyme [13]. A variety of electrophilic warheads were introduced at the C‐terminus of peptidomimetic M^pro^ inhibitors [14], including nitrile [6, 8], α‐keto amide [4, 15], aldehyde [16, 17, 18], acyloxymethyl ketone [19], aryl ketone [20], or vinyl methyl ketone moieties [21, 22].

We have previously introduced the chemotype of azapeptide nitriles as covalent inhibitors of cysteine proteases [23, 24]. Tailored representatives were identified as potent inhibitors of M^pro^ [25]. In a recent study, 73 new azapeptide nitriles were conceptualized, synthesized, and kinetically evaluated, which allowed for an active‐site scanning of the S1, S2, and S3/S4 pockets of M^pro^ [26]. Compound Gü3813 (Figure 1), for example, was identified as a particularly potent inhibitor out of a subseries of compounds with a diversified P1 position. Gü3899 (Figure 1), which shares the P3 amino acid *tert*‐leucine with nirmatrelvir, was obtained in the course of a systematic variation at the P3/P4 position. For macrocyclic inhibitors, a *meta*‐chloro substitution at the aromatic core of the P1 side chain was employed [26]. Overall, aromatic residues were preferred at P1 and P2 positions, a feature that differed from that of the carba analogs [27]. To prevent unwanted 5‐exo‐dig cyclizations [23, 28, 29], azapeptide nitriles necessarily bear alkyl substituents at both nitrogens of the C‐terminal aza‐amino nitrile building block.

Beyond azapeptide nitriles, the assembly of M^pro^ inhibitors with a C‐terminal aza‐amino acid has been realized, and potent activity‐based probes and protease inactivators have been obtained [30, 31, 32]. The present study is aimed at the development of novel azapeptides and their biochemical characterization as M^pro^ inhibitors. For this purpose, we used appropriate scaffolds from our previous work on azapeptide nitriles and performed warhead “hopping” experiments to generate new chemical entities with M^pro^ inhibitory activity.

## 2 Results and Discussion

### 2.1 Synthesis of Small‐Molecule Inhibitors With Miscellaneous Warheads

**Scheme 1:**
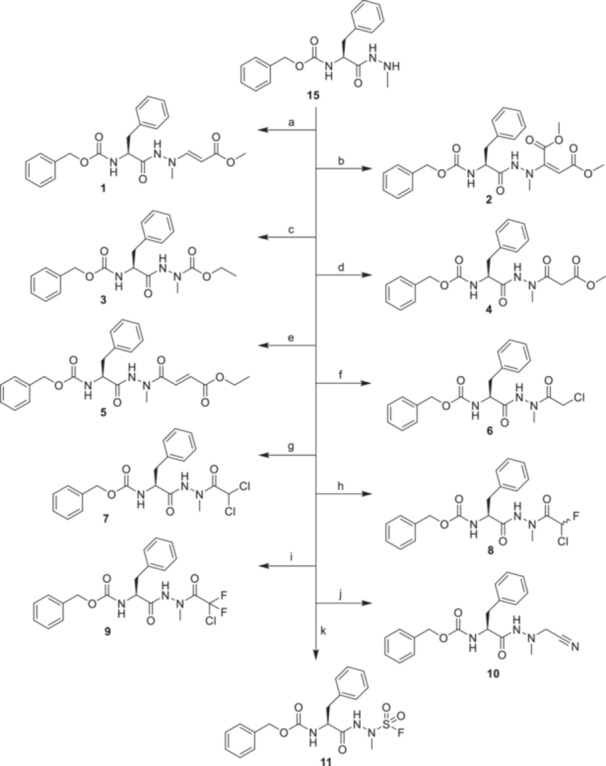
Synthesis of small‐molecule M^pro^ inhibitors with different warheads **1–11.** Reagents and conditions: (a) methyl propiolate, AcOH, 1,4‐dioxane, 60°C, 16 h; (b) dimethyl but‐2‐ynedioate, AcOH, 1,4‐dioxane, 60°C, 20 h; (c) ethyl chloroformate, *N*‐methylmorpholine, THF, 0°C to rt, 2 h; (d) methyl 3‐bromopropiolate, AcOH, 1,4‐dioxane, 60°C, 16 h *or* methyl malonyl chloride, DIPEA, CH_2_Cl_2_, rt, 20 h; (e) monoethyl fumarate, *N*‐methylmorpholine, ethyl chloroformate, DIPEA, THF, 0°C to rt, 20 h; (f) chloroacetyl chloride, DIPEA, CH_2_Cl_2_, rt, 2 h; (g) dichloroacetic anhydride, DIPEA, CH_2_Cl_2_, rt, 18 h; (h) **16**, HATU, DIPEA, DMF, 0°C to rt, 18 h; (i) sodium 2‐chloro‐2,2‐difluoroacetate, HATU, DIPEA, DMF, 0°C to rt, 18 h; (j) Cs_2_CO_3_, NaI, 2‐chloroacetonitrile, acetone, 75°C, 5 h; (k) 1‐(fluorosulfonyl)‐2,3‐dimethyl‐1*H*‐imidazol‐3‐ium trifluoromethanesulfonate, CH_2_Cl_2_, 0°C to rt, 4.5 h.

An initial series of small peptidomimetic inhibitors bearing various electrophilic warheads was devised. The compounds consist of an N‐terminal benzyloxycarbonyl‐protected l‐phenylalanine in P2, consistent with the N‐terminal substructure of Gü3813 (Figure 1), and differently modified aza‐alanine building blocks in the P1 position. In contrast to the aforementioned azanitrile structures, the methyl group linked to the inner nitrogen atom of the hydrazide moiety was removed, as the hydrogen in this position was assumed to enhance the inhibitory efficacy owing to additional interactions with the substrate binding site of the protease [11]. The final products of this warhead hopping are depicted in Scheme 1. The inhibitor candidates **1–11** were derived from a common precursor, hydrazide **15**. It was accessible from Z‐Phe‐OH and methylhydrazine via a mixed anhydride intermediate, and the resulting two regioisomers were separated using silica gel column chromatography [23].

Vinylation of the terminal amino group of **15** was accomplished through nucleophilic addition to the C–C triple bond of either methyl propiolate or dimethyl but‐2‐ynedionate, catalyzed by glacial acetic acid [33]. The pattern of the α‐hydrazino‐substituted Michael acceptor system present in **1** and **2** has not been described yet. To provide the ethyl carbazate **3**, ethyl chloroformate was applied.

The reaction between precursor **15** and methyl 3‐bromopropiolate, catalyzed by glacial acetic acid, was performed with the intention of synthesizing either the bromo‐substituted vinylated derivative or the corresponding dehydrohalogenation product with the re‐formed C‐C‐triple bond. However, analytical data suggested that compound **4** was obtained. The reaction of alkyl 3‐bromopropiolates with N‐nucleophiles has barely been studied; ynamines were produced with a small selection of secondary amines [34], alkynes from tertiary carbanions [35], and ketene aminals or ketene acetals in the course of an addition‐elimination reaction sequence [36, 37]. In our case, exposure to water, acting as the second nucleophile during extraction, and subsequent keto‐enol tautomerism afforded compound **4**. To confirm the structure of **4**, compound **15** was treated with methyl malonyl chloride to independently obtain the identical reference product.

Compound **5** was synthesized by activating monoethyl fumarate to a mixed anhydride, followed by the addition of the nucleophile **15**. Compounds **6–9** constitute halogenated acetohydrazide derivatives. Chloroacetyl and dichloroacetyl derivatives **6** and **7** were produced with the corresponding carboxylic acid chloride and anhydride, respectively. For the synthesis of **8**, ethyl 2‐chloro‐2‐fluoroacetate was saponified under mild conditions [38], and the resulting sodium salt **16** was subjected to a uronium salt‐mediated coupling reaction with **15**. Similarly, **9** was obtained from sodium 2‐chloro‐2,2‐difluoroactate. We applied 2‐choroacetonitrile for the alkylation of the terminal nitrogen of **15**, which gave the product **10**. The last compound in this series, **11**, which bears a fluorosulfonyl moiety, was obtained by utilizing 1‐(fluorosulfonyl)‐2,3‐dimethyl‐1*H*‐imidazol‐3‐ium trifluoromethanesulfonate, a shelf‐stable, crystalline solid analog of SO_2_F_2_ [39]. The synthesis of **11** was accomplished by stirring the imidazolium salt and **15** in anhydrous methylene chloride without additional reagents. A fluorosulfonyl warhead had not been installed on azapeptides so far.

### 2.2 Biochemical Evaluation of Small‐Molecule Inhibitors With Miscellaneous Warheads

Our target, SARS‐CoV‐2 M^pro^, was expressed and purified as described [26, 40, 41]. We used a fluorogenic substrate developed in our group, Boc‐Abu‐Tle‐Leu‐Gln‐AMC, to monitor the proteolytic activity of M^pro^ [25]. To examine the inhibitory potential that arises from the different electrophilic entities, the small‐molecule inhibitors **1**–**11** were initially screened at 10 and 50 µM, and the inhibition rates were determined (Table 1). Compounds with inhibition rates below 50% at 50 µM were not further biochemically characterized. For inhibitors with percentage rates of ≥ 50% (**6**, **7**, and **9**), the IC_50_ value, the inhibition constant *K*_i_, and (in the case of irreversible inhibition) the second‐order rate constant of enzyme inactivation *k*_inac_/*K*_i_ were determined (Table 2).

**Table 1:** SARS‐CoV‐2 M^pro^ inhibition rates of small‐molecule inhibitors **1–11** at 10 µM and 50 µM.

Compound	Inhibition @ [I] = 10 µM (%)^a^	Inhibition @ [I] = 50 µM (%)^a^
**1**	4	0
**2**	1	2
**3**	2	3
**4**	0	0
**5**	7	13
**6**	19	55^b^
**7**	85	100^b^
**8**	10	16
**9**	9	15
**10**	10	11
**11**	40	80^b^

^a^ Product formation was determined after 10 min.

^b^ For kinetic characterization of inhibitors **6**, **7**, and **11**, see Table 2.

**Table 2:** Kinetic parameters of SARS‐CoV‐2 M^pro^ inhibitors **6**, **7**, and **11**.

Compound	IC_50_ (µM)^a^	*K*_i_ (µM)	*k*_inac_/*K*_i_ (M^−1^s^−1^)
**6**	30.1 ± 1.7	14.8 ± 0.8	133 ± 8
**7**	2.47 ± 0.18	1.22 ± 0.09	n.d.^b^
**11**	14.7 ± 0.7	7.24 ± 0.34	54.6 ± 25.0

^a^ Product formation was determined after 10 min.

^b^ Not determined. Compound **7** showed reversible inhibition.

The warhead hopping was performed to identify a new warhead structure. However, azapeptides **1** and **2**, both with Michael acceptor moieties, were inactive against M^pro^, just as the malonomonohydrazide derivative **4**. The hydrazinoacetonitrile derivative **10** did not exhibit notable inhibitory properties either. The installation of a carbazate (in **3**) and a fumarate moiety (in **5**) did not cause protease inhibition; both warhead types have already been established in other azapeptides [30, 32], and the latter had led to a potent M^pro^ inhibitor [30].

Out of the four halogenated acetohydrazide derivatives (**6**–**9**), two representatives (**6** and **7**) were identified as M^pro^ inhibitors. This finding enabled us to narrow down the number of test candidates in the next step of synthesis. The chlorofluoroacetohydrazide and trihaloacetohydrazide derivatives **8** and **9** failed to strongly inhibit M^pro^. The reactive moiety of **8** has been employed in modifiers of kinase cysteine residues [38]. When introduced into tailored azadipeptides, potent M^pro^ inhibitors have been developed, with IC_50_ values of 3.8 nM for the *R*‐configured and 28 nM for the *S*‐configured stereoisomer [31]. Hence, although we investigated a mixture of two diastereomers, the weak inhibitory activity of **8** was unexpected. This result clearly reflects that the peptidic part of the inhibitor affects the efficacy of the warhead, because the protease can adjust its binding site to effectively recognize a specific inhibitor molecule.

**Figure 2:**
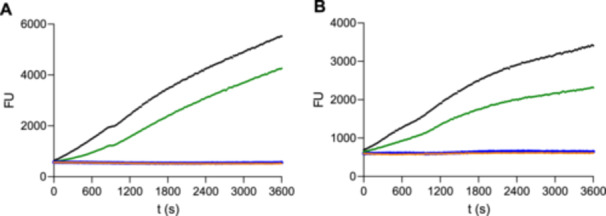
Determination of M^pro^ inhibition reversibility for compounds **6**, **7**, and **11** (A) and for **12–14** (B). Protease and inhibitor were incubated for 25 min and subsequently diluted 100‐fold by the addition of the M^pro^ substrate‐containing assay buffer. While reversible inhibitors dissociate from the active site upon jump dilution, allowing for observable product formation, no recovery of enzyme activity occurs under irreversible inhibition. (A) Inhibitor **7** (green) proves to be reversible, while **6** (orange) and **11** (blue) are irreversible. (B) Reversible inhibition is apparent for **13** (green), irreversible inhibition for **12** (orange), and **14** (blue). The control reaction in the absence of inhibitors is shown in black.

The kinetic parameters of inhibitors **6**, **7**, and **11** are shown in Table 2. Gratifyingly, we identified a new warhead structure, present in the fluorosulfonyl derivative **11**. Our data confirmed the suitability of the chloroacetohydrazide (in **6**) and the dichloroacetohydrazide moiety (in **7**) for the design of M^pro^‐inhibiting azapeptides [30, 32]. These cysteine‐reactive groups have also been installed in non‐peptidic covalent M^pro^ inhibitors [42]. The lowest IC_50_ (2.47 µM) and *K*_i_ (1.22 µM) values were obtained for compound **7**. A previously studied azanitrile inhibitor with the same core structure, except for a methyl group on the inner nitrogen atom of the hydrazide moiety, also showed a single‐digit micromolar IC_50_ value (2.19 µM), but, in comparison to **6** and **11**, a higher second‐order rate constant of inactivation (*k*_inac_/*K*_i_ = 1,260 M^–1^s^–1^) [25]. In contrast to **6**, **11**, and the reference azanitrile, which were found to be irreversible inhibitors with no enzyme activation after jump dilution (Figure 2), the dichloroacetohydrazide derivative **7** blocked the active site of M^pro^ in a reversible manner, allowing for enzyme reactivation. Therefore, a second‐order rate constant of enzyme inactivation, *k*_inac_/*K*_i_, could not be determined for **7**. Reversibility of M^pro^ inhibition may be advantageous when considering, for example, nirmatrelvir, a reversibly acting agent, which demonstrated high efficacy and a favorable safety profile [6]. Reversible inhibition is, moreover, expected to reduce off‐target reactivity and toxic effects [8]. Compound **11**, equipped with the fluorosulfonyl moiety, was observed to be not sufficiently stable in aqueous media, and its warhead was therefore not chosen for the subsequent syntheses. Hence, for the second iterative step, we selected the warhead structures of **6**, **7**, and **8** to be incorporated into structurally extended azapeptides.

### 2.3 Synthesis of Inhibitors With an Optimized P1–P4 Sequence

**Scheme 2:**
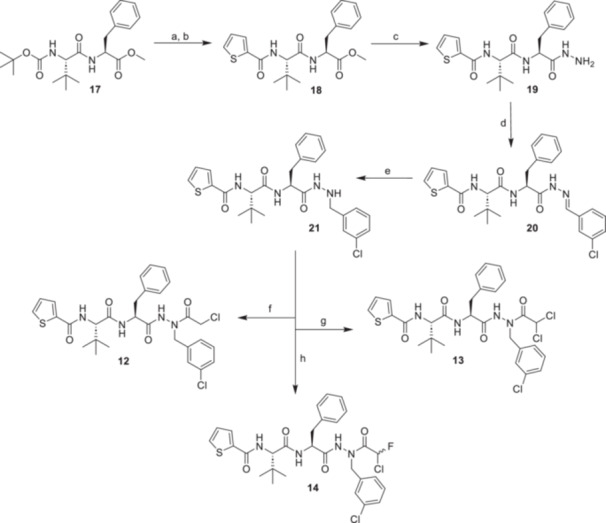
Synthesis of inhibitors **12–14** with an optimized P1–P4 sequence. Reagents and conditions: (a) CH_2_Cl_2_, TFA, rt, 2 h; (b) thiophene‐2‐carbonyl chloride, NEt_3_, DMAP, CH_2_Cl_2_, 0°C to rt, 18 h; (c) N_2_H_4 _× H_2_O, EtOH, 95°C, 4.5 h, then rt, 16 h; (d) 3‐chlorobenzaldehyde, Na_2_SO_4_, EtOH, rt, 16 h; (e) DMAB, *p*‐TsOH × H_2_O, CH_2_Cl_2_, MeOH, 0°C to rt, 16 h; (f) chloroacetyl chloride, DIPEA, CH_2_Cl_2_, rt, 18 h; (g) dichloroacetic anhydride, DIPEA, CH_2_Cl_2_, rt, 18 h; (h) **16**, DIPEA, HATU, DMF, 0°C to rt, 18 h.

The inhibitors **12–14** were synthesized as shown in Scheme 2. Based on previous findings on M^pro^ inhibiting azapeptide nitriles, residues that displayed favorable interactions within the active site of M^pro^ were incorporated [25, 26]. We employed an optimized sequence accommodating the binding region from S1 to S4. The so‐designed scaffold included a 3‐chlorobenzyl side chain for the aza‐amino acid in P1 position, phenylalanine in P2, *tert*‐leucine in P3, and a 2‐thiophenecarbonyl capping group. The azapeptidomimetic structure was assembled starting from position P2, continuing to the N‐terminus before coupling the P1 residue and subsequently the warhead to the core structure **21**. In the first step, Boc‐protected *tert*‐leucine was coupled to a phenylalanine methyl ester with HATU to generate **17**, which was deprotected and subjected to a base‐catalyzed amide coupling with 2‐thiophenecarbonyl chloride to afford the dipeptide ester **18**. Subsequent hydrazinolysis gave **19**, which underwent a condensation reaction with 3‐chlorobenzaldehyde to produce the hydrazone **20**. As established previously [23], borane dimethylamine complex (DMAB) was applied for the reduction, providing the core structure **21**. The final inhibitors **12**–**14** were obtained by attaching the corresponding warheads as described above for compounds **6**–**8**.

### 2.4 Biological Evaluation of Inhibitors With an Optimized P1–P4 Sequence

As auspicious warheads, the chloroacetyl (**12**), dichloroacetyl (**13**), and chlorofluoroacetyl (**14**) groups were introduced to the structurally extended scaffold, and the resulting compounds were kinetically characterized with respect to their inhibitory efficacy against M^pro^ (Table 3). The most promising electrophilic entity of the initial warhead screening, the dichloroacetyl group, did not lead to a particularly potent inhibitor (**13**) with an inhibition rate below 50% at 50 µM and was therefore excluded from further kinetic measurements. Similar to its small‐molecule analog **7**, compound **13** showed a reversible behavior as an M^pro^ inhibitor (Figure 2).

**Table 3:** Inhibition rates and kinetic parameters of M^pro^ inhibitors **12–14**.

Compound	Inhibition @ [I] = 50 µM (%)^a^	IC_50_ (µM)^a^	*K*_i_ (µM)	*k*_inac_/*K*_i_ (M^**−**1^s^**−**1^)
**12**	100	0.0466 ± 0.0034	0.0230 ± 0.0017	78,900 ± 12,800
**13**	42	n.d.^b^	n.d.^b^	n.d.^b^
**14**	69	8.82 ± 1.22	4.34 ± 0.60	1,580 ± 210

^a^ Product formation was determined after 10 min.

^b^ Not determined. Compound **13** showed reversible inhibition.

**Figure 3:**
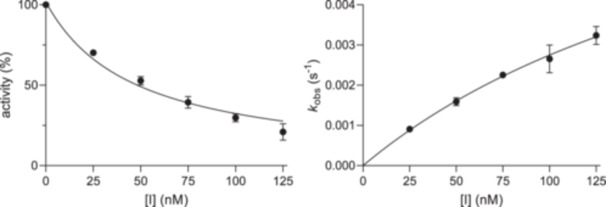
Kinetic characterization of irreversible M^pro^ inhibitor **12**. An IC_50_ value (left) of 46.6 nM and a *k*_inac_/*K*_i_ ratio (right) of 78,900 M^–1^s^–1^ were determined.

Tested via jump dilution, irreversible inhibition of M^pro^ was determined for both **12** and **14** (Figure 2). When coupled to the optimized peptidomimetic scaffold, the chlorofluoroacetohydrazide warhead (in **14**) demonstrated reactivity toward the catalytic cysteine of M^pro^. This is in accordance with literature data, although the IC_50_ value of 8.82 µM is three orders of magnitude higher than that of the reference chlorofluoroacetohydrazide inhibitor YH‐6 [31]. The chloroacetohydrazide‐containing inhibitor **12** conspicuously outperformed **14** and had a 50‐fold improved *k*_inac_/*K*_i_ value. For this most efficacious inhibitor **12**, an enormously increased second‐order rate constant was also detected in comparison to the small‐molecule counterpart **6** (78,900 M^–1^s^–^^1^ *vs.* 133 M^–1^s^–1^). Compound **12** exhibited a *K*_i_ value of 23 nM, close to those of nirmatrelvir and ensitrelvir (4 and 6 nM) [26, 43]. Our data confirm the observation that extended interactions between the binding pockets in the active‐site region of M^pro^ and the P1–P4 residues of the inhibitor can greatly contribute to inhibitory potency [1, 8, 25]. Compound **12** was identified as a potent SARS‐CoV‐2 M^pro^ inhibitor, indicated by the high *k*_inac_/*K*_i_ value reflecting fast enzyme inactivation, along with its low *K*_i_ value suggesting high affinity for M^pro^ (Figure 3).

### 2.5 X‐Ray Crystallography of SARS‐CoV‐2 **M^Pro^** in Complex With Inhibitor 12

**Figure 4:**
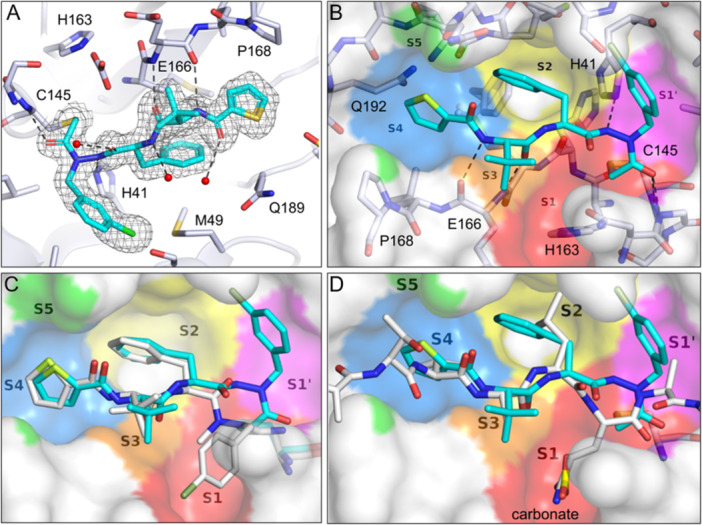
Crystal structure of inhibitor **12** bound to M^pro^ (PDB ID 9SDM, 1.79 Å resolution). (A) Electron density (2F_o_‐F_c_‐type, contoured at 1.0 σ_rms_) and binding mode of the covalently bound **12** (cyan) in chain A. Hydrogen bonding interactions to H41, E166, C145, and water molecules (red spheres) are indicated as dashed lines. (B) Molecular surface of M^pro^ with the binding sites of a peptide substrate labeled according to Schechter and Berger [44]. Inhibitor **12** is shown in cyan. The color coding of the binding sites has been generated based on the proximity of the molecular surface to the corresponding P5 to P1' residues of the peptide substrate shown in 4D. The size and location of the colored regions of the S5 to S1' sites, therefore, depend on the size and position of the side chains of the peptide. (C) Superpositions of the protease‐bound inhibitors **12** (cyan) and Gü3899 (white; structure in Figure 1; PDB ID 8RJY) [26]. (D) Superposition of the binding modes of inhibitor **12** (cyan) and the substrate peptide ATVRLQAGNA (white) bound to a C145A M^pro^ variant (PDB ID 7TA4) [11].

To characterize the binding mode of the potent inhibitor **12** and the arrangement of its residues within the active site, we next determined the crystal structure of M^pro^ bound to inhibitor **12** at 1.79 Å resolution (Figure 4A,B; Table S1; PDB ID 9SDM). In the complex, the warhead of **12** formed a covalent bond with the catalytically active cysteine residue (C145). The covalently attached inhibitor is well‐defined in the electron density maps, and the only indication of flexibility is two carbon atoms of the chlorophenyl ring that have weaker density, indicating some movement of this substituent. The chlorophenyl group is located in the S1' pocket, while the S1 pocket is not occupied by the inhibitor. The neighboring phenylalanine binds to the S2 pocket and the *tert*‐leucine to the S3 site. The terminal thiophene‐2‐carbonyl substituent occupies the S4 pocket. Hence, except for the residue at the P1 position, the P2–P4 fragments reside in the respective subsites accommodating the inhibitor in a substrate‐like manner along the active‐site cleft. This is further illustrated by a superposition with a peptide substrate bound to M^pro^ (PDB ID 7TA4) [11], highlighting similarity in the binding modes of the substrate and inhibitor P2–P4 backbones and in the orientation of the respective side chains (Figure 4D). Two hydrogen bonds are formed between the amide bonds of the *tert*‐leucine and the peptide bonds of E166, with the NH of *tert*‐leucine donating a hydrogen bond to the carbonyl oxygen of E166 and *vice versa*. The backbone NH group of C145 is hydrogen‐bonded to the C‐terminal carbonyl oxygen of the inhibitor. The side chain of H41 and the hydrazide NH group participate in a further hydrogen bond.

A carbonate (or hydrogencarbonate) ion is placed in the S1 pocket of the protease‐inhibitor complex, as indicated by a well‐defined trigonal planar electron density, which did not originate from the crystallization buffer and is most likely derived from carbon dioxide. The (hydrogen)carbonate ion superimposes closely with the carboxamide group of the glutamine side chain in the S1 pocket of a peptide substrate (Figure 4D).

The crystal structure of the M^pro^‐**12** complex is the first reported example of a crystallographic confirmation of the covalent binding mode of an inhibitor with a chloroacetohydrazide moiety in the active site of M^pro^. It verifies the nucleophilic displacement of chloride by the active‐site thiolate. Such a mechanism has already been suggested in 1999, based on mass spectrometry analysis of the complex of human rhinovirus 3C protease with a bromoacetohydrazide‐type inhibitor [45]. A bimolecular nucleophilic substitution occurred in the covalent attachment of a chlorofluoroacetohydrazide to the active site cysteine of M^pro^, whereupon chloride was replaced [31]. A related chloroacetyl warhead of non‐peptidic M^pro^ inactivators reacted similarly upon loss of chlorine [42, 46]. Recently, the chloroacetohydrazide warhead was established for the covalent inhibition of ubiquitin C‐terminal hydrolase L1 [47].

When comparing the crystallographic orientation of **12** and its close analog Gü3899, an almost identical overlay of the P2–P4 substructures was observed. However, the chlorobenzyl residues are accommodated by different binding sites (Figure 4C), which is likely attributed to the different position and hybridization of the nucleophilically attacked carbon atoms.

### 2.6 Cellular Antiviral Activity of Compound 12

**Figure 5:**
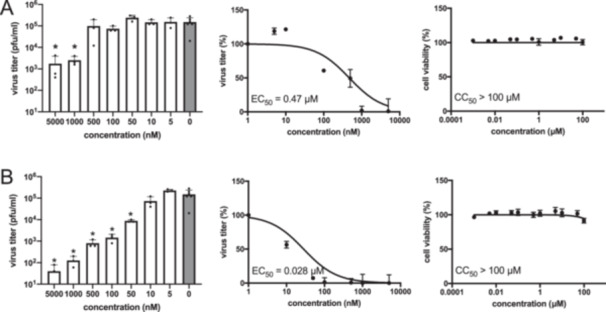
Antiviral activity against SARS‐CoV‐2 and cytotoxicity of (A) **12** and (B) reference compound ensitrelvir in A549‐ACE2 cells.

To examine the cellular efficacy of lead compound **12** against SARS‐CoV‐2 M^pro^, experiments were conducted in A549 cells expressing the angiotensin‐converting enzyme 2 (ACE2). This enzyme is a transmembrane receptor known to facilitate SARS‐CoV‐2 cell entry [48, 49]. Cells were infected at a multiplicity of infection (MOI) of 0.1, and quantification of the viral load was performed 24 h postinfection by means of a plaque assay. Ensitrelvir (structure in Figure 1) was employed as a positive control. With no cytotoxicity occurring up to 100 µM, **12** exhibited an EC_50_ value of 0.47 µM (Figure 5A). A reported EC_50_ value of nirmatrelvir in A549‐ACE2 cells is 0.054 µM [50]. For ensitrelvir, the obtained EC_50_ of 0.028 µM (Figure 5B) lies within the range of literature data from experiments employing the same cell line (0.004 and 0.089 µM) [15, 51]. Compared with the antiviral performance of the high‐potency positive control, the EC_50_ value of compound **12** is 17‐fold lower, but still in a submicromolar range without any cytotoxicity even at a concentration of 200‐fold EC_50_.

### 2.7 Stability of Compound 12 in Human Plasma

**Figure 6:**
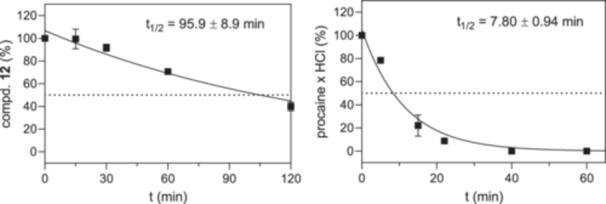
Determination of the plasma half‐lives of M^pro^ inhibitor **12** (left) and of the reference compound procaine hydrochloride (right). Data are means from duplicate experiments. The standard errors were obtained by propagation of uncertainty for the standard error of the first‐order rate constant of degradation *k* from non‐linear regression. In most cases, standard errors were smaller than the symbols.

For the investigation of the in vitro stability of **12** in human plasma, 100 µM of the M^pro^ inhibitor was incubated with male human plasma at 37°C for five different time periods. After quenching the enzymatic reactions, the residual proportion of **12** was quantified via HPLC and plotted *versus* incubation time to determine a plasma half‐life of 95.9 ± 8.9 min (Figure 6). Procaine hydrochloride served as a positive control, showing a half‐life of 7.80 ± 0.94 min, which confirmed sufficient plasma activity. A previous study, applying the same assay conditions, characterized Gü3899 by a plasma half‐life of 114 min and other structurally related azapeptide inhibitors by *t*_1/2_ values in the range of 77.1**–**99.3 min [26], underscoring the adequate stability of **12**. Nirmatrelvir was pharmacokinetically evaluated in a phase I study, exhibiting a plasma half‐life of 122 min after single‐dose treatment under fasted conditions without ritonavir coadministration [52].

## 3 Conclusion

We followed a sequential approach to create new peptidomimetic inhibitors of the main protease of SARS‐CoV‐2. In the course of a warhead hopping, suitable cysteine‐focused electrophiles were identified from a selection of small molecule analogs. Upon introducing efficacious warheads into a qualified tripeptide, compound **12** with a chloroacetohydrazide substructure outperformed the other inhibitor candidates. The highly potent (*k*_inac_/*K*_i_ = 78,900 M^–1^s^–1^) agent **12** was biochemically characterized as an irreversible inhibitor of M^pro^. Its kinetic behavior was in accordance with the covalent complex between M^pro^ and **12**, whose structure was solved by X‐ray crystallography. The protease–inhibitor interaction was elucidated to be based on the nucleophilic attack by the active‐site thiolate, the displacement of the chloride leaving group, and hence the establishment of a thioether linkage. Inhibitor **12** exhibited antiviral activity in the triple‐digit nanomolar range, determined in human lung adenocarcinoma cells, accompanied by a favorable stability and safety profile.

## 4 Experimental

### 4.1 Chemistry

#### 4.1.1 General Materials and Methods

For preparative column chromatography, silica gel with a pore size of 60 Å (0.063–0.200 mm) was used. An Interchim SAS (Montluçon, France) puriFlash XS520Plus was used for flash chromatography. Thin‐layer chromatography was conducted on silica gel 60 F_254_ aluminum sheets. The determination of melting points was performed on a Büchi (Essen, Germany) M‐560 apparatus. An Agilent (Santa Clara, CA, USA) HPLC 1260 Infinity II with EC50/2 Nucleodur C18 Gravity 3 µm column (Macherey‐Nagel, Düren, Germany) coupled to an Agilent Infinity Lab LC/MDS‐system with ESI‐source was applied for LC‐MS analyses. Samples were dissolved (1 mg/mL) in MeOH containing 2 mM ammonium acetate. The column temperature was set to 40°C, and the injection volume was 2 µL of the sample solution. Elution followed a gradient of 90% 2 mM ammonium acetate in H_2_O to 100% MeCN within 10 min and a flush with 100% MeCN for 10 min at a flow rate of 0.5 mL/min. Detection of UV absorption was conducted with a diode array detector from 190 to 600 nm, and the purity of the tested compounds was determined at 220–600 nm. Positive and negative total ion scans were recorded from 100 to 1000 *m/z*, and if necessary for a broader mass range. ^1^H NMR (500 MHz or 600 MHz), ^13^C NMR (126 MHz or 151 MHz), and ^19^F NMR (471 MHz or 565 MHz) spectra were recorded on a Bruker (Rheinstetten, Germany) DRX 500 MHz Avance or a Bruker Ascend 600 at 300–303 K using DMSO‐*d*_6_ as solvent. Chemical shift values *δ* are given in ppm and referenced to solvent signals (DMSO‐*d*_6_ 2.50/39.70 ppm). Coupling constants *J* are expressed in Hertz and spin multiplicities as singlet (s), doublet (d), doublet of doublets (dd), triplet (t), quartet (q), or multiplet (m). The data were processed and analyzed with MestReNova. High resolution mass spectra (HRMS) were obtained on a Thermo Fisher Scientific Orbitrap XL or on a Bruker micrOTOF‐Q mass spectrometer with ESI‐source, both coupled to a Thermo Fisher Scientific HPLC Dionex Ultimate 3000. Acetonitrile or a mixture of MeCN/H_2_O (9:1), containing 2 mM ammonium acetate, was applied as mobile phase. Positive full scan MS were recorded from 100 to 2000 or 50–1000 *m/z*.

#### 4.1.2 Compounds

Methyl (*E*)‐3‐{2‐[({benzyloxy}carbonyl)‐l‐phenylalanyl]‐1‐methylhydrazineyl}acrylate (**1**). Compound **15** (200 mg, 1.0 eq, 611 µmol), methyl propiolate (77 mg, 1.5 eq, 916 µmol, 82 µL), and glacial acetic acid (15 mg, 0.4 eq, 244 µmol, 14 µL) were stirred in anhydrous 1,4‐dioxane (10 mL) for 16 h at 60°C under a nitrogen atmosphere in a sealed system. The mixture was diluted with CH_2_Cl_2_ and water (50 mL each). The phases were separated, and the aqueous layer was extracted with CH_2_Cl_2_ (2 × 50 mL). The combined organic layers were dried over Na_2_SO_4_, filtered, and concentrated under reduced pressure. Purification by flash chromatography (cyclohexane/EtOAc 80:20 → 30:70) afforded the product as a white resin. Yield: 139 mg (54%); *R*_f_ = 0.45 (petroleum ether/EtOAc 1:2); ^1^H NMR (500 MHz, DMSO‐*d*_6_) *δ* 2.79–3.02 (m, 5H), 3.53 (s, 3H), 4.15–4.24 (m, 1H), 4.58 (d, *J* = 13.0 Hz, 1H), 4.92–5.01 (m, 2H), 7.17–7.37 (m, 13H); ^13^C NMR {^1^H} (126 MHz, DMSO‐*d*_6_) δ 37.1, 50.0, 54.7, 65.4, 85.9, 126.4, 127.6, 127.7, 128.1, 128.2, 129.2, 136.9, 137.3, 151.9, 155.8, 168.1, 169.8, one signal was obscured by the DMSO signal; LCMS (ESI), *t*_R_ = 6.30 min, 98% purity, *m/z* calculated for C_22_H_25_N_3_O_5_ [M + H]^+^, 412.2; found 412.4. HRMS (ESI) *m/z* calculated for C_22_H_25_N_3_O_5_ [M + H]^+^, 412.1867; found, 412.1868.

Dimethyl 2‐{2‐[({benzyloxy}carbonyl)‐l‐phenylalanyl]‐1‐methylhydrazineyl}maleate (**2**). Compound **15** (200 mg, 1.0 eq, 611 µmol), dimethyl but‐2‐ynedionate (BLD Pharma) (130 mg, 1.5 eq, 916 µmol, 113 µL), and glacial acetic acid (15 mg, 0.4 eq, 244 µmol, 14 µL) were stirred in anhydrous 1,4‐dioxane (10 mL) for 20 h at 60°C under a nitrogen atmosphere in a sealed system. The solution was diluted with CH_2_Cl_2_ and water (50 mL each), the phases were separated, and the aqueous layer was extracted with CH_2_Cl_2_ (2 × 50 mL). The combined organic layers were dried over Na_2_SO_4_, filtered, and concentrated under reduced pressure. Purification by flash chromatography (cyclohexane/EtOAc 80:20 → 30:70) afforded the product as a white solid. Yield: 227 mg (79%); *R*_f_ = 0.18 (CH_2_Cl_2_/MeOH 19:1); mp 142°C–145°C; ^1^H NMR (600 MHz, DMSO‐*d*_6_) *δ* 2.72–3.00 (m, 5H), 3.52 (s, 3H), 3.69 (s, 3H), 4.15–4.22 (m, 1H), 4.74 (s, 1H), 4.91–5.00 (m, 2H), 7.16–7.37 (m, 10H), 7.71 (s, 1H), 10.66 (s, 1H); ^13^C NMR {^1^H} (151 MHz, DMSO‐*d*_6_) *δ* 36.9, 50.5, 52.5, 54.6, 65.3, 85.5, 126.4, 127.5, 127.7, 128.1, 128.3, 129.2, 136.9, 137.4, 155.8, 163.7, 166.4, two signals were obscured the by DMSO signal; LCMS (ESI), *t*_R_ = 6.61 min, > 99% purity, *m/z* calculated for C_24_H_27_N_3_O_7_ [M + H]^+^, 470.6; found 470.6. HRMS (ESI) *m/z* calculated for C_24_H_27_N_3_O_7_ [M + H]^+^, 470.1922; found, 470.1922.

Ethyl 2‐{[(*N*‐benzyloxy)carbonyl]‐l‐phenylalanyl}‐1‐5;methylhydrazine‐1‐carboxylate (**3**). Compound **15** (200 mg, 1.0 eq, 611 µmol) was stirred in 10 mL anhydrous THF at 0°C. *N*‐Methylmorpholine (68 mg, 1.1 eq, 672 µmol, 74 µL) and ethyl chloroformate (73 mg, 1.1 eq, 672 µmol, 64 µL) were added at 0°C, and the mixture was stirred for 2 h at ambient temperature. The solution was diluted with CH_2_Cl_2_ and water (50 mL each), the phases were separated, and the aqueous layer was extracted with CH_2_Cl_2_ (2 × 50 mL). The combined organic layers were dried over Na_2_SO_4_, filtered, and concentrated under reduced pressure. The crude material was purified by flash chromatography (cyclohexane/EtOAc 80:20 → 30:70) to obtain the product as a white solid. Yield: 136 mg (56%); *R*_f_ = 0.31 (CH_2_Cl_2_/MeOH 19:1); mp 109°C–110°C ^1^H NMR (500 MHz, DMSO‐*d*_6_) *δ* 1.02–1.27 (m, 3H), 2.74–2.83 (m, 1H), 2.86–3.16 (m, 4H), 3.87–4.16 (m, 2H), 4.25 (s, 1H), 4.88–5.00 (m, 2H), 7.16–7.35 (m, 10H), 7.56–7.67 (m, 1H), 10.36 (s, 1H); ^13^C NMR {^1^H} (126 MHz, DMSO‐*d*_6_) *δ* 14.3, 37.1, 54.5, 61.4, 65.2, 126.3, 127.4, 127.6, 128.0, 128.2, 129.1, 136.9, 137.7, 155.7, 170.4, one signal was obscured by the DMSO signal; LCMS (ESI), *t*_R_ = 7.52 min, > 99% purity, *m/z* calculated for C_21_H_25_N_3_O_5_ [M + H]^+^, 400.2; found, 400.6. HRMS (ESI) *m/z* calculated for C_21_H_25_N_3_O_5_ [M + H]^+^, 400.1867; found 400.1866.

Methyl 3‐{2‐[({benzyloxy}carbonyl)‐l‐phenylalanyl]‐1‐6;methylhydrazineyl}‐3‐oxopropanoate (**4**). *Procedure A*: Compound **15** (200 mg, 1.0 eq, 611 µmol), methyl 3‐bromopropiolate (149 mg, 1.5 eq, 916 µmol), and glacial acetic acid (15 mg, 0.4 eq, 244 µmol, 14 µL) were stirred in anhydrous 1,4‐dioxane (15 mL) for 16 h at 60°C under a nitrogen atmosphere in a sealed system. The solution was diluted with CH_2_Cl_2_ and water (75 mL each), the phases were separated, and the aqueous layer was extracted with CH_2_Cl_2_ (2 × 75 mL). The combined organic layers were dried over Na_2_SO_4_, filtered, and concentrated under reduced pressure. The crude material was purified by flash chromatography (cyclohexane/EtOAc 80:20 → 0:100), followed by reversed‐phase flash chromatography (H_2_O/MeCN 70:30 → 40:60) to obtain the product as a colorless oil. Yield: 97 mg (32%); *R*_f_ = 0.23 (CH_2_Cl_2_/MeOH 19:1); ^1^H NMR (600 MHz, DMSO‐*d*_6_) *δ* 2.70–3.52 (m, 7H), 3.60 (s, 3H), 4.20 (s, 1H), 4.91–5.05 (m, 2H), 7.18–7.37 (m, 10H), 7.81 (d, *J* = 7.4 Hz, 1H), 10.60 (s, 1H); ^13^C NMR {^1^H} (151 MHz, DMSO‐*d*_6_) *δ* 34.8, 36.5, 51.8, 54.7, 65.4, 126.5, 127.6, 127.7, 128.2, 128.3, 129.2, 136.8, 137.2, 155.9, 167.6, 170.8, one signal was obscured by the DMSO signal; LCMS (ESI), *t*_R_ = 6.17 min, *m/z* calculated for C_22_H_25_N_3_O_6_ [M + H]^+^, 428.2; found, 428.7. HRMS (ESI) *m/z* calculated for C_22_H_25_N_3_O_6_ [M + H]^+^, 428.1816; found, 428.1818.

*Procedure B*: Compound **15** (200 mg, 1.0 eq, 611 µmol) was stirred in anhydrous CH_2_Cl_2_ (10 mL) and DIPEA (158 mg, 2.0 eq, 1.22 mmol, 213 µL). Methyl malonyl chloride (100 mg, 1.2 eq, 733 µmol, 79 µL) was added dropwise to the mixture, and the reaction was stirred for 20 h at ambient temperature. The solution was diluted with CH_2_Cl_2_ and water (50 mL each), the phases were separated, and the aqueous layer was extracted with CH_2_Cl_2_ (2 × 50 mL). The combined organic layers were dried over Na_2_SO_4_, filtered, and concentrated under reduced pressure. Purification by flash chromatography (cyclohexane/EtOAc 80:20 → 20:80) afforded the product as a colorless oil. Yield: 111 mg (42%); ^1^H NMR (600 MHz, DMSO‐*d*_6_) *δ* 2.64–3.53 (m, 7H, partly obscured by the water signal), 3.54–3.73 (m, 3H), 4.21 (s, 1H), 4.85–5.12 (m, 2H), 7.11–7.48 (m, 10H), 7.81 (d, *J* = 7.4 Hz, 1H), 10.60 (s, 1H); ^13^C NMR {^1^H} (151 MHz, DMSO‐*d*_6_) *δ* 34.8, 36.5, 51.8, 54.7, 65.4, 126.5, 127.6, 127.7, 128.2, 128.3, 129.2, 136.8, 137.2, 155.9, 167.6, 170.8, one signal was obscured by the DMSO signal; LCMS (ESI), *t*_R_ = 5.95 min, 99% purity, m/z calculated for C_22_H_25_N_3_O_6_ [M + H]^+^, 428.2; found, 428.4.

Ethyl (*E*)‐4‐{2‐[({benzyloxy}carbonyl)‐l‐phenylalanyl]‐1‐methylhydrazineyl}‐4‐oxobut‐2‐enoate (**5**). Monoethyl fumarate (106 mg, 1.2 eq, 733 µmol) was dissolved in anhydrous THF (8 mL) at 0°C. *N*‐Methylmorpholine (74 mg, 1.2 eq, 733 µmol, 81 µL) and ethyl chloroformate (80 mg, 1.2 eq, 733 µmol, 70 µL) were added, and the mixture was stirred for 15 min at 0°C. Compound **15** (200 mg, 1.0 eq, 611 µmol), dissolved in anhydrous THF (20 mL) and DIPEA (158 mg, 2.0 eq, 1.22 mmol, 213 µL), was added to the stirring reaction, and the mixture was stirred for 20 h at ambient temperature. The solution was diluted with CH_2_Cl_2_ and water (75 mL each), the phases were separated, and the aqueous layer was extracted with CH_2_Cl_2_ (2 × 50 mL). The combined organic layers were dried over Na_2_SO_4_, filtered, and concentrated under reduced pressure. Purification by flash chromatography (cyclohexane/EtOAc 80:20 → 30:70) afforded the product as a white solid. Yield: 172 mg (61%); *R*_f_ = 0.21 (CH_2_Cl_2_/MeOH 19:1); mp 139°C–142°C; ^1^H NMR (500 MHz, DMSO‐*d*_6_) *δ* 1.19 (t, *J* = 7.1 Hz, 3H), 2.69–3.14 (m, 5H), 4.13 (q, *J* = 6.3 Hz, 2H), 4.22–4.32 (m, 1H), 5.00 (s, 2H), 6.60 (d, *J* = 15.5 Hz, 1H), 7.10–7.41 (m, 11H), 7.82 (s, 1H), 10.82 (s, 1H); ^13^C NMR {^1^H} (126 MHz, DMSO‐*d*_6_) *δ* 13.9, 35.3, 36.6, 54.9, 60.7, 65.4, 126.5, 127.6, 127.7, 128.2, 128.2, 129.2, 131.0, 132.6, 136.8, 137.2, 155.9, 164.7, 165.5, 171.1; LCMS (ESI), *t*_R_ = 6.71 min, 99% purity, *m/z* calculated for C_24_H_27_N_3_O_6_ [M + H]^+^, 454.2; found, 454.5. HRMS (ESI) *m/z* calculated for C_24_H_27_N_3_O_6_ [M + H]^+^, 454.1973; found, 454.1973.

Benzyl (*S*)‐{1‐[2‐(2‐chloroacetyl)‐2‐methylhydrazineyl]‐1‐oxo‐3‐phenylpropan‐2‐yl}carbamate (**6**). Compound **15** (200 mg, 1.0 eq, 611 µmol) was stirred in anhydrous CH_2_Cl_2_ (10 mL) and DIPEA (158 mg, 2.0 eq, 1.22 mmol, 213 µL). Chloroacetyl chloride (83 mg, 1.2 eq, 733 µmol, 58 µL) was added, and the mixture was stirred for 2 h at ambient temperature. The solution was diluted with CH_2_Cl_2_ and water (50 mL each), the phases were separated, and the aqueous layer was extracted with CH_2_Cl_2_ (2 × 50 mL). The combined organic layers were dried over Na_2_SO_4_, filtered, and concentrated under reduced pressure. The crude material was purified by flash chromatography (cyclohexane/EtOAc 80:20 → 30:70), followed by reversed‐phase flash chromatography (H_2_O/MeCN 70:30 → 20:80) to yield the product as a white solid. Yield: 81 mg (32%); *R*_f_ = 0.27 (CH_2_Cl_2_/MeOH 19:1); mp 122°C–124°C; ^1^H NMR (600 MHz, DMSO‐*d*_6_) *δ* 2.70–3.04 (m, 5H), 3.45–4.46 (m, 3H), 4.84–5.14 (m, 2H), 7.06–7.50 (m, 10H), 7.85 (d, *J* = 7.0 Hz, 1H), 10.60 (s, 1H); ^13^C NMR {^1^H} (151 MHz, DMSO‐*d*_6_) *δ* 35.4, 36.4, 41.6, 54.8, 65.5, 126.6, 127.6, 127.8, 128.2, 128.3, 129.3, 136.8, 137.0, 156.0, 171.0, one signal was not visible; LCMS (ESI), *t*_R_ = 6.26 min, 96% purity, *m/z* calculated for C_20_H_22_ClN_3_O_4_ [M – H]^−^, 402.1; found, 402.2. HRMS (ESI) *m/z* calculated for C_20_H_22_ClN_3_O_4_ [M + H]^+^, 404.1372; found, 404.1370.

Benzyl (*S*)‐{1‐[2‐(2,2‐dichloroacetyl)‐2‐methylhydrazineyl]‐1‐oxo‐3‐phenylpropan‐2‐yl}carbamate (**7**). A mixture of compound **15** (200 mg, 1.0 eq, 611 µmol) and dichloroacetic anhydride (176 mg, 1.2 eq, 733 mmol, 112 µL) was stirred in anhydrous CH_2_Cl_2_ (10 mL) and DIPEA (158 mg, 2.0 eq, 1.22 mmol, 213 µL) for 18 h at ambient temperature. The solution was diluted with CH_2_Cl_2_ and water (50 mL each), the phases were separated, and the aqueous layer was extracted with CH_2_Cl_2_ (2 × 50 mL). The combined organic layers were dried over Na_2_SO_4_, filtered, and concentrated under reduced pressure. The crude material was purified by flash chromatography (cyclohexane/EtOAc 80:20 → 30:70) to yield the product as a white solid. The compound was obtained as a mixture of two rotamers. Yield: 49 mg (18%); *R*_f_ = 0.22 (CH_2_Cl_2_/MeOH 19:1); mp 163°C–167°C; ^1^H NMR (600 MHz, DMSO‐*d*_6_) *δ* 2.73–3.09 (m, 5H), 4.10–4.38 (m, 1H), 4.87–5.20 (m, 2H), 6.05–6.91 (m, 1H), 7.11–7.49 (m, 10H), 7.78–8.02 (m, 1H), 10.57–10.98 (m, 1H); ^13^C NMR {^1^H} (151 MHz, DMSO‐*d*_6_) *δ* 35.8, 36.2, 54.9, 63.8, 65.6, 126.6, 127.7, 127.8, 128.2, 128.3, 129.3, 136.7, 136.9, 156.3, 165.5, 171.2; LCMS (ESI), *t*_R_ = 6.89 min, 99% purity, *m/z* calculated for C_20_H_21_Cl_2_N_3_O_4_ [M – H]^−^, 436.1; found, 436.3. HRMS (ESI) *m/z* calculated for C_20_H_21_Cl_2_N_3_O_4_ [M + H]^+^, 438.0982; found, 438.0969.

Benzyl {[2*S*]‐1‐[2‐(2‐chloro‐2‐fluoroacetyl)‐2‐methylhydrazinyl]‐1‐oxo‐3‐phenylpropan‐2‐yl}carbamate (**8**). To a stirring solution of compound **15** (150 mg, 1.0 eq, 458 µmol), compound **16** (68 mg, 1.1 eq, 504 µmol), and DIPEA (178 mg, 3.0 eq, 1.37 mmol, 239 µL) in anhydrous DMF (10 mL), HATU (192 mg, 1.1 eq, 504 µmol) was added at 0°C under a nitrogen atmosphere. The corresponding mixture was stirred for 18 h at ambient temperature. All volatiles were removed under reduced pressure, and the residue was partitioned between CH_2_Cl_2_ and water (50 mL each). The aqueous layer was adjusted to pH 10–12 with aqueous saturated NaHCO_3_ solution, and the phases were separated. The aqueous layer was extracted with CH_2_Cl_2_ (2 × 50 mL). The combined organic layers were dried over Na_2_SO_4_, filtered, and concentrated under reduced pressure. The crude material was purified by a twofold flash chromatography (I: cyclohexane/EtOAc 90:10 → 40:60; II: CH_2_Cl_2_/MeOH 100:0 → 98:2) to yield the compound as a white solid. The compound was obtained as a mixture of two diastereomers with two rotamers each. Yield: 53 mg (27%); *R*_f_ = 0.27 (petroleum ether/EtOAc 1:1); mp 159°C–162°C; ^1^H NMR (500 MHz, DMSO‐*d*_6_) 2.65–3.16 (m, 5H), 4.02–4.42 (m, 1H), 4.83–5.15 (m, 2H), 5.78–8.26 (m, 12H), 10.39–11.04 (m, 1H);^13^C NMR {^1^H} (126 MHz, DMSO‐*d*_6_) *δ* 35.4, 36.3, 54.9, 65.6, 90.0 (d, *J* = 248.2 Hz), 126.5, 127.6, 127.8, 128.2, 128.3, 129.2, 136.7, 137.0, 156.0, 171.9, one signal was not visible; ^19^F NMR (471 MHz, DMSO‐*d*_6_, major diastereomer, major rotamer) *δ* ‐148.1 (d, *J* = 50.3 Hz); LC‐MS (ESI), *t*_R_ = 6.66 min, 99% purity, *m/z* calculated for C_20_H_21_ClFN_3_O_4_ [M − H]^−^, 420.1; found, 420.0. HRMS (ESI) *m/z* calculated for C_20_H_21_ClFN_3_O_4_ [M + H]^+^, 422.1277; found, 422.1266.

Benzyl (*S*)‐{1‐[2‐(2‐chloro‐2,2‐difluoroacetyl)‐2‐methylhydrazineyl]‐1‐oxo‐3‐phenylpropan‐2‐yl}carbamate (**9**). HATU (192 mg, 1.1 eq, 504 µmol) was added to a stirring solution of compound **15** (150 mg, 1.0 eq, 458 µmol), sodium 2‐chloro‐2,2‐difluoroacetate (BLD Pharma) (77.0 mg, 1.1 eq, 504 µmol), and DIPEA (178 mg, 3.0 eq, 1.37 mmol, 239 µL) in 10 mL anhydrous DMF at 0°C under nitrogen atmosphere, and the corresponding mixture was stirred for 18 h at ambient temperature. All volatiles were removed under reduced pressure, and the residue was partitioned between CH_2_Cl_2_ and water (50 mL each). The aqueous layer was adjusted to pH 10–12 with aqueous saturated NaHCO_3_ solution, and the phases were separated. The aqueous layer was extracted with CH_2_Cl_2_ (2 × 50 mL), dried over Na_2_SO_4_, filtered, and removed under reduced pressure. The crude material was purified by a twofold flash chromatography (I: cyclohexane/EtOAc 90:10 → 40:60; II: CH_2_Cl_2_/MeOH 100:0 → 98:2), followed by reversed‐phase flash chromatography (H_2_O/MeCN 70:30 → 30:70) to yield the product as a colorless resin. Yield: 12 mg (6%); *R*_f_ = 0.65 (petroleum ether/EtOAc 1:1); ^1^H NMR (500 MHz, CDCl_3_) 2.96–3.08 (m, 4H), 3.09–3.17 (m, 1H), 4.37–4.56 (m, 1H), 4.93–5.13 (m, 2H), 5.19–5.44 (m, 1H), 7.12–7.20 (m, 2H), 7.21–7.26 (m, 3H, partly obscured by the chloroform signal), 7.26–7.36 (m, 5H, partly obscured by the chloroform signal), 8.56 (s, 1H); ^13^C NMR {^1^H} (151 MHz, DMSO‐*d*_6_) *δ* 37.1, 37.2, 54.8, 67.8, 118.1 (t, *J* = 301.6 Hz), 127.6, 128.1, 128.7, 128.8, 129.1, 129.4, 135.6, 135.7, 156.7, 159.5 (t, *J* = 29.5 Hz), 170.2; ^19^F NMR (471 MHz, CDCl_3_) *δ* –60.0; LC‐MS (ESI), *t*_R_ = 7.08 min, 99% purity, *m*/*z* calculated for C_20_H_20_ClF_2_N_3_O_4_ [M − H]^−^, 438.1; found, 438.0. HRMS (ESI) *m/z* calculated for C_20_H_20_ClF_2_N_3_O_4_ [M + Na]^+^, 462.1003; found, 462.1002.

Benzyl (*S*)‐{1‐[2‐(cyanomethyl)‐2‐methylhydrazineyl]‐1‐oxo‐3‐phenylpropan‐2‐yl}carbamate (**10**). A mixture of compound **15** (150 mg, 1.0 eq, 458 µmol), Cs_2_CO_3_ (299 mg, 2.0 eq, 916 µmol), sodium iodide (343 mg, 5.0 eq, 2.29 mmol), and 2‐chloroacetonitrile (38.0 mg, 1.1 eq, 504 µmol, 32 µL) in 20 mL anhydrous acetone was stirred for 5 h at 75°C under reflux. The mixture was cooled down to ambient temperature, and all volatiles were removed under reduced pressure. The residue was partitioned between CH_2_Cl_2_ and water (50 mL each). The phases were separated, and the aqueous layer was extracted with CH_2_Cl_2_ (2 × 50 mL). The combined organic layers were dried over Na_2_SO_4_, filtered, and concentrated under reduced pressure. The crude material was purified by flash chromatography (CH_2_Cl_2_/MeOH 99:1 → 94:6), followed by reversed‐phase flash chromatography (H_2_O/MeCN 90:10 → 80:20) to yield the product as a white solid. Yield: 54 mg (32%); *R*_f_ = 0.22 (CH_2_Cl_2_/MeOH 19:1); mp 151°C–153°C; ^1^H NMR (500 MHz, DMSO‐*d*_6_) 2.19–2.68 (m, 3H, partly obscured by the DMSO signal), 2.71–2.82 (m, 1H), 2.83–3.00 (m, 1H), 3.62–4.02 (m, 2H), 4.09–5.04 (m, 3H), 6.98–7.71 (m, 11H), 8.70–9.63 (m, 1H); ^13^C NMR {^1^H} (126 MHz, DMSO‐*d*_6_) *δ* 37.7, 43.2, 45.6, 54.7, 65.2, 116.3, 126.3, 127.4, 127.6, 128.0, 128.2, 129.2, 137.0, 137.6, 155.7, 169.3; LC‐MS (ESI), *t*_R_ = 5.81 min, 96% purity, *m*/*z* calculated for C_20_H_22_N_4_O_3_ [M + H]^+^, 367.2; found, 367.2. HRMS (ESI) *m/z* calculated for C_20_H_22_N_4_O_3_ [M + H]^+^, 367.1765; found, 367.1746.

Benzyl (*S*)‐{1‐[2‐(fluorosulfonyl)‐2‐methylhydrazineyl]‐1‐oxo‐3‐phenylpropan‐2‐yl}carbamate (**11**). To a stirring solution of compound **15** (200 mg, 1.0 eq, 611 µmol) in 10 mL anhydrous CH_2_Cl_2_ (10 mL), 1‐(fluorosulfonyl)‐2,3‐dimethyl‐1*H*‐imidazol‐3‐ium trifluoromethanesulfonate (BLD Pharma) (201 mg, 1.0 eq, 611 µmol) was added at 0°C. The mixture was stirred for 4.5 h at ambient temperature. The solution was diluted with CH_2_Cl_2_ and water (50 mL each), and the aqueous layer was adjusted to pH 2 with aqueous 10% KHSO_4_ solution. The phases were separated, and the aqueous layer was extracted with CH_2_Cl_2_ (2 × 50 mL). The combined organic layers were dried over Na_2_SO_4_, filtered, and concentrated under reduced pressure. Purification by flash chromatography (cyclohexane/EtOAc 80:20 → 0:100) afforded the product as a white solid. Yield: 85 mg (34%); *R*_f_ = 0.71 (CH_2_Cl_2_/MeOH 19:1); mp 219°C–221°C; ^1^H NMR (600 MHz, DMSO‐*d*_6_) *δ* 2.77–2.85 (m, 1H), 2.90–3.00 (m, 1H), 3.34 (s, 3H), 4.17–4.32 (m, 1H), 4.91–5.02 (m, 2H), 7.17–7.38 (m, 10H), 7.77 (d, *J* = 8.3 Hz, 1H), 11.07 (s, 1H); ^13^C NMR {^1^H} (151 MHz, DMSO‐*d*_6_) *δ* 36.9, 54.6, 65.4, 126.5, 127.5, 127.7, 128.1, 128.3, 129.2, 136.8, 137.2, 155.8, 170.7, one signal was obscured by the DMSO signal; ^19^F NMR (565 MHz, DMSO) *δ* 42.5–43.0 (m); HRMS (ESI) *m/z* calculated for C_18_H_20_FN_3_O_5_S [M + H]^+^, 410.1180; found, 410.1165.

*N*‐{[*S*]‐1‐[({*S*}‐1‐{2‐(2‐Chloroacetyl)‐2‐(3‐chlorobenzyl)hydrazineyl}‐1‐oxo‐3‐phenylpropan‐2‐yl)amino]‐3,3‐dimethyl‐1‐oxobutan‐2‐yl}thiophene‐2‐carboxamide (**12**). Hydrazide derivative **21** (200 mg, 1.0 eq, 379 µmol) was stirred in anhyd CH_2_Cl_2_ (10 mL) and DIPEA (98.0 mg, 2.0 eq, 759 µmol, 132 µL) at ambient temperature under a nitrogen atmosphere. 2‐Chloroacetyl chloride (51.0 mg, 1.2 eq, 455 µmol, 36 µL) was added, and the mixture was stirred for 18 h at ambient temperature. The solution was diluted with CH_2_Cl_2_ and water (50 mL each), the phases were separated, and the aqueous layer was extracted with CH_2_Cl_2_ (2 × 50 mL). The combined organic layers were dried over Na_2_SO_4_, filtered, and concentrated under reduced pressure. The crude material was purified by flash chromatography (CH_2_Cl_2_/MeOH 100:0 → 95:5) to yield the product as a white solid. Yield: 96 mg (41%); *R*_f_ = 0.55 (CH_2_Cl_2_/MeOH 9:1); mp 139°C–147°C; ^1^H NMR (500 MHz, DMSO‐*d*_6_) *δ* 0.96 (s, 9H), 2.74–3.07 (m, 2H), 3.15–5.23 (m, 6H, partly obscured by the water signal), 6.93–7.41 (m, 10H), 7.73–8.11 (m, 3H), 8.66 (s, 1H), 10.59–10.84 (m, 1H); ^13^C NMR {^1^H} (126 MHz, DMSO‐*d*_6_) *δ* 26.6, 34.5, 36.5, 42.2, 51.5, 53.1, 59.7, 126.7, 127.3, 127.7, 127.8, 128.2, 129.0, 129.1, 130.0, 131.1, 132.9, 136.5, 138.5, 139.3, 161.1, 167.8, 170.7, one signal (CO) was not visible; LCMS (ESI), *t*_R_ = 7.88 min, 98% purity, *m/z* calculated for C_29_H_32_Cl_2_N_4_O_4_S [M + H]^+^, 603.2; found, 603.1. HRMS (ESI) *m/z* calculated for C_29_H_32_Cl_2_N_4_O_4_S [M + H]^+^, 603.1594; found, 603.1577.

*N*‐{[*S*]‐1‐[({*S*}‐1‐{2‐(3‐Chlorobenzyl)‐2‐(2,2‐dichloroacetyl)hydrazineyl}‐1‐oxo‐3‐phenylpropan‐2‐yl)amino]‐3,3‐dimethyl‐1‐oxobutan‐2‐yl}thiophene‐2‐carboxamide (**13**). Hydrazide derivative **21** (200 mg, 1.0 eq, 379 µmol) was stirred in anhydrous CH_2_Cl_2_ (10 mL) and DIPEA (98.0 mg, 2.0 eq, 759 µmol, 132 µL) at ambient temperature under a nitrogen atmosphere. Dichloroacetic anhydride (109 mg, 1.2 eq, 455 µmol, 70 µL) was added, and the mixture was stirred for 18 h at ambient temperature. The solution was diluted with CH_2_Cl_2_ and water (50 mL each), the phases were separated, and the aqueous layer was extracted with CH_2_Cl_2_ (2 × 50 mL). The combined organic layers were dried over Na_2_SO_4_, filtered, and concentrated under reduced pressure. The crude material was purified by flash chromatography (CH_2_Cl_2_/MeOH 100:0 → 95:5) to yield the product as a white solid. Yield: 62 mg (25%); R_f_ = 0.60 (CH_2_Cl_2_/MeOH 9:1); mp 143°C–152°C; ^1^H NMR (500 MHz, DMSO‐*d*_6_) *δ* 0.99 (s, 9H), 2.72–3.11 (m, 2H), 3.59–5.13 (m, 4H), 5.41–6.92 (m, 1H), 6.96–7.45 (m, 10H), 7.69–8.20 (m, 3H), 8.55–8.88 (m, 1H), 10.80–11.18 (m, 1H); ^13^C NMR {^1^H} (126 MHz, DMSO‐*d*_6_) *δ* 26.6, 34.5, 36.2, 52.2, 53.2, 59.5, 64.0, 126.7, 127.5, 127.7, 127.8, 128.2, 129.1, 129.2, 130.1, 131.1, 132.9, 136.4, 137.8, 139.3, 161.1, 165.9, 171.0, 171.1; LCMS (ESI), *t*_R_ = 8.37 min, 99% purity, *m/z* calculated for C_29_H_31_Cl_3_N_4_O_4_S [M − H]^−^, 635.1; found, 635.2. HRMS (ESI) *m/z* calculated for C_29_H_31_Cl_3_N_4_O_4_S [M + H]^+^, 637.1204; found, 637.1191.

*N*‐{[2*S*]‐1‐[({2*S*}‐1‐{2‐(2‐Chloro‐2‐fluoroacetyl)‐2‐(3‐chlorobenzyl)hydrazineyl}‐1‐oxo‐3 phenylpropan‐2‐yl)amino]‐3,3‐dimethyl‐1‐oxobutan‐2‐yl}thiophene‐2‐carboxamide (**14**). Hydrazide derivative 21 (200 mg, 1.0 eq, 379 µmol) and compound **16** (56.0 mg, 1.1 eq, 417 µmol) were stirred in anhydrous DMF (10 mL) at 0°C under a nitrogen atmosphere. HATU (159 mg, 1.1 eq, 417 µmol) and DIPEA (147 mg, 3.0 eq, 1.14 mmol, 198 µL) were added at 0°C, and the mixture was stirred for 18 h at ambient temperature. All volatiles were removed under reduced pressure, and the residue was partitioned between CH_2_Cl_2_ and water (50 mL each). The aqueous layer was adjusted to pH 10–11 with aqueous saturated NaHCO_3_ solution, the phases were separated, and the aqueous layer was extracted with CH_2_Cl_2_ (2 × 50 mL). The combined organic layers were dried over Na_2_SO_4_, filtered, and concentrated under reduced pressure. Purification by flash chromatography (CH_2_Cl_2_/MeOH 100:0 → 95:5) afforded the product as a white solid. The compound was obtained as a diastereomeric mixture. Yield: 13 mg (5%); *R*_f_ = 0.61 (CH_2_Cl_2_/MeOH 9:1); mp 210°C–212°C; ^1^H NMR (600 MHz, DMSO‐d_6_) *δ* 0.89–1.02 (m, 9H), 2.77–3.09 (m, 2H), 3.60–5.14 (m, 4H), 6.88–7.38 (m, 11H), 7.72–7.79 (m, 1H), 7.84–8.13 (m, 2H), 8.60–8.79 (m, 1H), 10.85–11.13 (m, 1H); ^13^C NMR {^1^H} (151 MHz, DMSO‐*d*_6_) *δ* 26.5/26.6, 34.4/34.5, 36.2/36.6, 51.6, 53.1, 59.4/59.8, 88.9–91.1 (m, CHFCl), 126.7, 127.52, 127.76/127.84, 128.2, 129.1/129.2, 130.2, 131.1, 132.9, 136.4, 136.5, 137.6, 139.3, 161.1, 171.0, 171.6, one signal was not visible; *The compound was analyzed as diastereomeric mixture; LCMS (ESI), *t*_R_ = 8.18 min, 96% purity, *m/z* calculated for C_29_H_31_Cl_2_FN_4_O_4_S [M + H]^+^, 621.2; found, 621.2. HRMS (ESI) *m/z* calculated for C_29_H_31_Cl_2_FN_4_O_4_S [M + H]^+^, 621.1500; found, 621.1483.

Benzyl (*S*)‐[1‐(2‐methylhydrazineyl)‐1‐oxo‐3‐phenylpropan‐2‐yl]carbamate (**15**). [(Benzyloxy)carbonyl]‐l‐phenylalanine (TCI) (6.00 g, 1.0 eq, 20.1 mmol) was dissolved in anhydrous THF (60 mL). *N*‐Methylmorpholine (2.03 g, 1.0 eq, 20.1 mmol, 2.23 mL) and isobutyl chloroformate (2.74 g, 1.0 eq, 20.1 mmol, 2.61 mL) were added to the stirring solution at 0°C. Subsequently, methylhydrazine (4.62 g, 5.0 eq, 100 mmol, 5.25 mL) was added, and the reaction was stirred for 18 h at ambient temperature. All volatiles were removed under reduced pressure, and the residue was partitioned between ethyl acetate and water (150 mL each). The phases were separated, and the aqueous layer was extracted with ethyl acetate (2 × 75 mL). The combined organic layers were washed with brine (100 mL), dried over Na_2_SO_4_, filtered, and concentrated under reduced pressure. The crude material was purified by silica gel column chromatography (CH_2_Cl_2_/MeOH 39:1 → 29:1) to yield the compound as a white solid; mp 137°C–138°C, lit [23]. mp 138°C–139°C; Yield: 2.95 g (45%); *R*_f_ = 0.36 (CH_2_Cl_2_/MeOH 19:1); ^1^H NMR (600 MHz, DMSO) *δ* 2.30–2.40 (m, 3H), 2.74–2.83 (m, 1H), 2.86–2.96 (m, 1H), 4.13–4.19 (m, 1H), 4.73–4.80 (m, 1H), 4.90–4.98 (m, 2H), 7.16–7.35 (m, 10H), 7.50 (d, *J* = 8.6 Hz, 1H), 9.40–9.48 (m, 1H); ^13^C NMR {^1^H} (151 MHz, DMSO) *δ* 37.7, 38.3, 54.8, 65.2, 126.2, 127.4, 127.6, 128.0, 128.2, 129.2, 137.0, 137.8, 155.7, 169.8; LCMS (ESI), *t*_R_ = 5.41 min, 96% purity, *m/z* calculated for C_18_H_21_N_3_O_3_ [M + H]^+^, 328.2; found, 328.0.

Sodium 2‐chloro‐2‐fluoroacetate (**16**) [38]. Finely grounded NaOH (569 mg, 2.0 eq, 14.2 mmol) was added to a solution of ethyl 2‐chloro‐2‐fluoroacetate (BLD Pharm) (1.00 g, 1.0 eq, 7.12 mmol) in ethanol (15 mL) at 0°C, and the mixture was stirred for 30 min at 0°C. Subsequently, n‐hexane (30 mL) was added, and the mixture was stirred for 18 h at ambient temperature. The suspension was cooled down to 0°C, and the precipitate was filtered off and washed with n‐hexane (3 × 30 mL) to obtain the desired product as a white solid. Yield: 682 mg (71%); ^1^H NMR (500 MHz, DMSO‐*d*_6_) 6.04 (d, |^2^ *J*(H,F)| = 53.7 Hz, 1H); ^13^C NMR {^1^H} (126 MHz, DMSO‐*d*_6_) *δ* 95.5 (d, |^1^ *J*(C,F)| = 259.4 Hz, FClCHCO), 165.3 (d, |^2^ *J*(C,F)| = 20.2 Hz, FClCHCO); ^19^F NMR (471 MHz, DMSO‐*d*_6_) δ ‐132.48 (d, |^2^ *J*(F,H)| = 53.7 Hz).

Methyl {[S]‐2‐[(*tert*‐butoxycarbonyl)amino]‐3,3‐dimethylbutanoyl}‐l‐phenylalaninate (**17**). Boc‐l‐Tle‐OH (BLD Pharm) (10.3 g, 1.2 eq, 44.5 mmol), triethylamine (11.3 g, 3.0 eq, 111 mmol, 15.5 mL), and HATU (16.9 g, 1.2 eq, 44.5 mmol) were stirred in 50 mL anhydrous DMF for 20 min at 0°C under a nitrogen atmosphere. Methyl l‐phenylalaninate hydrochloride (BLD Pharm) (8.00 mmol) was suspended in anhydrous DMF (70 mL) and triethylamine (11.3 g, 3.0 eq, 111 mmol, 15.5 mL). The suspension was added to the stirring reaction, and the mixture was adjusted to pH 10 with triethylamine (3.75 g, 1.0 eq, 37.1 mmol, 5.20 mL). The reaction mixture was stirred for 18 h at ambient temperature. All volatiles were removed under reduced pressure, and the residue was partitioned between CH_2_Cl_2_ (100 mL) and water (200 mL). The aqueous layer was adjusted to pH 10 with 2 N aqueous KOH solution, the phases were separated, and the aqueous layer was extracted with CH_2_Cl_2_ (2 × 80 mL). Water (200 mL) was added to the combined organic layers. The aqueous layer was adjusted to pH 2 with an aqueous 10% KHSO_4_ solution, and the phases were separated. The organic layer was washed with brine (100 mL), dried over Na_2_SO_4_, filtered, and concentrated under reduced pressure. The crude material was purified by silica gel column chromatography (petroleum ether/EtOAc 67:33) to obtain the product as a white solid. Yield: 10.03 g (65%); *R*_f_ = 0.73 (petroleum ether/EtOAc 3:1); mp 135°C–138°C; lit [53]. mp 137°C–138°C; ^1^H NMR (500 MHz, DMSO‐*d*_6_) *δ* 0.84 (s, 9H), 1.38 (s, 9H), 2.89–2.97 (m, 1H), 3.00–3.07 (m, 1H), 3.56 (s, 3H), 3.89 (d, *J* = 9.7 Hz, 1H), 4.47–4.54 (m, 1H), 6.32 (d, *J* = 9.7 Hz, 1H), 7.16–7.28 (m, 5H), 8.29 (d, *J* = 7.4 Hz, 1H); ^13^C NMR {^1^H} (126 MHz, DMSO‐*d*_6_) *δ* 26.4, 28.1, 34.1, 36.5, 51.6, 53.3, 61.5, 78.0, 126.4, 128.1, 128.9, 137.0, 155.1, 170.3, 171.7; LCMS (ESI), *t*_R_ = 7.48 min, 95% purity, *m/z* calculated for fragment C_17_H_24_N_2_O_5_ [M − C_4_H_8_ + H]^+^, 337.2; found, 337.4.

Methyl [(*S*)‐3,3‐dimethyl‐2‐(thiophene‐2‐carboxamido)butanoyl]‐l‐phenylalaninate (**18**). Boc‐protected amine **17** (10.0 g, 1.0 eq, 25.5 mmol) was stirred in anhydrous CH_2_Cl_2_ and TFA (20 mL each) for 2 h at ambient temperature. All volatiles were evaporated under reduced pressure, co‐evaporated with CH_2_Cl_2_ (3 × 20 mL), and dried under high vacuum for 1 h. The deprotected amine, DMAP (3.42 g, 1.1 eq, 28.0 mmol) and triethylamine (7.73 g, 3.0 eq, 76.4 mmol, 10.7 mL) were stirred in anhydrous CH_2_Cl_2_ (170 mL) at 0°C under a nitrogen atmosphere. 2‐Thiophenecarbonyl chloride (4.11 g, 1.1 eq, 28.0 mmol, 3.00 mL) was added dropwise to the stirring mixture at 0°C, and the reaction was stirred for 18 h at ambient temperature. All volatiles were removed under reduced pressure, and the residue was partitioned between CH_2_Cl_2_ and water (100 mL each). The aqueous layer was adjusted to pH 11 with 2 N aqueous KOH, the phases were separated, and the aqueous layer was extracted with CH_2_Cl_2_ (2 × 80 mL). Water (150 mL) was added to the combined organic layers. The aqueous layer was adjusted to pH 2 with an aqueous 10% KHSO_4_ solution, and the phases were separated. The organic layer was dried over Na_2_SO_4_, filtered, and concentrated under reduced pressure. Purification by silica gel column chromatography (petroleum ether/EtOAc 75:25) afforded the product as a white solid. Yield: 7.86 g (76%); *R*_f_ = 0.36 (petroleum ether/EtOAc 3:1); mp 151°C–153°C; ^1^H NMR (600 MHz, DMSO‐*d*_6_) *δ* 0.95 (s, 9H), 2.91–2.98 (m, 1H), 2.99–3.06 (m, 1H), 3.55 (s, 3H), 4.46–4.53 (m, 2H), 7.10–7.15 (m, 2H), 7.16–7.22 (m, 4H), 7.77 (d, *J* = 5.0 Hz, 1H), 7.81 (d, *J* = 9.6 Hz, 1H), 7.97 (d, *J* = 3.7 Hz, 1H), 8.54 (d, *J* = 7.2 Hz, 1H); ^13^C NMR {^1^H} (151 MHz, DMSO‐*d*_6_) *δ* 26.6, 34.5, 36.5, 51.6, 53.5, 60.0, 126.4, 127.8, 128.1, 128.8, 129.0, 131.0, 137.0, 139.3, 160.9, 169.8, 171.6; LCMS (ESI), *t*_R_ = 6.82 min, 99% purity, *m/z* calculated for C_21_H_26_N_2_O_4_S [M + H]^+^, 403.2; found, 403.2.

*N*‐{[*S*]‐1‐[({*S*}‐1‐Hydrazineyl‐1‐oxo‐3‐phenylpropan‐2‐yl)amino]‐3,3‐dimethyl‐1‐oxobutan‐2‐yl}thiophene‐2‐carboxamide (**19**). Methyl ester derivative **18** (7.83 g, 1.0 eq, 19.5 mmol) and 65% hydrazine monohydrate solution (3.84 g, 4.0 eq, 77.8 mmol, 3.72 mL) were stirred in ethanol (50 mL) for 4.5 h at 95°C under reflux. The reaction was cooled down to room temperature and stirred for 16 h at ambient temperature. All volatiles were removed under reduced pressure, and the residue was partitioned between CH_2_Cl_2_ and water (100 mL each). The phases were separated, and the aqueous layer was extracted with CH_2_Cl_2_ (2 × 80 mL). The combined organic layers were dried over Na_2_SO_4,_ filtered, and concentrated under reduced pressure. The crude material was purified by silica gel column chromatography (petroleum ether/EtOAc 67:33 → 0:100 → EtOAC/EtOH 95:5) to yield the product as a white solid. Yield: 6.86 g (86%); *R*_f_ = 0.50 (100% EtOAc); mp 120°C–124°C; ^1^H NMR (600 MHz, DMSO‐*d*_6_) *δ* 0.89 (s, 9H), 2.77–2.85 (m, 1H), 2.91–2.97 (m, 1H), 4.20 (s, 2H), 4.45 (d, *J* = 9.5 Hz, 1H), 4.50–4.57 (m, 1H), 7.06–7.11 (m, 1H), 7.12–7.22 (m, 5H), 7.76–7.79 (m, 1H), 7.81 (d, *J* = 9.5 Hz, 1H), 7.92–7.95 (m, 1H), 8.17 (d, *J* = 8.3 Hz, 1H), 9.11 (s, 1H); ^13^C NMR {^1^H} (151 MHz, DMSO‐*d*_6_) *δ* 26.7, 34.3, 38.0, 52.4, 60.4, 126.1, 127.8, 127.9, 128.8, 129.1, 131.0, 137.5, 139.4, 160.9, 169.3, 169.9; LCMS (ESI), *t*_R_ = 5.12 min, 98% purity, *m/z* calculated for C_20_H_26_N_4_O_3_S [M + H]^+^, 403.2; found, 403.2.

*N*‐{[*S*]‐1‐[({*S*}‐1‐{2‐[(*E*)‐3‐Chlorobenzylidene]hydrazineyl}‐1‐oxo‐3‐phenylpropan‐2‐yl)amino]‐3,3‐dimethyl‐1‐oxobutan‐2‐yl}thiophene‐2‐carboxamide (**20**). Hydrazine derivative **19** (2.00 g, 1.0 eq, 4.97 mmol), Na_2_SO_4_ (3.00 g), and *m*‐chlorobenzaldehyde (768 mg, 1.1 eq, 5.47 mmol, 620 µL) were stirred in ethanol (50 mL) for 16 h at ambient temperature. All volatiles were removed under reduced pressure, and the residue was dried under high vacuum for 1 h. Purification by silica gel column chromatography (CH_2_Cl_2_/MeOH 100:0 → 92:8) afforded the product as a white solid. Yield: 2.53 g (95%); *R*_f_ = 0.20 (CH_2_Cl_2_/MeOH 19:1); mp 134°C–139°C; ^1^H NMR (500 MHz, DMSO‐*d*_6_) *δ* 0.85–0.99 (m, 9H), 2.86–2.96 (m, 1H), 2.99–3.09 (m, 1H), 4.43–4.56 (m, 1H), 4.60–5.52 (m, 1H), 7.03–7.29 (m, 6H), 7.40–7.51 (m, 2H), 7.55–7.67 (m, 1H), 7.69–7.86 (m, 3H), 7.89–8.18 (m, 2H), 8.37–8.45 (m, 1H), 11.43–11.63 (m, 1H); ^13^C NMR {^1^H} (126 MHz, DMSO‐*d*_6_) δ 26.6/26.7, 34.3/34.5, 36.9/37.4, 50.6/53.2, 60.0/60.6, 125.7/125.8, 126.23/126.27/126.31, 127.8, 127.9/128.0, 128.7, 128.9, 129.0/129.1, 129.4, 129.6, 130.6/130.7, 130.9/131.1, 133.6, 136.29/136.34, 137.3, 137.7, 139.2, 139.4, 141.5, 145.3, 160.8/161.1, 169.5/169.7, 167.5/172.4; *The compound was analyzed as mixture of *E*/*Z*‐isomers; LCMS (ESI), *t*_R_ = 7.74 min, 98% purity, *m/z* calculated for C_27_H_29_ClN_4_O_3_S [M + H]^+^, 525.2; found 525.7.

*N*‐{[*S*]‐1‐[({*S*}‐1‐{2‐[3‐Chlorobenzyl]hydrazineyl}‐1‐oxo‐3‐phenylpropan‐2‐yl)amino]‐3,3‐dimethyl‐1‐oxobutan‐2‐yl}thiophene‐2‐carboxamide (**21**). Hydrazone derivative **20** (2.50 g, 1.0 eq, 4.76 mmol) and *p*‐toluenesulfonic acid monohydrate (5.43 g, 6.0 eq, 28.6 mmol) were stirred in anhydrous CH_2_Cl_2_ (100 mL) and anhydrous methanol (60 mL) at 0°C. Borane dimethylamine complex (abcr) (449 mg, 1.6 eq, 7.62 mmol) was added to the stirring mixture at 0°C, and the reaction was stirred for 90 min at ambient temperature. After the addition of borane dimethylamine complex (449 mg, 1.6 eq, 7.62 mmol) for a second time, the reaction mixture was stirred for an additional 90 min. A third portion of borane dimethylamine complex (449 mg, 1.6 eq, 7.62 mmol) was added, and stirring of the reaction was continued for a further 16 h at ambient temperature. The reaction was quenched by the addition of 1.5 N aqueous NaOH solution (30 mL), and the resulting biphasic mixture was stirred for 30 min. The mixture was diluted with CH_2_Cl_2_ (50 mL) and water (80 mL), the phases were separated, and the aqueous layer was extracted with CH_2_Cl_2_ (2 × 70 mL). The combined organic layers were dried over Na_2_SO_4_, filtered, and concentrated under reduced pressure. The crude material was purified by flash chromatography (CH_2_Cl_2_/MeOH 98:2 → 92:8) to yield the product as white foam. Yield: 1.71 g (68%); *R*_f_ = 0.48 (CH_2_Cl_2_/MeOH 9:1); mp 96°C–101°C; ^1^H NMR (500 MHz, DMSO‐*d*_6_) *δ* 0.84–0.94 (m, 9H), 2.74–2.81 (m, 1H), 2.83–2.90 (m, 1H), 3.71–3.84 (m, 2H), 4.42–4.53 (m, 2H), 5.36 (q, *J* = 4.7 Hz, 1H), 7.05–7.11 (m, 1H), 7.12–7.22 (m, 6H), 7.25–7.35 (m, 3H), 7.74–7.82 (m, 2H), 7.92–7.97 (m, 1H), 8.19 (d, *J* = 8.2 Hz, 1H), 9.40 (d, *J* = 6.0 Hz, 1H); ^13^C NMR {^1^H} (126 MHz, DMSO‐*d*_6_) *δ* 26.7, 34.4, 37.8, 52.3, 53.6, 60.3, 126.1, 126.7, 126.9, 127.8, 127.9, 128.0, 128.8, 129.0, 129.8, 131.0, 132.8, 137.3, 139.3, 141.4, 160.9, 169.3, 169.7; LCMS (ESI), *t*_R_ = 7.38 min, 99% purity, *m/z* calculated for C_27_H_31_ClN_4_O_3_S [M + H]^+^, 527.2; found, 527.6.

### 4.2 Biochemical, Crystallographic, and Biological Experiments

#### 4.2.1 General Settings

A FluostarOptima plate reader (BMG Labtech, Ortenberg, Germany) and black 96‐well plates with a clear and flat bottom (Greiner Bio‐One, Kremsmünster, Austria) were used for the measurements, which were conducted at 37°C with *λ*_ex_ = 360 nm and *λ*_em_ = 460 nm. The expression and purification of the C‐terminally His_10_‐tagged M^pro^ followed a described protocol [25]. A buffer of 50 mM TRIS (pH 7.8), 100 mM NaCl, 1 mM EDTA, and 1 mM DTT was used to store the protease, which was freshly thawed for the experiments. The assay was conducted in 50 mM MOPS buffer, pH 7.2, containing 10 mM NaCl, 1 mM EDTA, and 0.01% (*v/v*) Triton X‐100 with a total volume of 50 µL per well, if not stated otherwise. DMSO was used as the solvent for the preparation of all stock solutions. Data analysis was performed by means of GraphPad Prism.

#### 4.2.2 Screening of **M^pro^** Inhibition at 10 and 50 µM

A 2.5 mM stock solution of the fluorogenic substrate Boc‐Abu‐Tle‐Leu‐Gln‐AMC was diluted with assay buffer (1 + 23) and pipetted into each well. A total of 1 µL of an inhibitor solution (or DMSO) was added, and the mixture was kept at 37°C for 5 min. To initiate the reaction, 25 µL of an enzyme dilution in assay buffer was added, and the reaction was monitored for 10 min. The final substrate concentration was 50 µM ( = 1.03 × *K*_m_) [25], the final inhibitor concentration was either 10 or 50 µM, the final DMSO content was 4% (*v*/*v*), the final DTT concentration was 20 µM, and the final concentration of active M^pro^ was 30 nM. The latter was adjusted by the employed volume of the enzyme solution, whose active‐site concentration was determined separately following a described procedure [40]. For smaller volumes of added M^pro^ solution, the DTT concentration was adjusted by adding storage buffer accordingly. The product formation rates at 10 or 50 µM inhibitor were normalized to the rate of the uninhibited control (100%). The resulting residual activity was subtracted from 100% to obtain the percentage inhibition after 10 min (*n *= 2). Compounds with ≥ 50% inhibition at 50 µM were further kinetically characterized.

#### 4.2.3 Investigation of Inhibition Reversibility by Jump Dilution

To 22.5 µL of an enzyme dilution in assay buffer, 2.5 µL of an inhibitor solution (or DMSO) was added. The mixture was incubated for 25 min at 37°C. The enzymatic reaction was started by adding 2 µL of the preincubated solution to a well containing 198 µL of a mixture of assay buffer, DMSO, DTT, and a 2.5 mM stock solution of a fluorogenic M^pro^ substrate. Product formation was monitored for 60 min. The total volume per well was 200 µL. The M^pro^ preincubation concentration was 500 nM, and the final M^pro^ concentration after jump dilution was 5 nM. The inhibitor preincubation concentration was ≈10 × IC_50_, and its concentration after dilution was ≈0.1 × IC_50_. The concentration of a tetrapeptidic, yet unpublished AMC substrate was 50 µM (=0.76 × *K*_m_), and DTT concentration corresponded to 100 µM during preincubation and 20 µM after dilution. The DMSO content was 10% (*v*/*v*) during preincubation and 5% (*v*/*v*) during the measurement. M^pro^ inhibition was considered irreversible if product formation after jump dilution was ≤ 10% of the uninhibited control (*n* = 2).

#### 4.2.4 Determination of Ic*_50_* and K*_i_* Values

Five different concentrations (c) corresponding to a range of *c*_1_ to 5 × c_1_ were employed for each inhibitor. The measurements were performed analogous to the M^pro^ inhibition screening, and any data on volumes, concentrations, and measurement conditions are as stated therein. The product formation rate at each inhibitor concentration was normalized to the rate of the uninhibited control (100%). The percentage residual activity was plotted *versus* inhibitor concentration. Non‐linear regression according to the equation *V*_i_ = *V*_0_/(1 + ([I]/IC_50_)) was performed to obtain IC_50_ (*V*_i_ = residual activity at a defined inhibitor concentration; *V*_0_ = activity in the absence of inhibitor; [*I*] = inhibitor concentration). *K*_i_ was calculated by fitting IC_50_ into the Cheng‐Prusoff equation (*n* = 2).

#### 4.2.5 Determination of K*_inac_*/K*_i_* for Irreversible Inhibitors

Five different concentrations corresponding to a range of c1 to 5 × c1 were employed for each inhibitor. The measurements were performed analogous to the M^pro^ inhibition screening, and any data on volumes and concentrations were as stated therein. The reactions were monitored for 60 min. The fluorescence readout was baseline‐corrected to correspond to the newly formed fluorescence, which is proportional to product formation. Non‐linear regression of ΔFU versus time was performed according to the equation ΔFU = *V*_0_ × ((1–*e*^ (–*k*_obs_ × *t*))/*k*_obs_) + *d* (*V*_0_ = initial reaction rate; *k*_obs_ = first‐order rate constant; *d* = offset). The obtained *k*_obs_ values were plotted *versus* inhibitor concentration. For non‐linear regression, the equation *k*_obs_ = (*k*_inac_ × [*I*])/([*I*] + *K*_i_ × (1 + [*S*]/*K*_m_)) was employed (*k*_inac_ = maximum rate of enzyme inactivation; *K*_i_ = inhibitor concentration at half‐maximum enzyme inactivation; [*S*] = substrate concentration; *K*_m_ = the Michaelis constant of the substrate). The second‐order rate constant of enzyme inactivation, *k*_inac_/*K*_i_, was obtained (*n* = 2).

#### 4.2.6 X‐Ray Crystal Structure Analysis

For crystallization, M^pro^ was overexpressed in BL21(DE3) *Escherichia coli* cells and purified as described [26, 41, 54]. The crystals were soaked with 250 µM of compound **12** for 24 h, similarly as described [55]. The buffer consisted of PEG 1500 (23.5%), MIB (malonic acid, imidazole, boric acid; 0.1 M, pH 7.7), 5% DMSO, 1 mM DTT, and 0.25 mM EDTA. The harvested crystal (without cryoprotectant) diffracted anisotropically to resolution limits between 2.25 and 1.56 Å (corresponding isotropic resolution limit 1.79 Å). X‐ray diffraction data were collected at 100 K at EMBL beamline P14 at the DESY synchrotron in Hamburg, Germany (Table S1). The diffraction data were indexed, integrated, and scaled with XDS [56] and STARANISO [57] as implemented in ISPyB [58] at DESY. The structure 7MBG [59, 60] was used as a starting model for refinement. Two protein chains are present in the asymmetric unit of the crystals, and the inhibitor was refined with full occupancy in both chains. Phenix [59] was used for refinement and Coot [61] for model building. Stereochemical restraints for ligand refinement were generated using grade2 (https://grade.globalphasing.org). Molecular figures were prepared using PyMOL (https://pymol.org).

#### 4.2.7 Determination of Cellular Antiviral Activity

##### 4.2.7.1 Cells and Virus

As described [21, 22], A549 cells overexpressing human angiotensin‐converting enzyme 2 (ACE2) (A549‐ACE2; kindly provided by Friedemann Weber, Institute of Virology, Justus Liebig University Giessen) were grown in Dulbecco's modified Eagle's medium (DMEM) supplemented with 10% fetal bovine serum (FBS) and antibiotics (100 U/mL penicillin and 100 μg/mL streptomycin) at 37°C in an atmosphere containing 5% CO_2_. In this study, the SARS‐CoV‐2 isolate Munich 929 was used, which was kindly provided by Christian Drosten (Institute of Virology, Charité‐Universitätsmedizin, Berlin) [62].

##### 4.2.7.2 Antiviral Activity

The antiviral activities of inhibitor **12** and ensitrelvir were investigated using protocols described previously [22, 63]. To determine effective concentrations 50% (EC_50_), A549‐ACE2 cells were inoculated with SARS‐CoV‐2 at an MOI of 0.1 plaque‐forming units (pfu) per cell. After incubation for 1 h at 33°C, the virus inoculum was replaced with fresh cell culture medium containing the test compounds at the indicated concentration. After 23 h at 33°C, the cell culture supernatants were collected, and virus titers were determined by virus plaque assay as described previously [64]. To calculate EC_50_ values, the virus titer determined for virus‐infected cells treated with DMSO only was set to 100% and titers obtained for treated cells were normalized to this value. EC_50_ values were calculated by non‐linear regression analysis using GraphPad Prism 6.0 (GraphPad Software).

##### 4.2.7.3 Cytotoxicity

Using previously described methods [65], the cytotoxic concentrations 50% (CC_50_) of **12** and ensitrelvir were determined by incubating cells, which were grown to near confluency in FCS‐free medium, with serial dilutions of **12** in a 96‐well format. After incubation for 24 h, 200 μL of MTT‐mix (DMEM supplemented with 10% FCS containing 250 μg/mL tetrazolium bromide, Sigma) was added to each well. Cells were further incubated for 90–120 min at 33°C and subsequently fixed with 3.7% paraformaldehyde in PBS. Tetrazolium crystals were dissolved by adding 200 μL isopropanol to each well, and absorbance at 490 nm was determined using an ELISA reader (BioTek). To determine the CC_50_, the MTT values were calculated in percent with the respective DMSO control set as 100%. CC_50_ values were calculated by non‐linear regression analysis using GraphPad Prism 5.0 (GraphPad Software).

#### 4.2.8 Determination of In Vitro Stability

A volume of 30 µL of a 2 mM compound stock solution in DMSO was mixed with 570 µL of a male human plasma solution (Biomol GmbH, Hamburg, Germany) that was diluted to 80% with 0.05 M sodium phosphate buffer, pH 7.4, and prewarmed at 37°C. The final compound concentration was 100 µM, and the DMSO concentration was 5% (*v*/*v*). The mixture was divided into aliquots of 100 µL, which were incubated for different time periods and subsequently quenched by the addition of 200 µL of a 0.05 mg/mL reserpine (internal standard) and 2% AcOH (*v*/*v*) solution in MeOH. The samples were vortexed for 1 min, stored on ice for 15 min, and subsequently centrifuged at 15,000 rpm and 4°C for 2 min. After filtration through a 0.45 µm polytetrafluoroethylene membrane filter, the resulting solutions were analyzed by HPLC (Jasco Deutschland GmbH, Pfungstadt, Germany). The AUC of the test compound was normalized to the reserpine AUC for each time of incubation, and the ratio at *t* = 0 was defined to equal a compound proportion of 100%. The residual proportion was plotted *versus* incubation time and non‐linear regression according to *Y*(*t*) = *Y*_0_ × *e*^ (‐*k*t) provided the first‐order rate constant of degradation *k*, with *Y*_0_ being the compound proportion at *t *= 0 and *Y(t*) the residual proportion after a specific incubation time *t*. Equation *t*_1/2_ = ln2/*k* afforded the plasma half‐life *t*_1/2_ (*n* = 2).

## Conflicts of Interest

The authors declare no conflicts of interest.

## Supporting information

RV_SI.

ArchPharm_SupplMat_InChI_2020.

## Data Availability

The data supporting the findings of this study can be found in the Supporting Information accompanying this article.
