# Supplementary material for: Sequential Optimization Approach Toward an Azapeptide‐Based SARS‐CoV‐2 Main Protease Inhibitor
Source: Arch Pharm (Weinheim). 2025 Dec 23;358(12):e70175. doi: 10.1002/ardp.70175 (PMC12723578; doi:10.1002/ardp.70175)
Supplement: Supplementary file 1 — RV_SI. [file ARDP-358-e70175-s002.pdf]

Supporting Information

Sequential Optimization Approach Towards an Azapeptide-Based SARS-CoV-2 Main Protease Inhibitor

Rabea Voget,[1] Victoria Steiger,[1] Julian Breidenbach,[1] Katharina Sylvester,[1] Christin Müller-Ruttloff,[2] Chun-Chiao Yang,[3] John Ziebuhr,[2] Norbert Sträter,[3] Christa E. Müller,[1] Michael Gütschow[1,*]

[1] Pharmaceutical Institute, Pharmaceutical & Medicinal Chemistry, University of Bonn, An der Immenburg 4, 53121 Bonn, Germany

[2] Institute of Medical Virology, Justus Liebig University Giessen, 35392 Giessen, Germany

[3] Institute of Bioanalytical Chemistry, Center for Biotechnology and Biomedicine, Leipzig University, 04103 Leipzig, Germany

*Corresponding Author: Michael Gütschow – guetschow@uni-bonn.de

Table of Contents

1. X-ray Crystallography Data…………………………………………………………… S3

TABLE S1 Diffraction data and refinement statistics………………………………….. S3

2. NMR Spectra………………………………………………………………………..…… S4

3. HRMS Spectra………………………………………….…………………………….. S30

4. LC-MS Traces………………………………………………………………..…….. S41

1. X-ray Crystallography Data

TABLE S1 Diffraction data and refinement statistics

Compound	Inhibitor **12** (GUE-4303)
PDB ID	9SDM
Final buffer before cryo-cooling	23.5% PEG 1500, 0.1 M MIB (malonic acid, imidazole, boric acid), 5% DMSO, 1 mM DTT, 0.25 mM EDTA
Data collection	
Source	DESY EMBL P14
Wavelength (Å)	1.3000
Resolution (Å)	72.74-1.55 (1.70-1.55)
Resolution iso (Å)	
Resolution aniso (Å)	1.555, 2.249, 1.576
Space group	$P2_12_12_1$
Unit cell dimensions (Å;°)	67.84, 101.73, 104.05; 90.0, 90.0 90.0
Unique reflections	66567 (3329)
Multiplicity	11.6 (5.9)
Completeness (%)* spherical/ellipsoidal	64.1 (13.7) / 92.1 (45.0)
Mean $I/\sigma(I)$	19.8 (1.8)
R-meas	0.064 (0.880)
R-merge	0.061 (0.803)
R-pim	0.018 (0.353)
$CC_{1/2}$	0.999 (0.686)
Wilson B (Å^2)	21.79
Refinement	
Resolution (Å)	36.37-1.55 (1.58-1.55)
R-work	0.1874 (0.3049)
R-free	0.2311 (0.2005)
Number of non-hydrogen atoms, B-value (Å^2)	
Protein	4713, 29.97
Heterogen	102, 33.40
Solvent	473, 34.33
Rmsd bonds (Å), angles (°)	0.012, 1.101
Ramachandran favored, allowed, outliers (%)	96.35, 3.65, 0.00
Rotamer favored, allowed, outliers (%)	94.89, 3.60, 1.52
MolProbity clashscore	3.28

2. NMR Spectra

Compound 1

^{1}H NMR (500 MHz, DMSO-d_6)

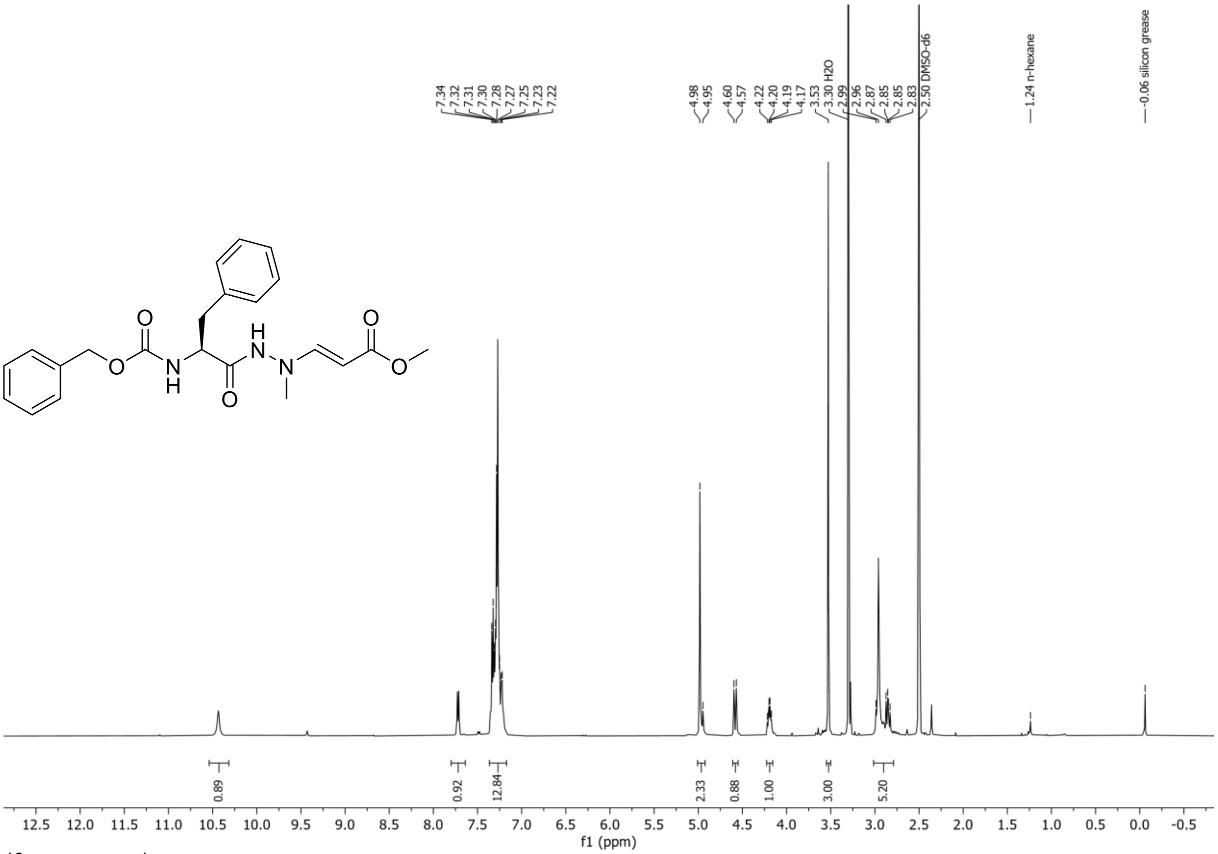

^{13}C NMR {^{1}H} (126 MHz, DMSO-d_6)

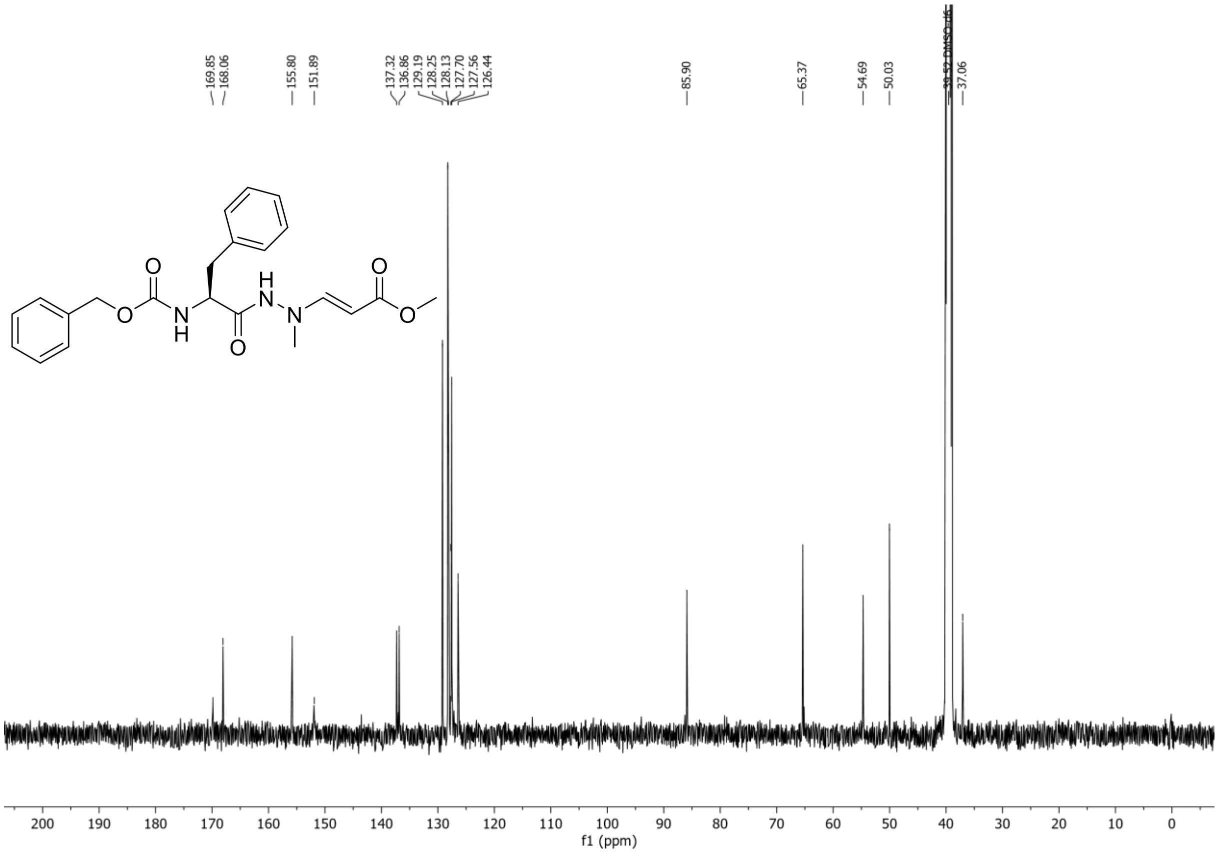

Compound 2

^{1}H NMR (600 MHz, DMSO-d_6)

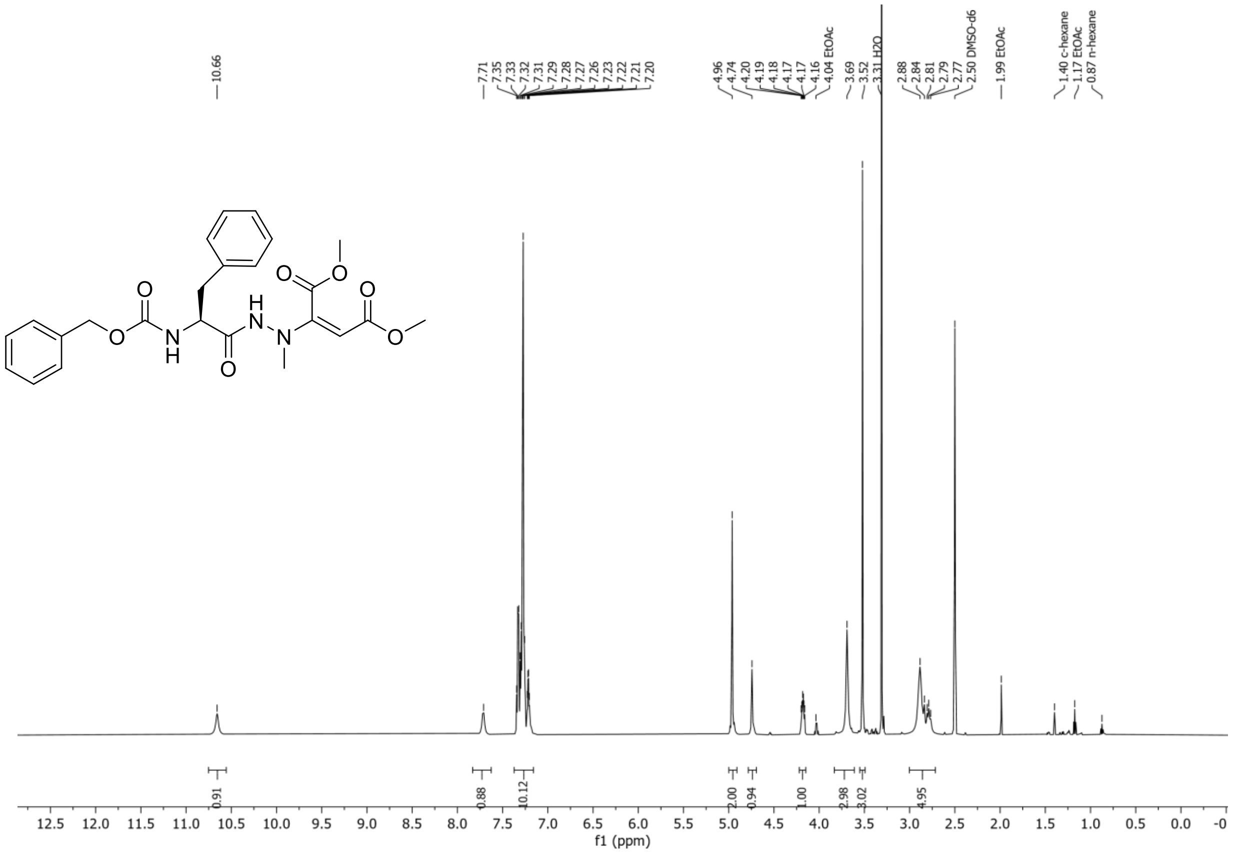

^{13}C NMR {^{1}H} (151 MHz, DMSO-d_6)

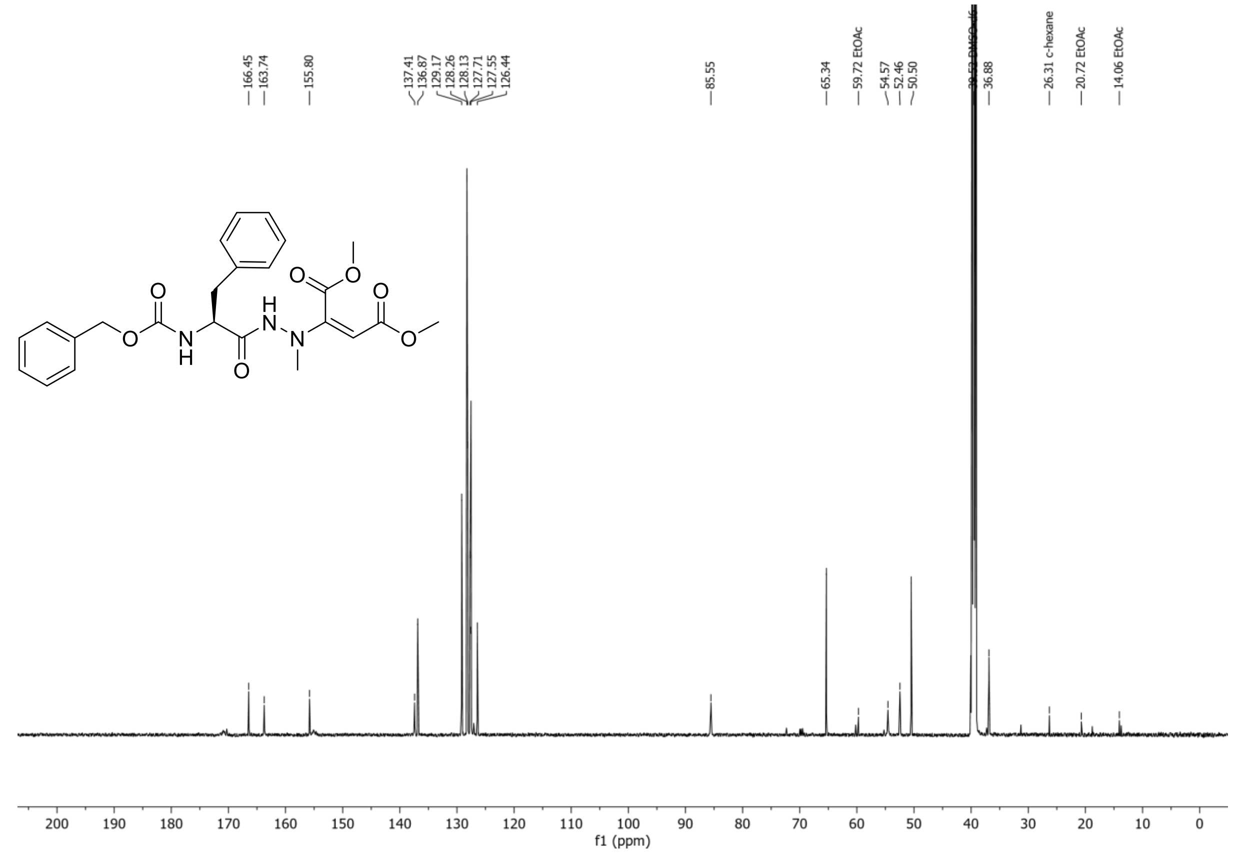

Compound 3

^{1}H NMR (500 MHz, DMSO-d_6)

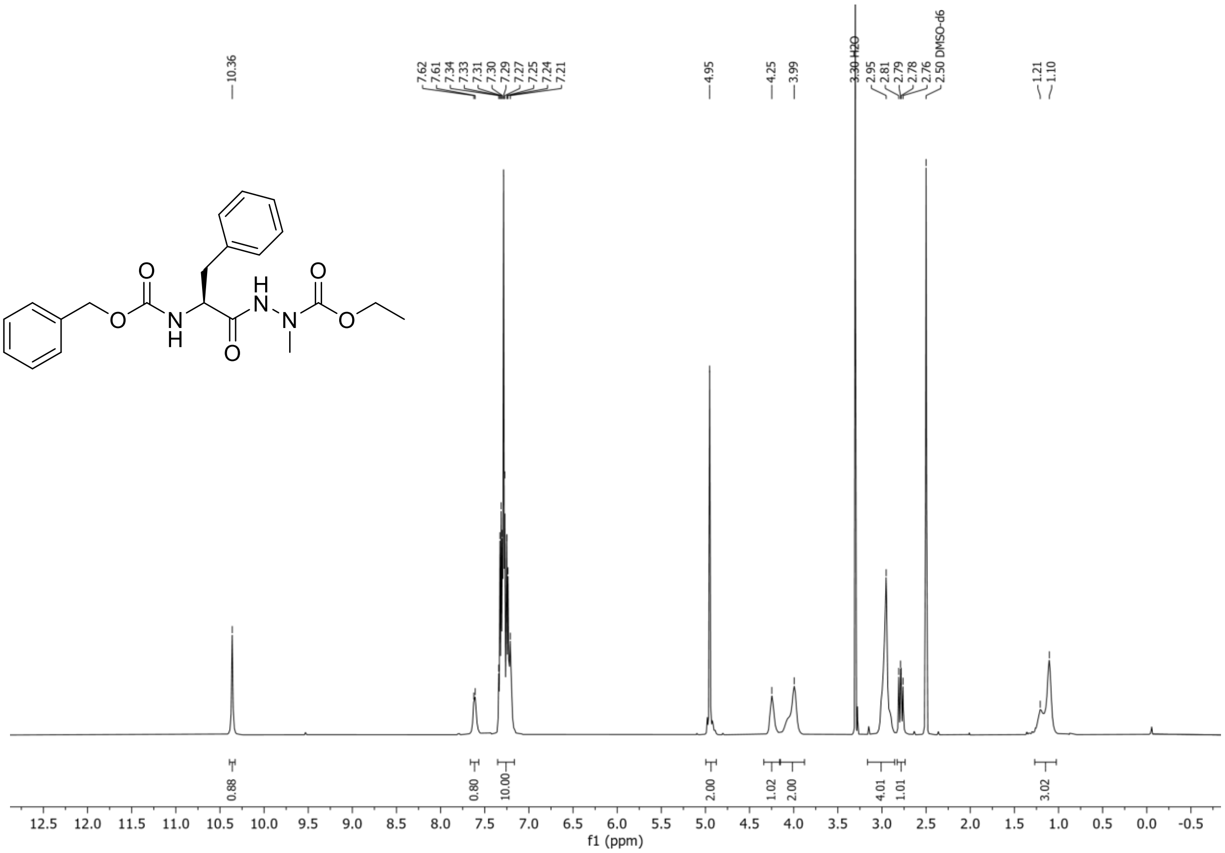

^{13}C NMR {^{1}H} (126 MHz, DMSO-d_6)

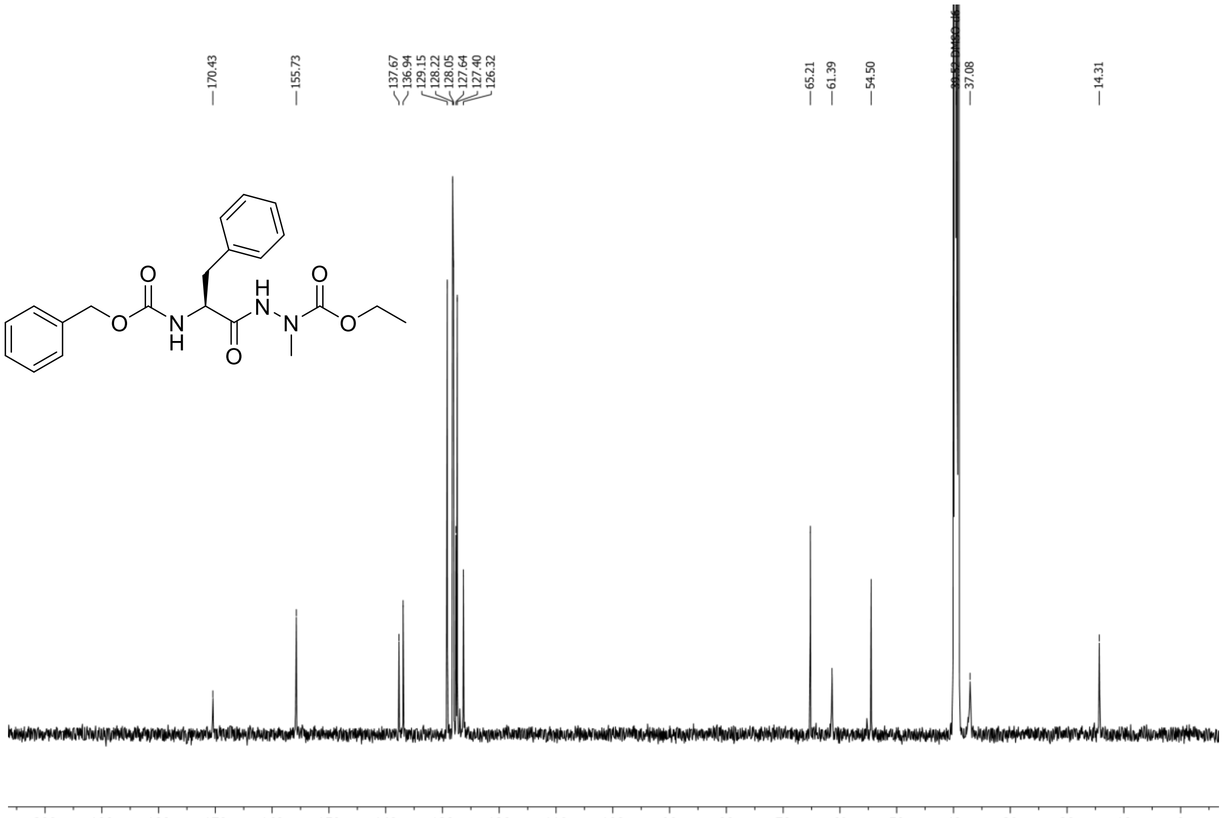

Compound 4

A: ^{1}H NMR (600 MHz, DMSO-d_6)

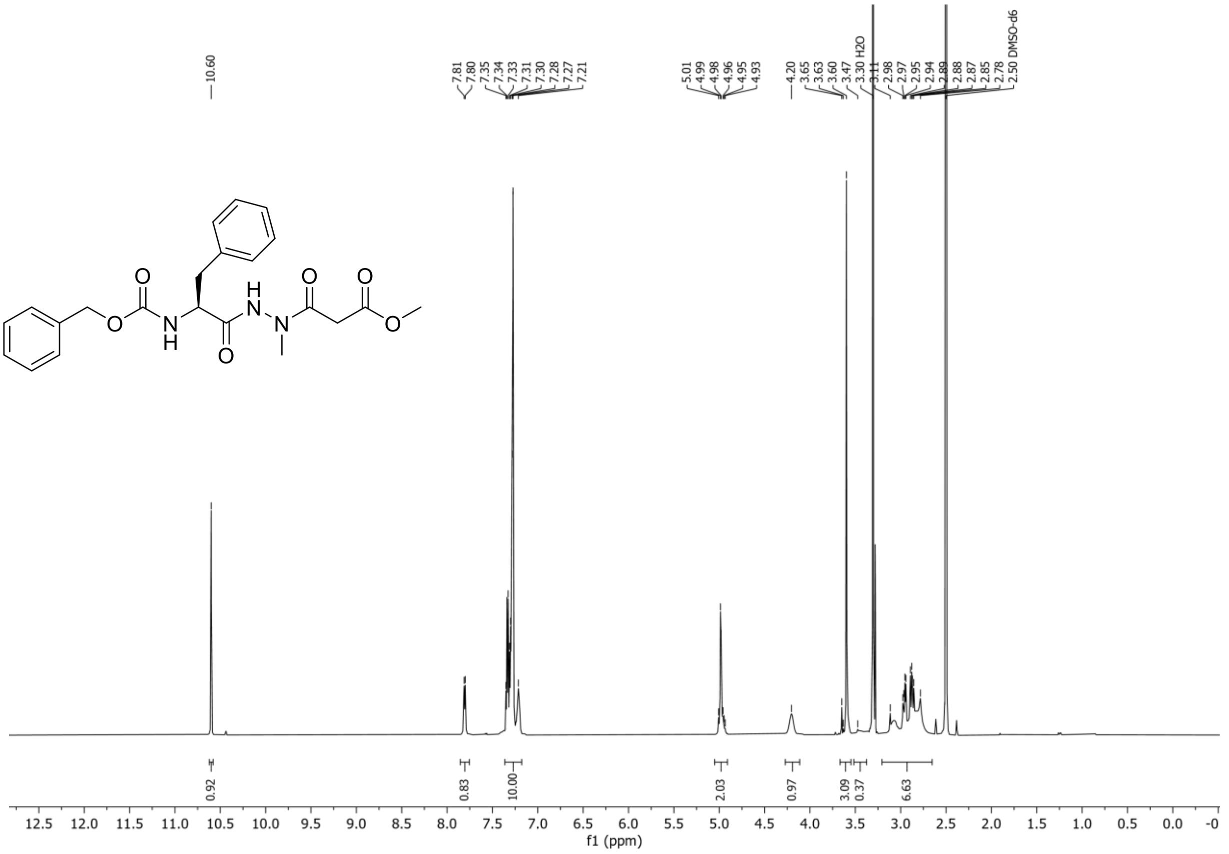

^{13}C NMR {^{1}H} (151 MHz, DMSO-d_6)

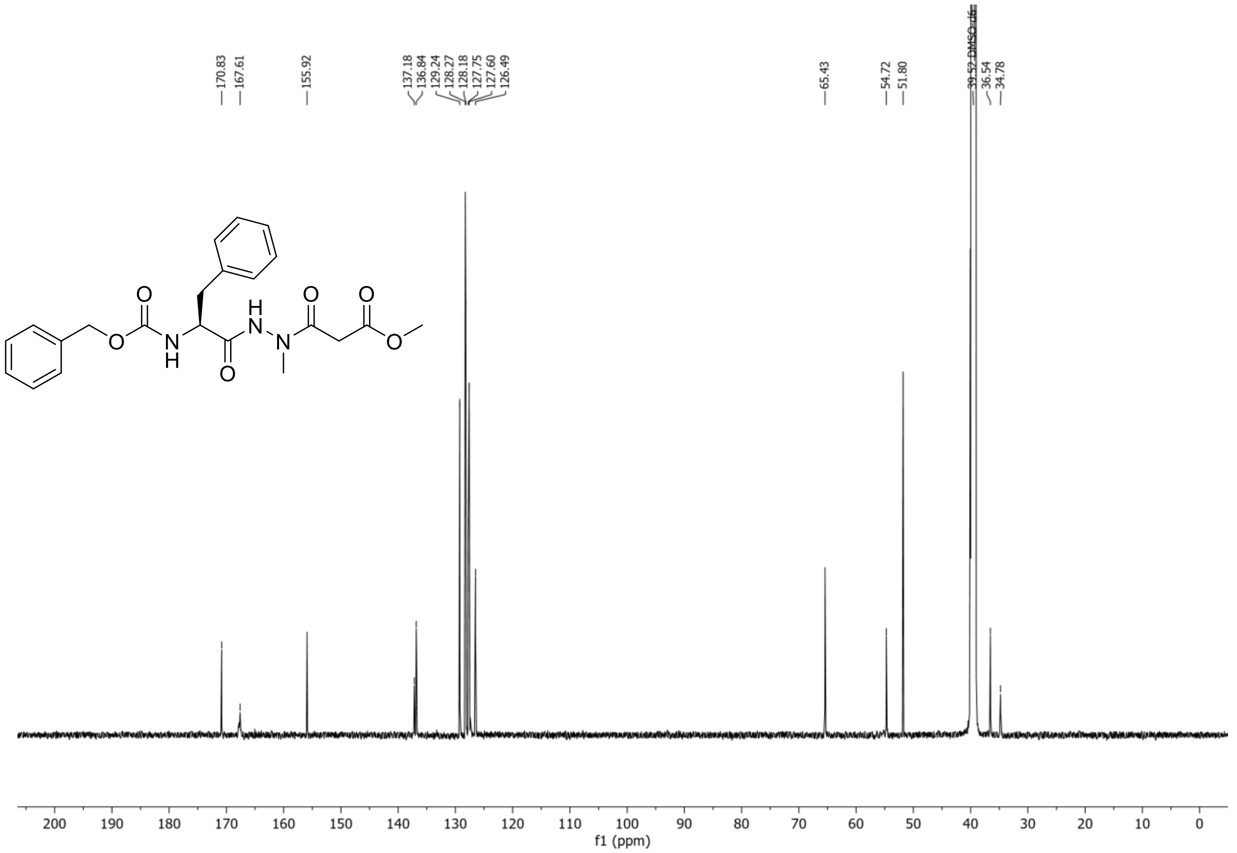

B: ^{1}H NMR (600 MHz, DMSO-d_6)

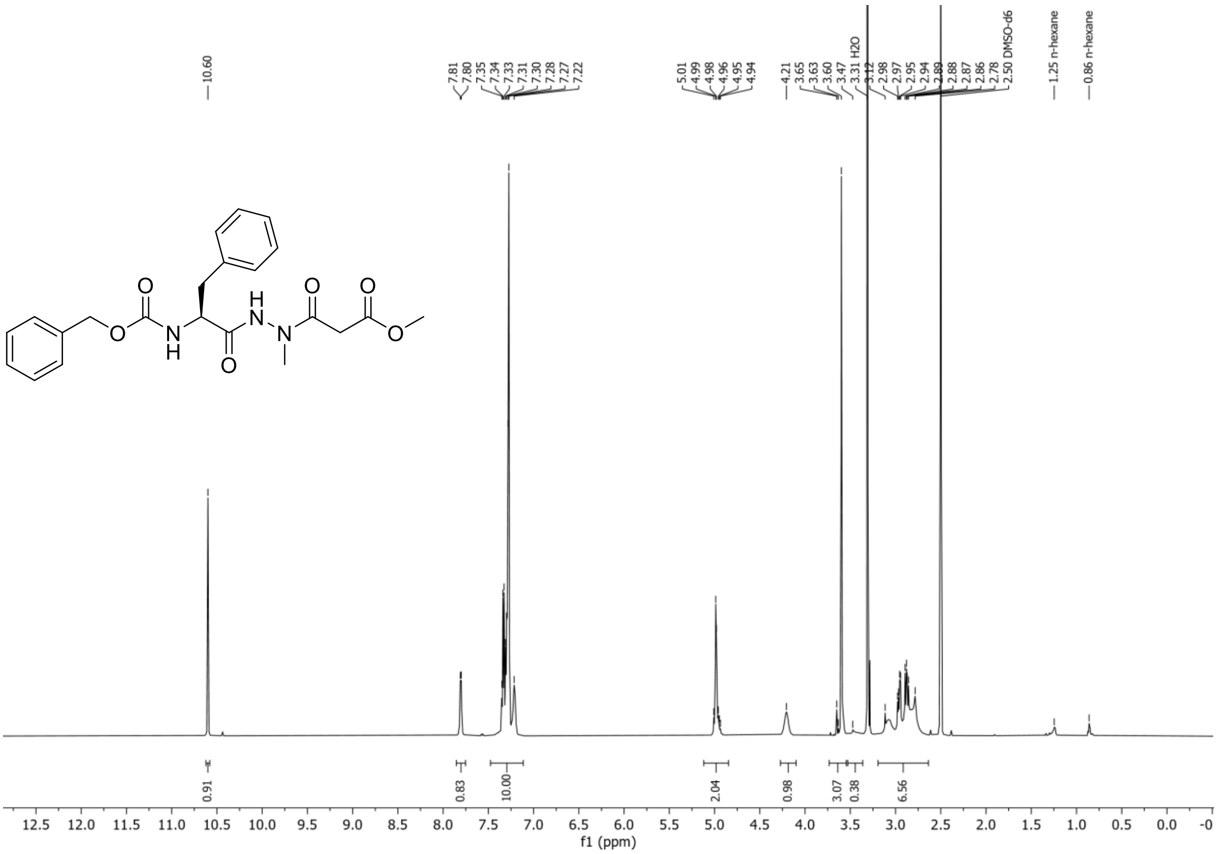

^{13}NMR {^{1}H} (151 MHz, DMSO-d_6)

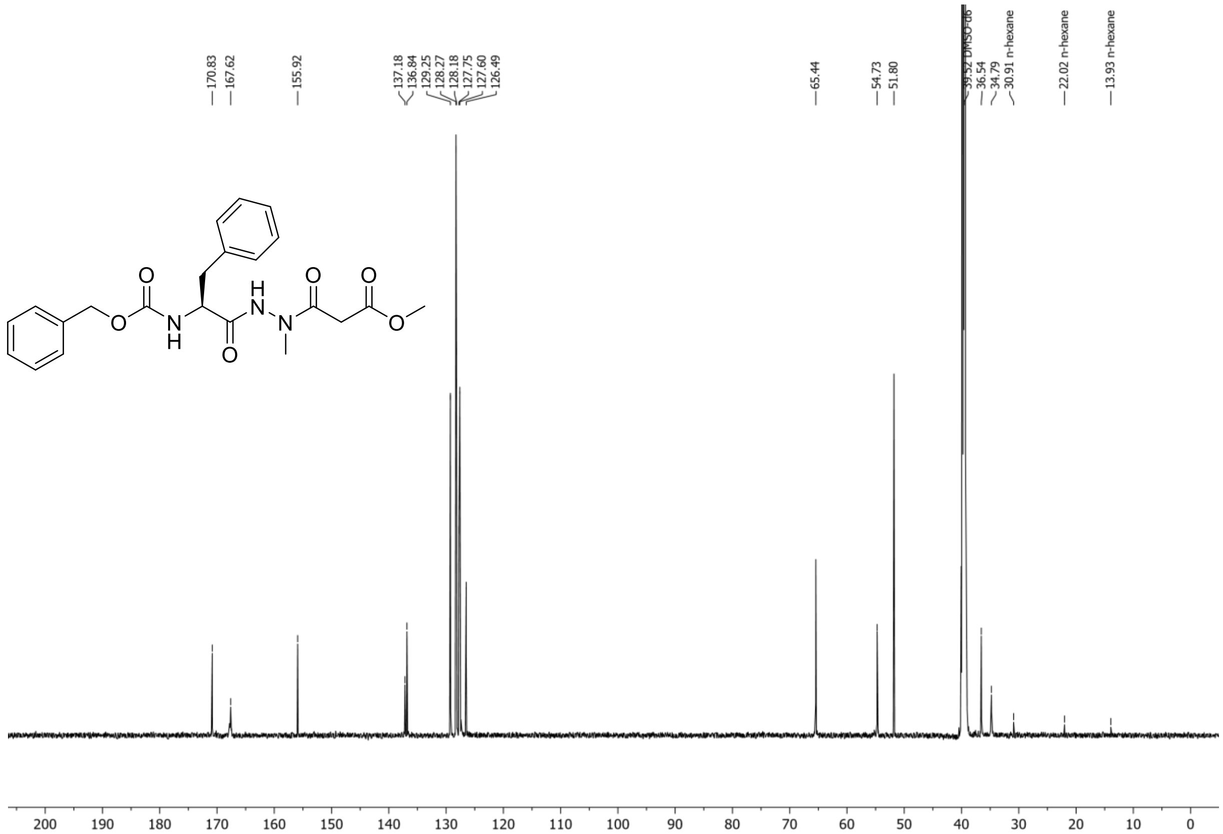

Compound 5

^{1}H NMR (500 MHz, DMSO-d_6)

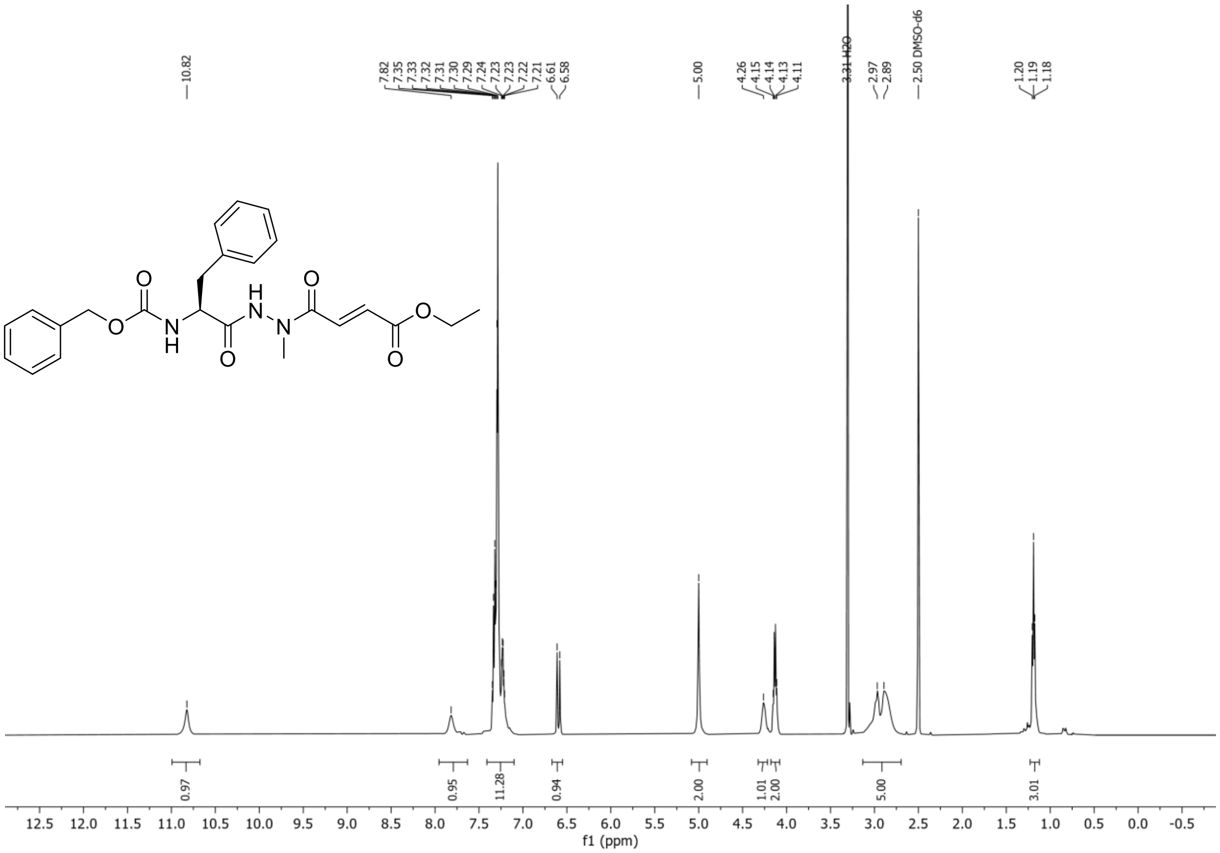

^{13}C NMR (126 MHz, DMSO-d_6)

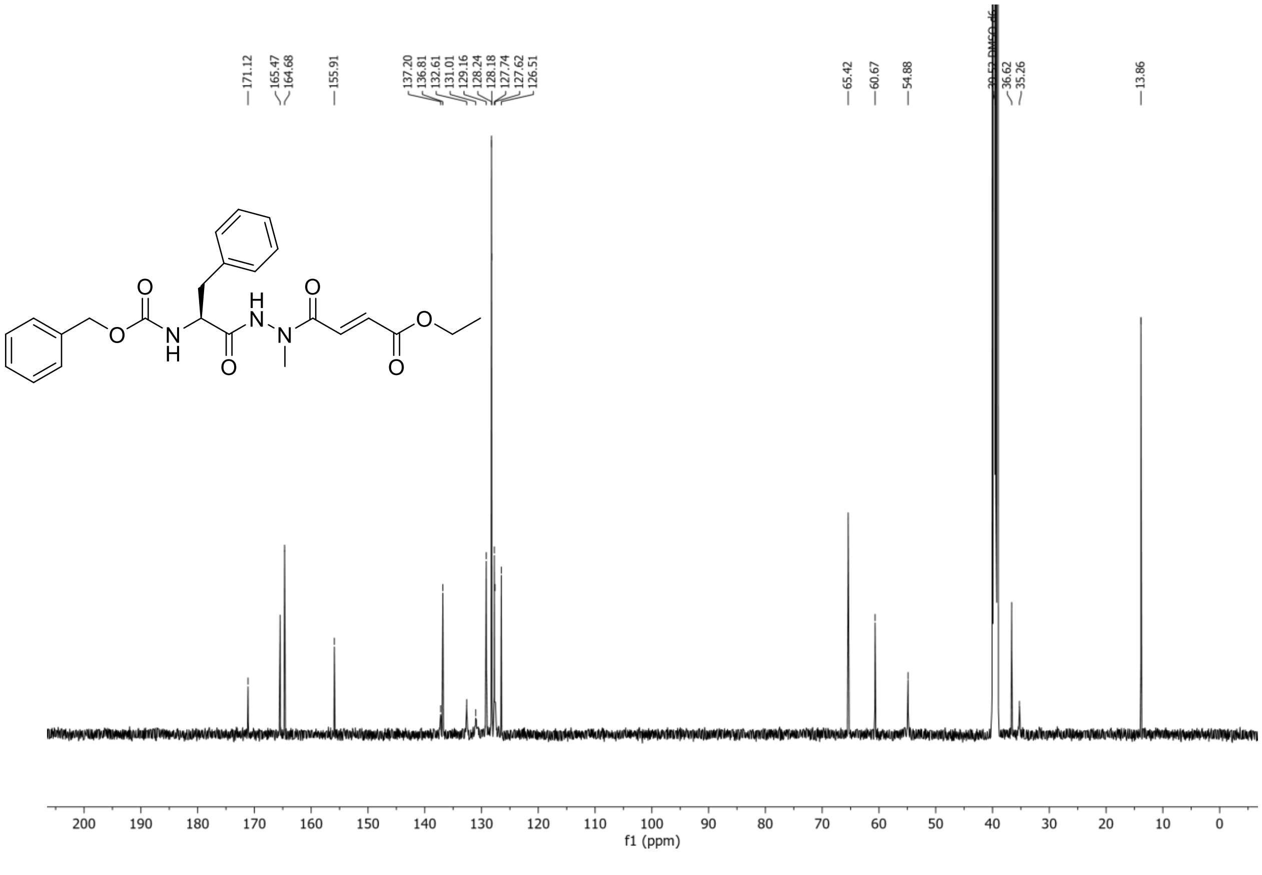

Compound 6

^{1}H NMR (600 MHz, DMSO-d_6)

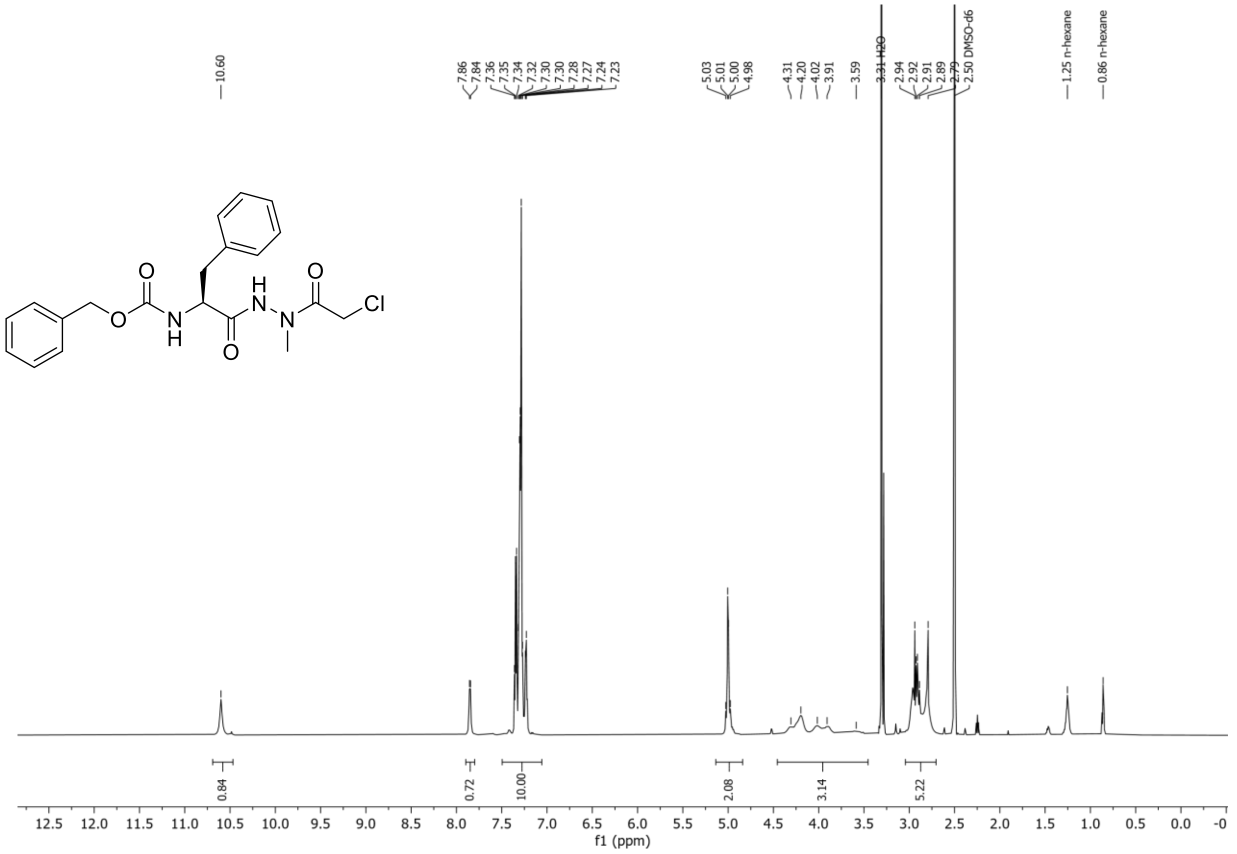

^{13}C NMR {^{1}H} (151 MHz, DMSO-d_6)

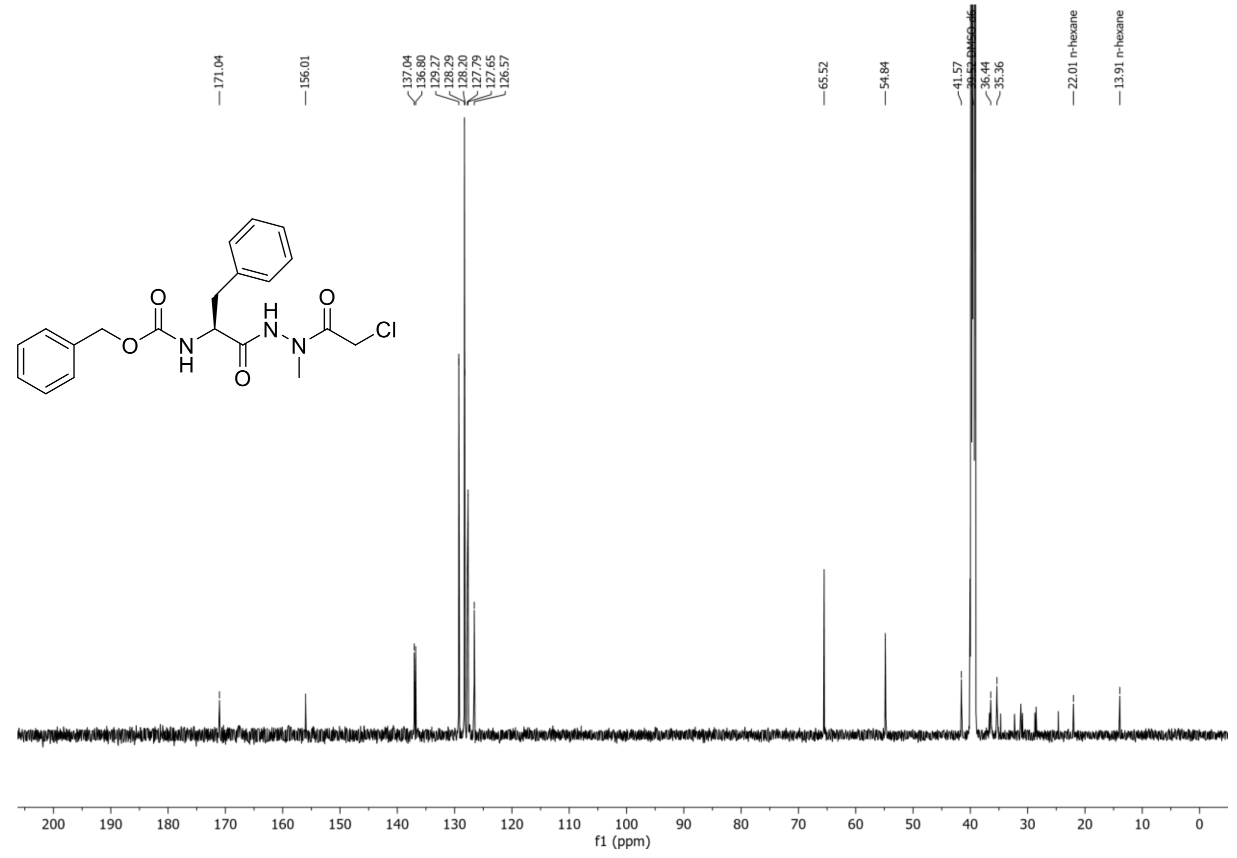

Compound 7

^{1}H NMR (600 MHz, DMSO-d_6)

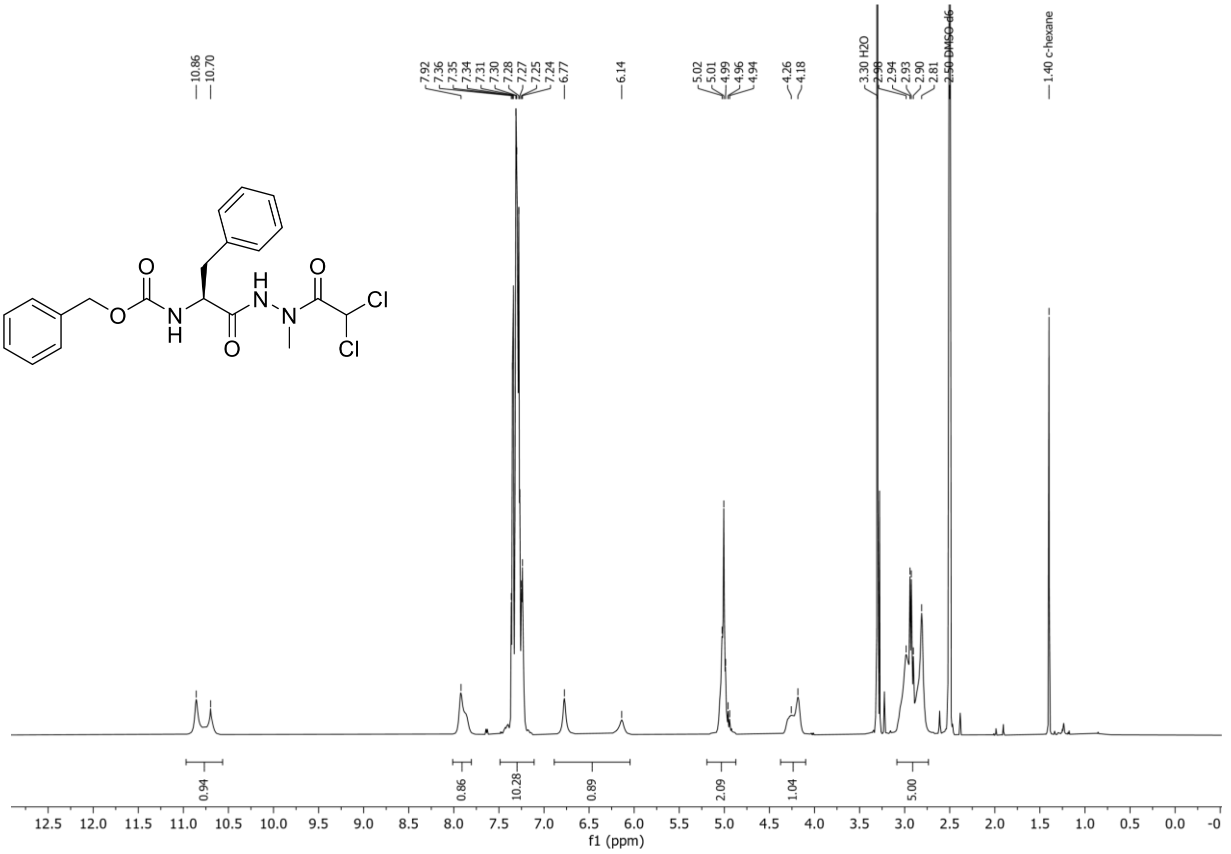

^{13}C NMR {^{1}H} (151 MHz, DMSO-d_6)

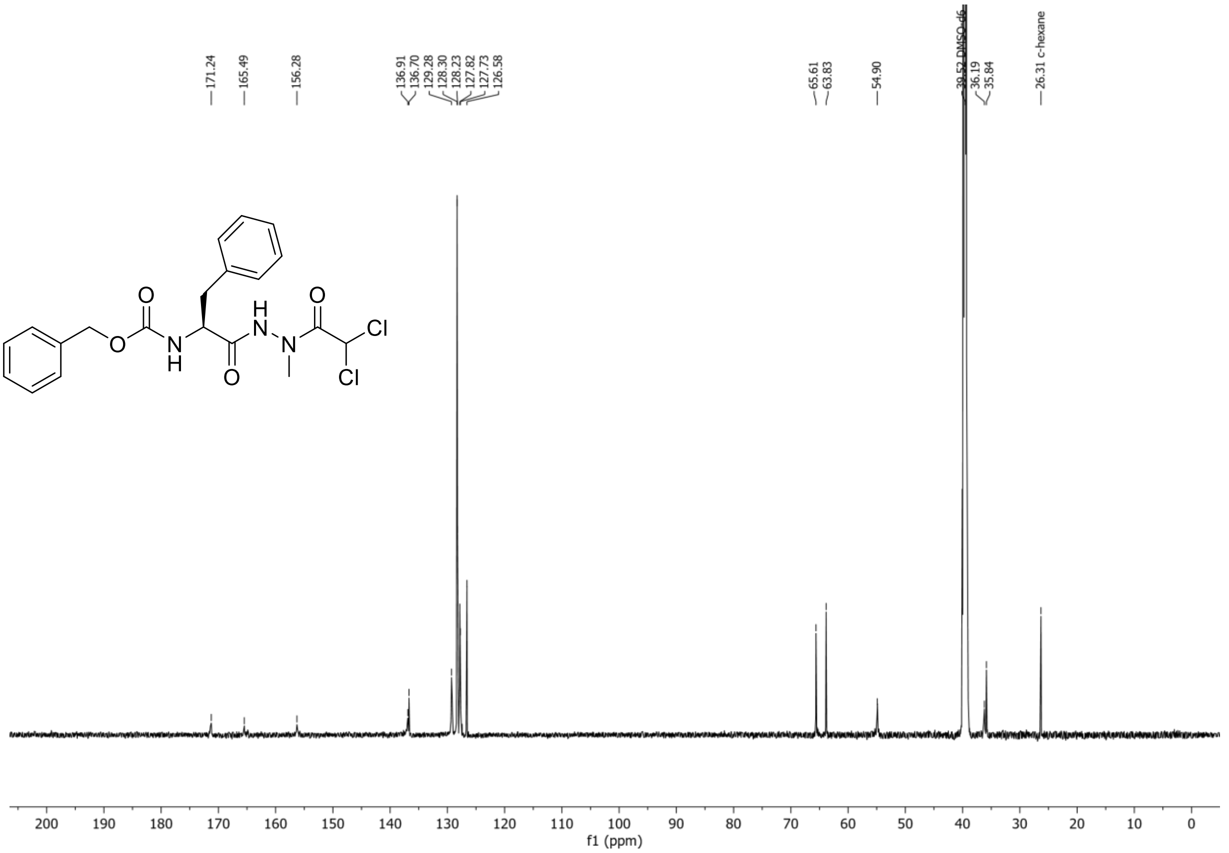

Compound 8

^{1}H NMR (500 MHz, DMSO-d_6)

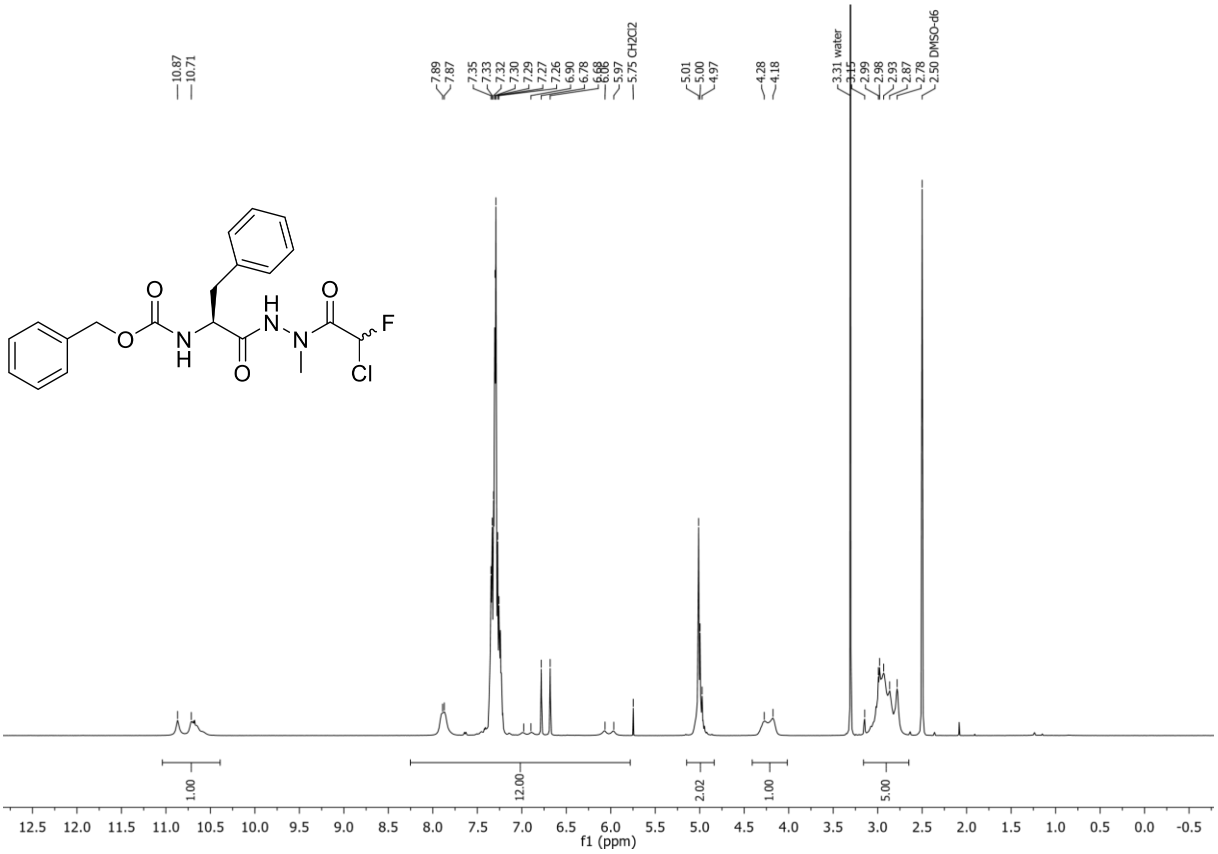

^{13}C NMR {^{1}H} (126 MHz, DMSO-d_6)

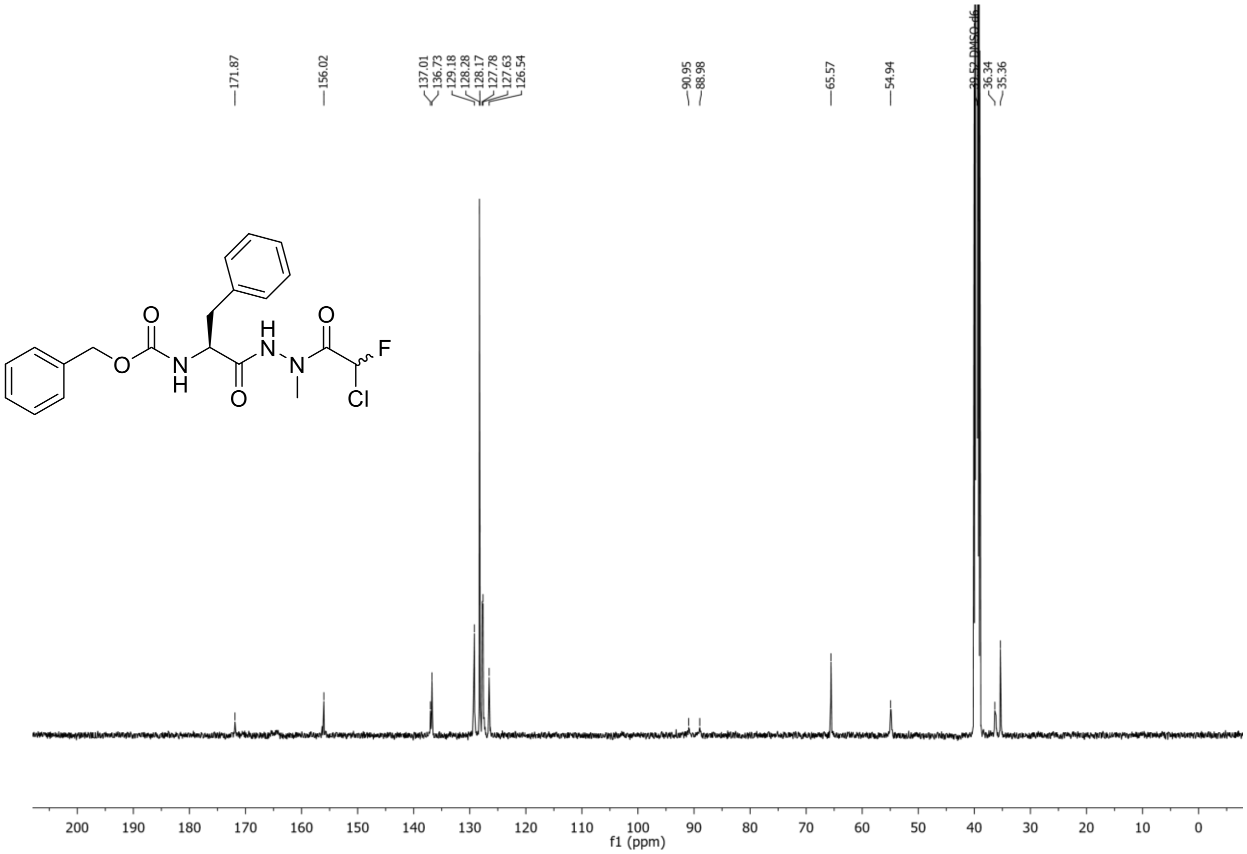

^{19}F NMR (471 MHz, DMSO-d_6)

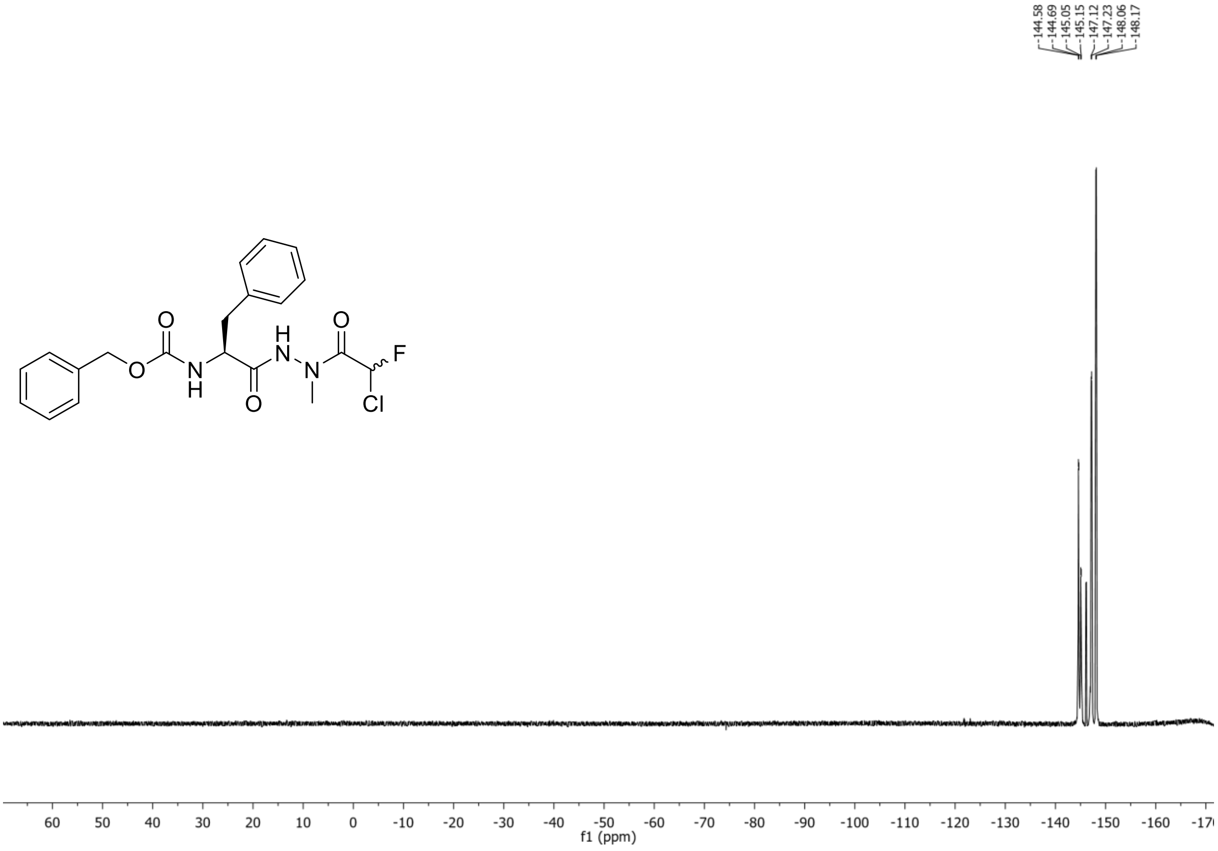

Compound 9

^{1}H NMR (500 MHz, DMSO-d_6)

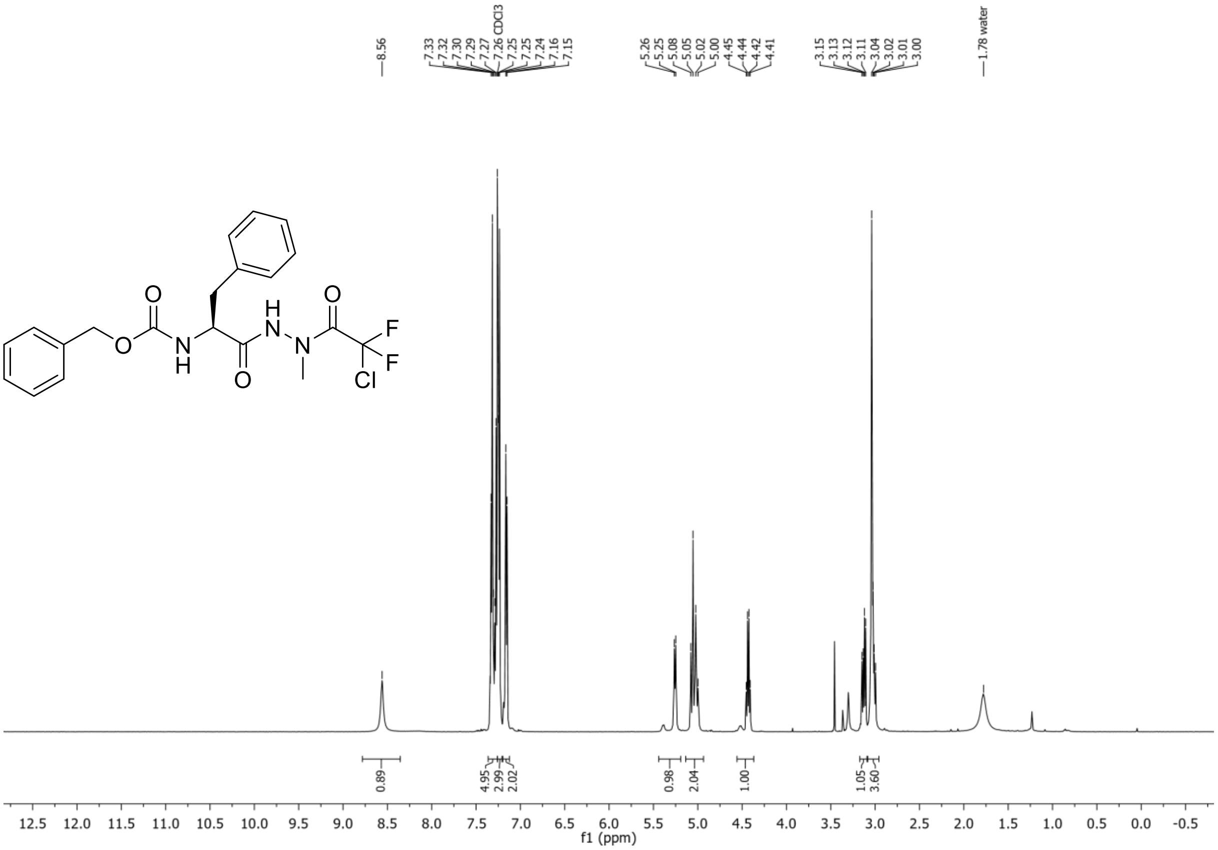

^{13}C NMR (126 MHz, DMSO-d_6)

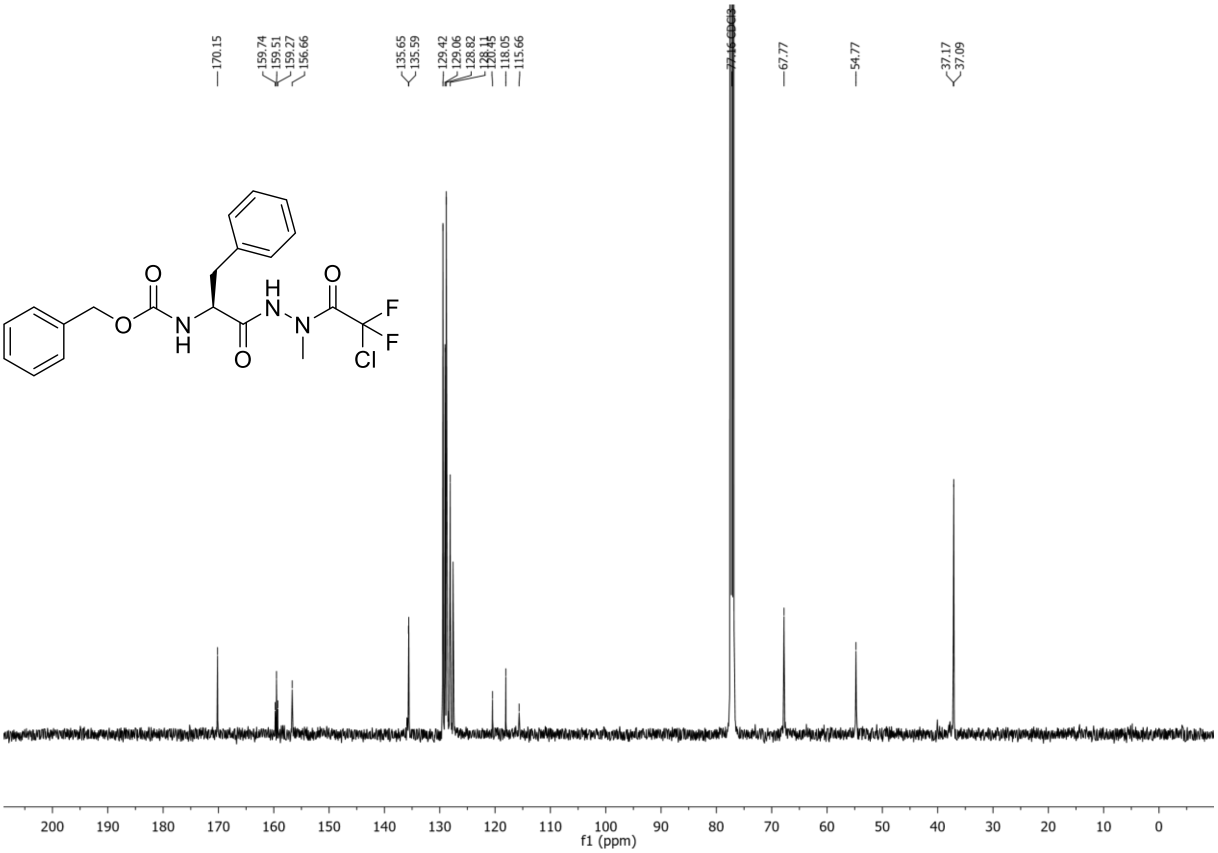

^{19}F NMR (471 MHz, DMSO-d_6)

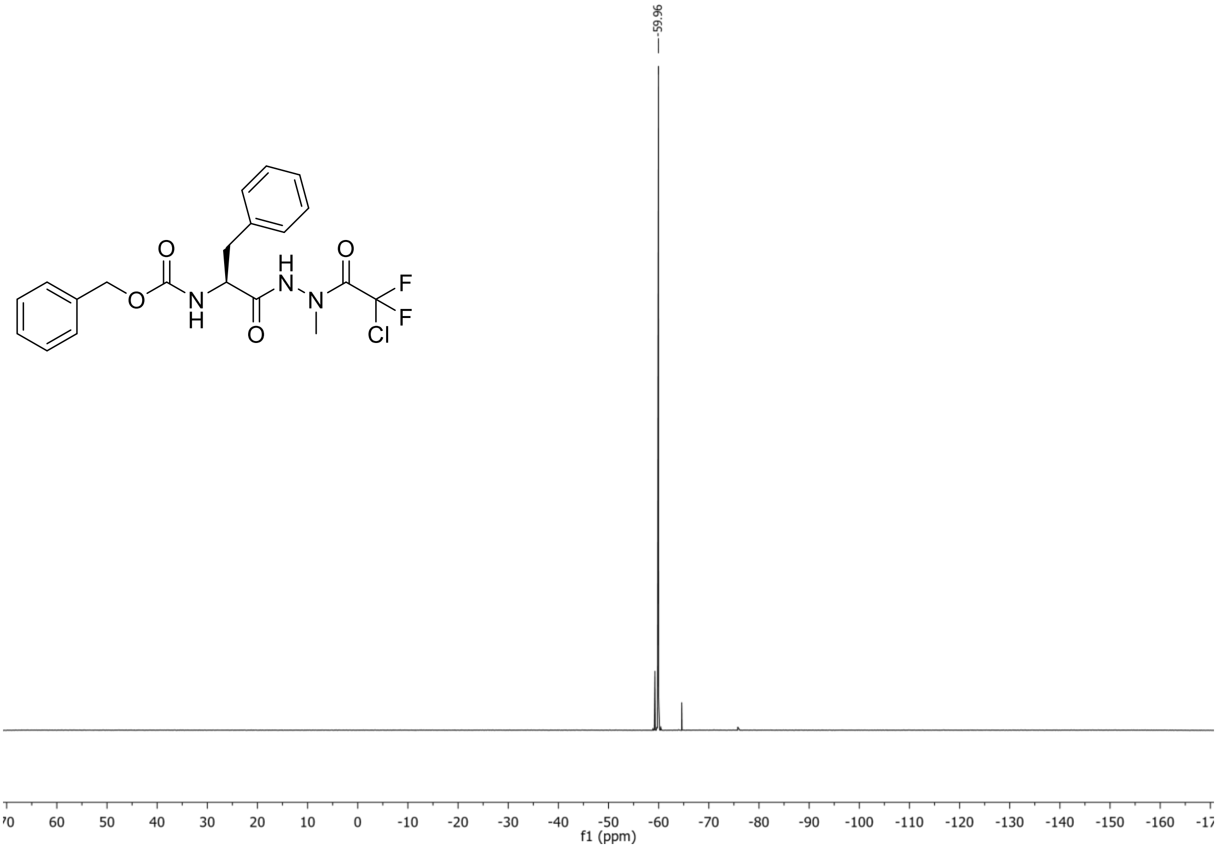

Compound 10

^{1}H NMR (500 MHz, DMSO-d_6)

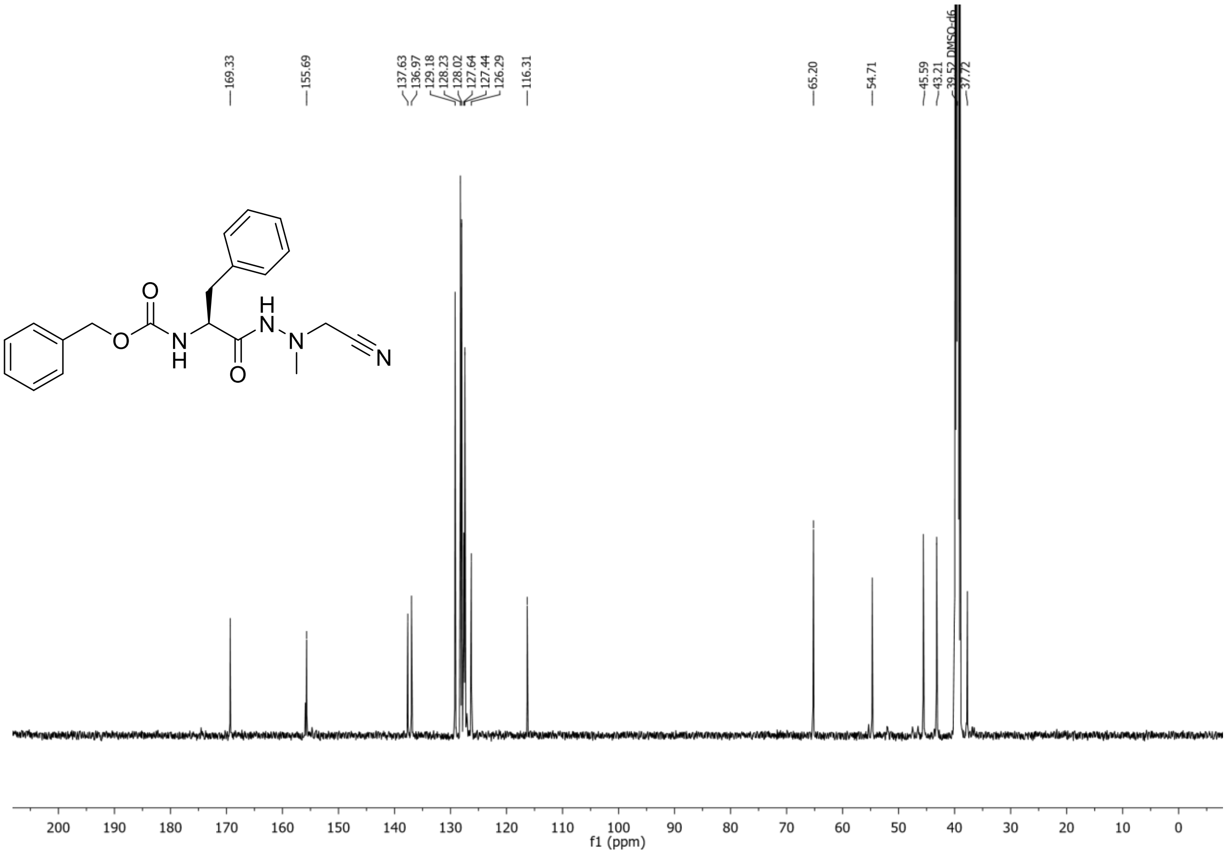

^{13}C NMR (126 MHz, DMSO-d_6)

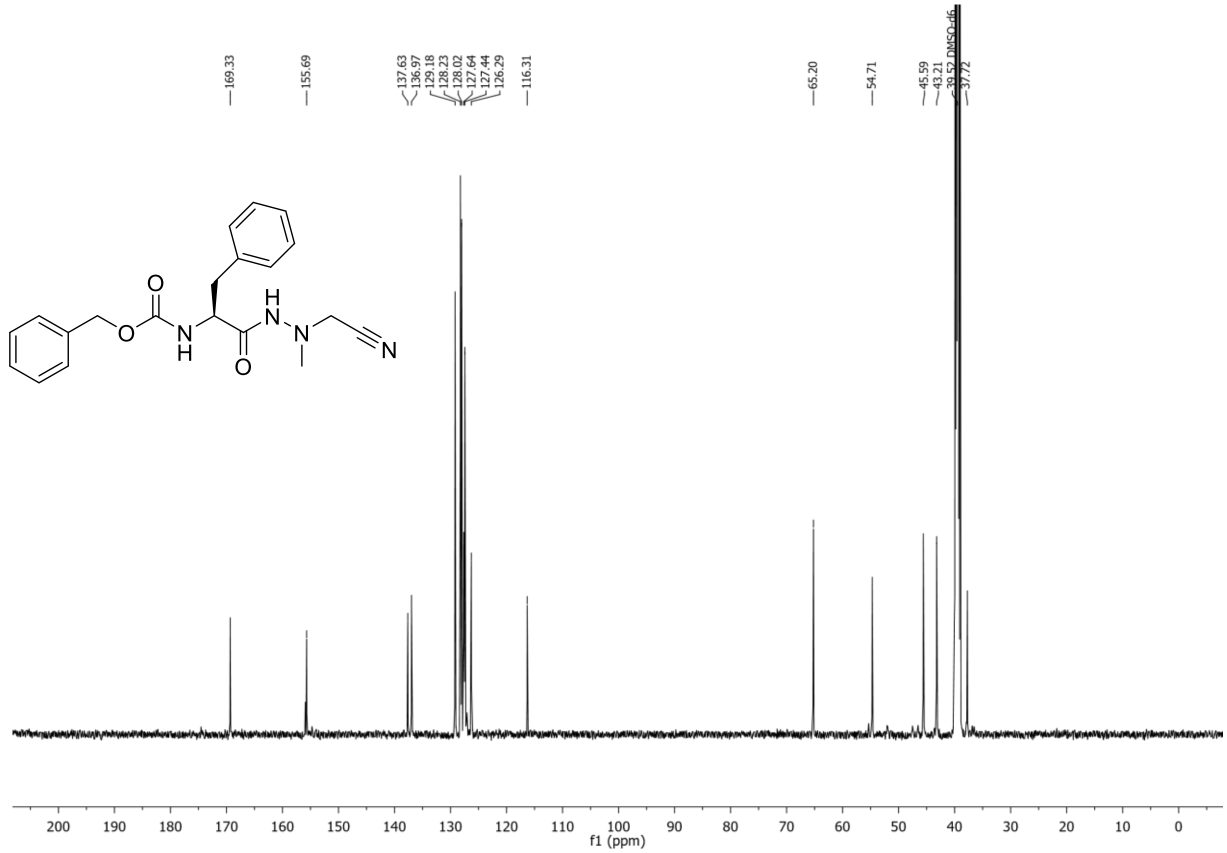

Compound 11

^{1}H NMR (600 MHz, DMSO-d_6)

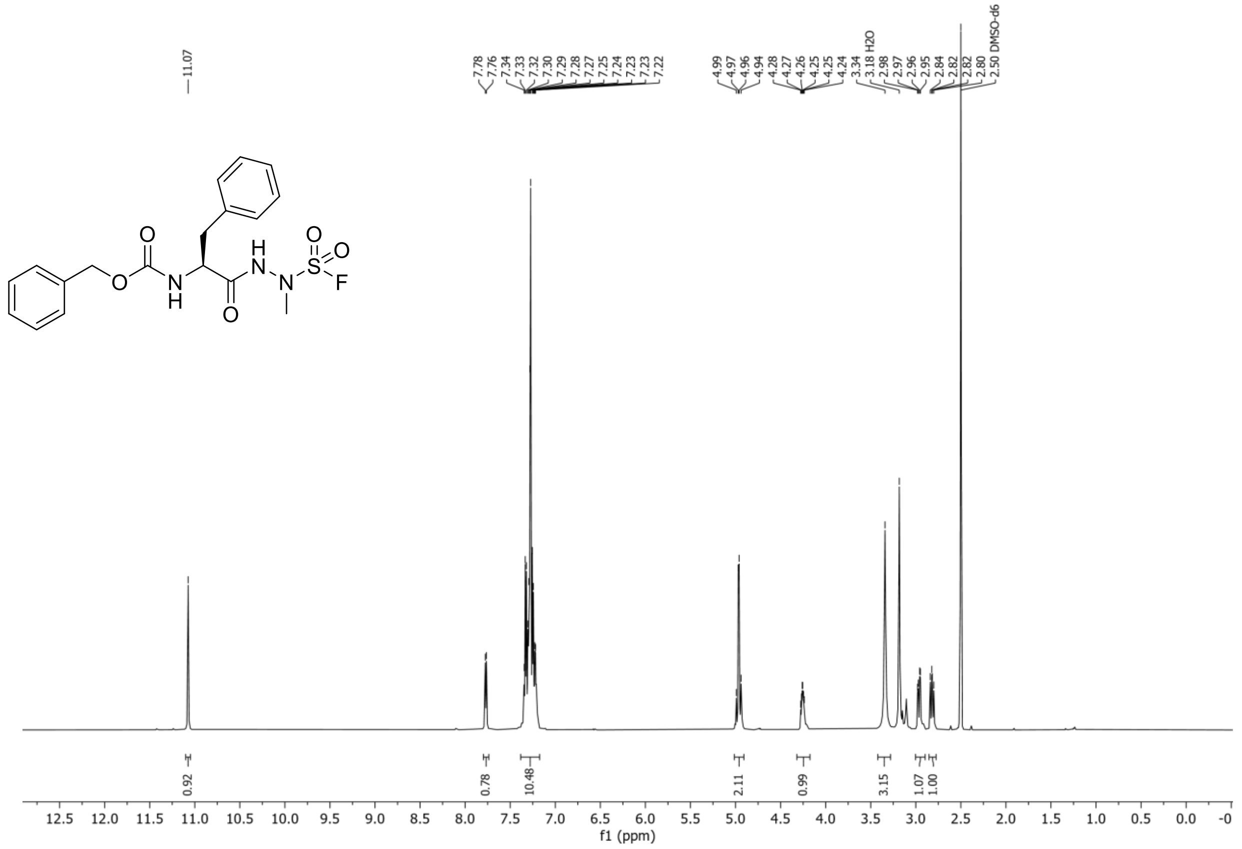

^{13}C NMR {^{1}H} (151 MHz, DMSO-d_6)

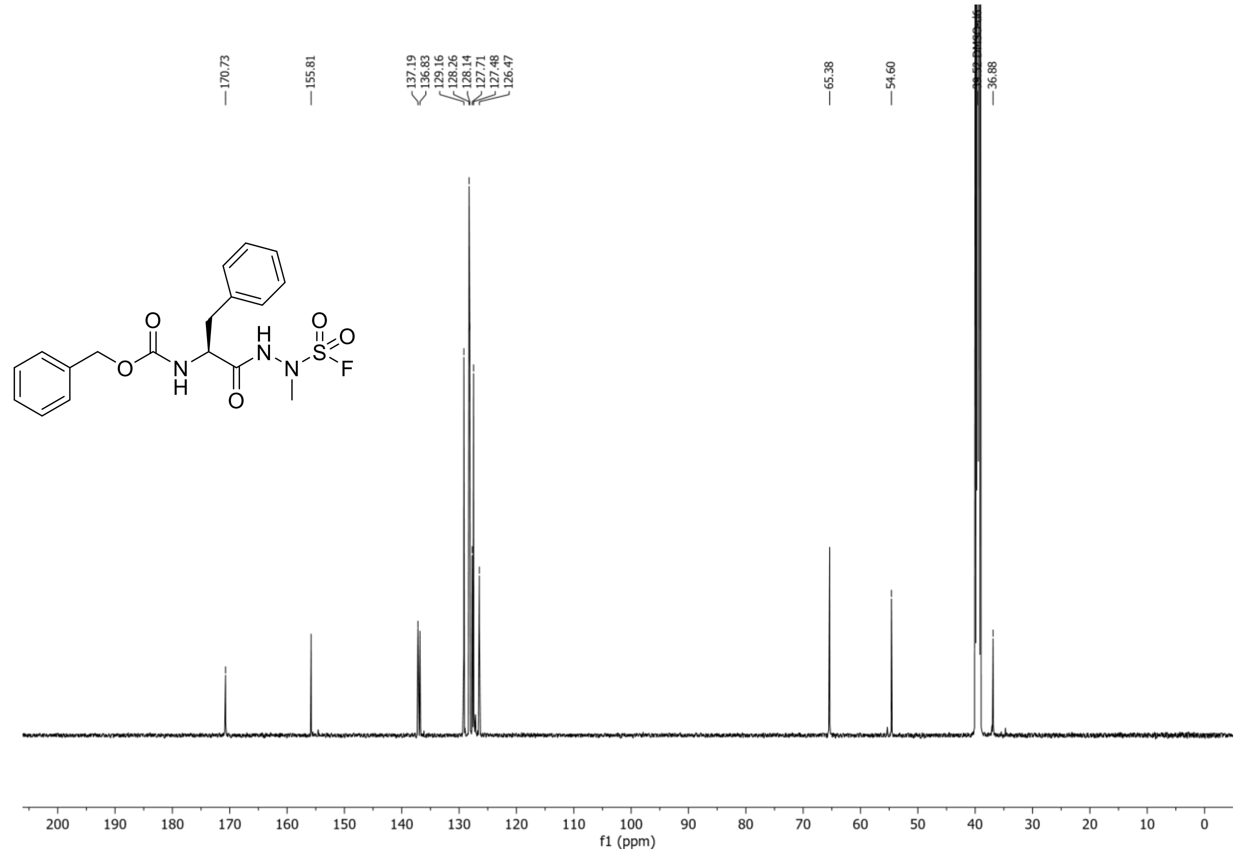

^{19}F NMR (565 MHz, DMSO-d_6)

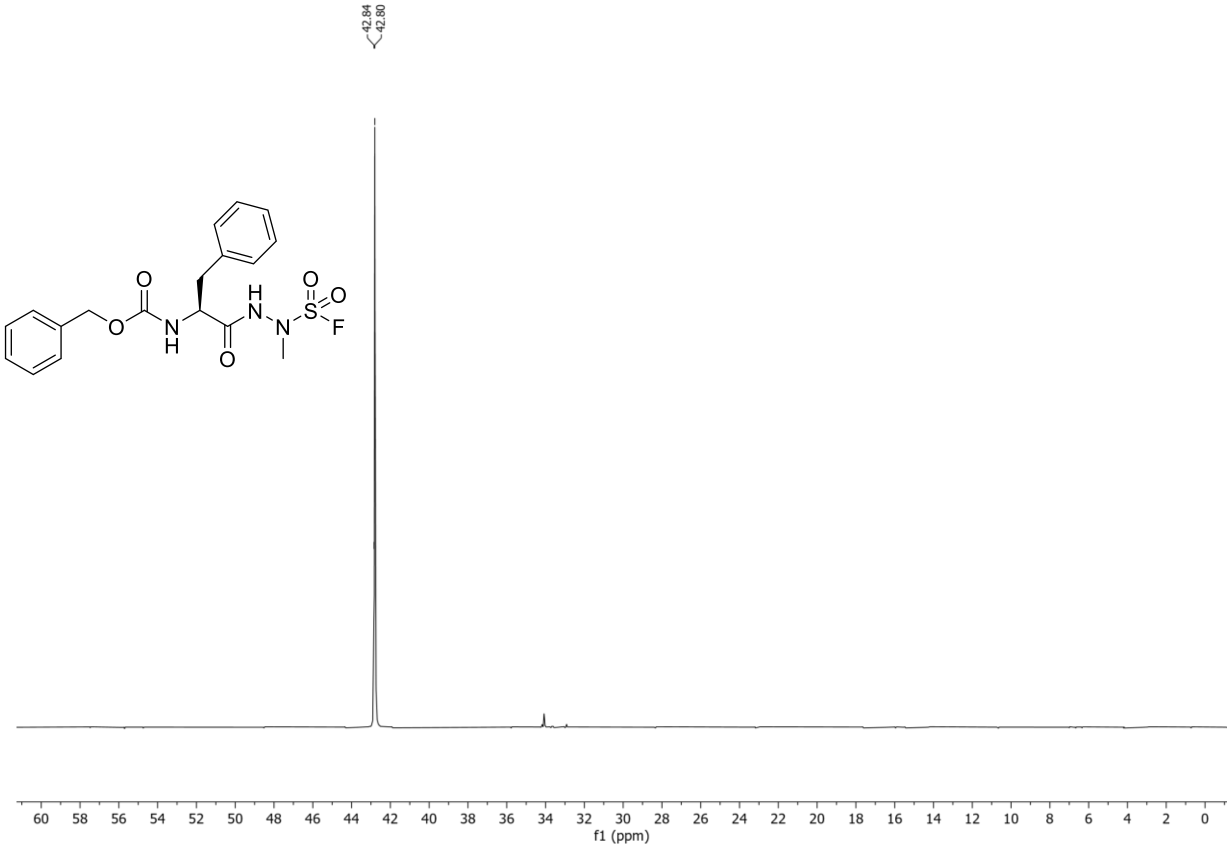

Compound 12

^{1}H NMR (500 MHz, DMSO-d_6)

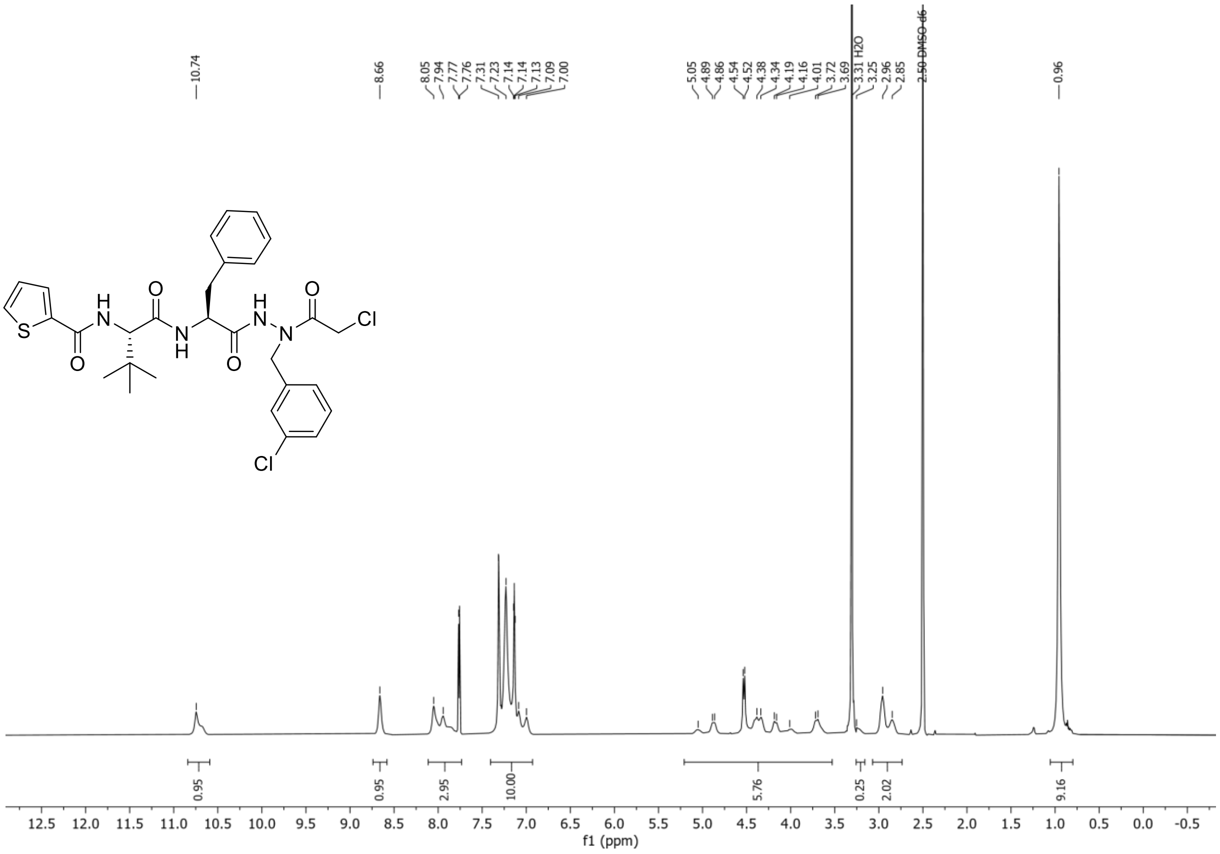

^{13}C NMR {^{1}H} (126 MHz, DMSO-d_6)

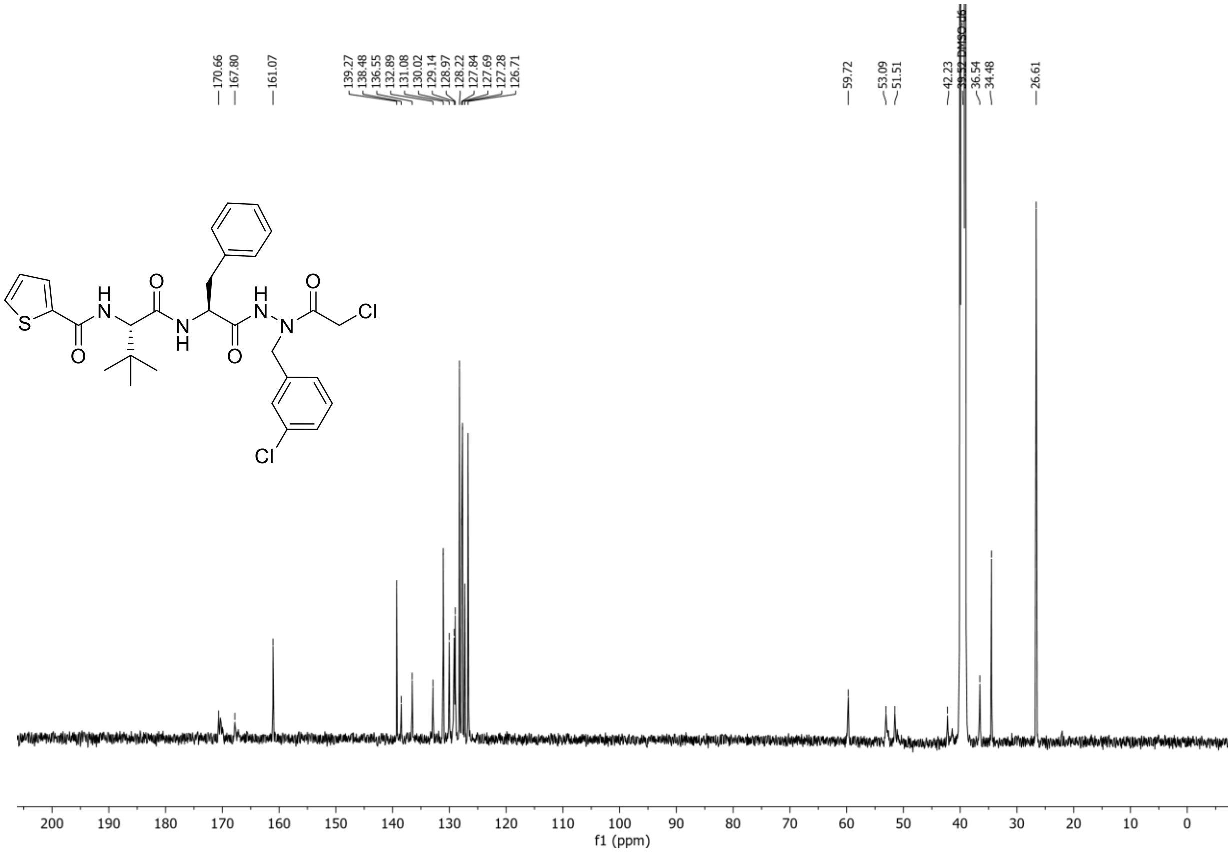

Compound 13

^{1}H NMR (500 MHz, DMSO-d_6)

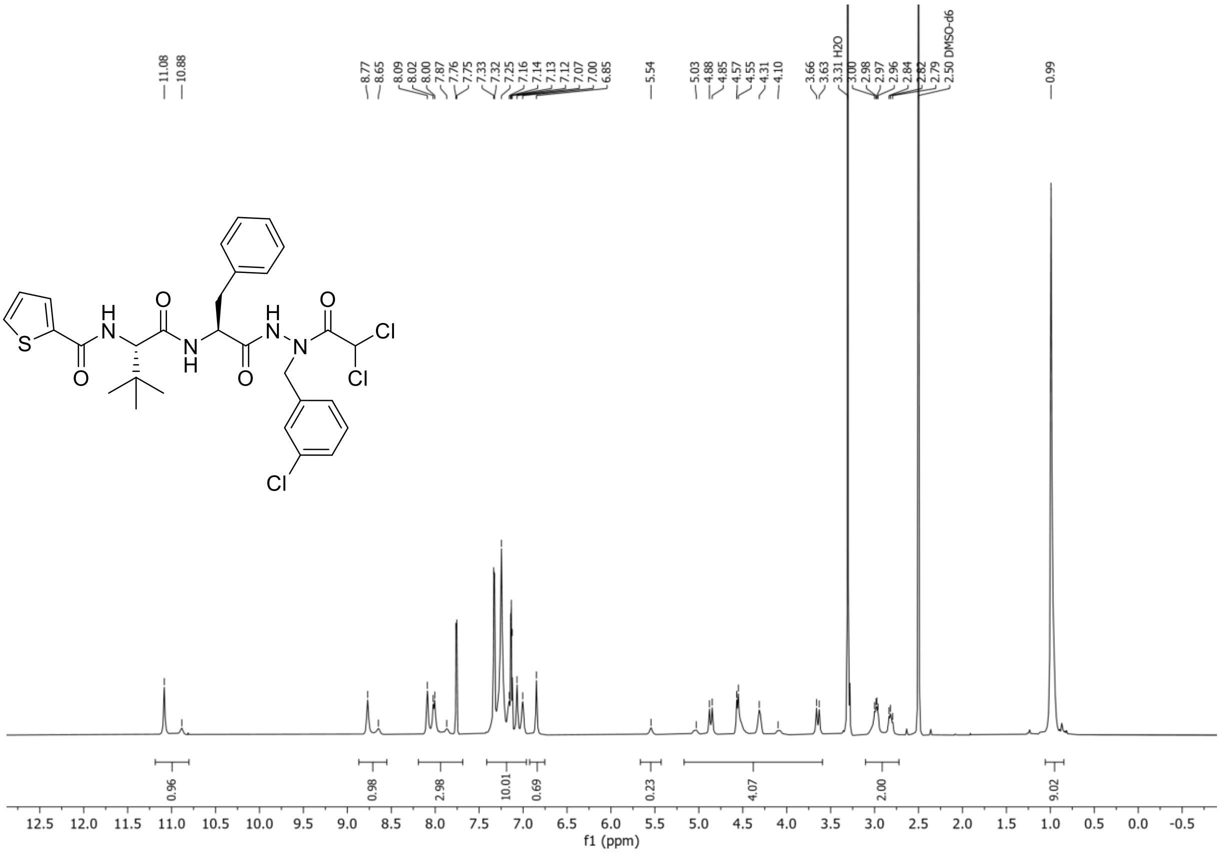

^{13}C NMR {^{1}H} (126 MHz, DMSO-d_6)

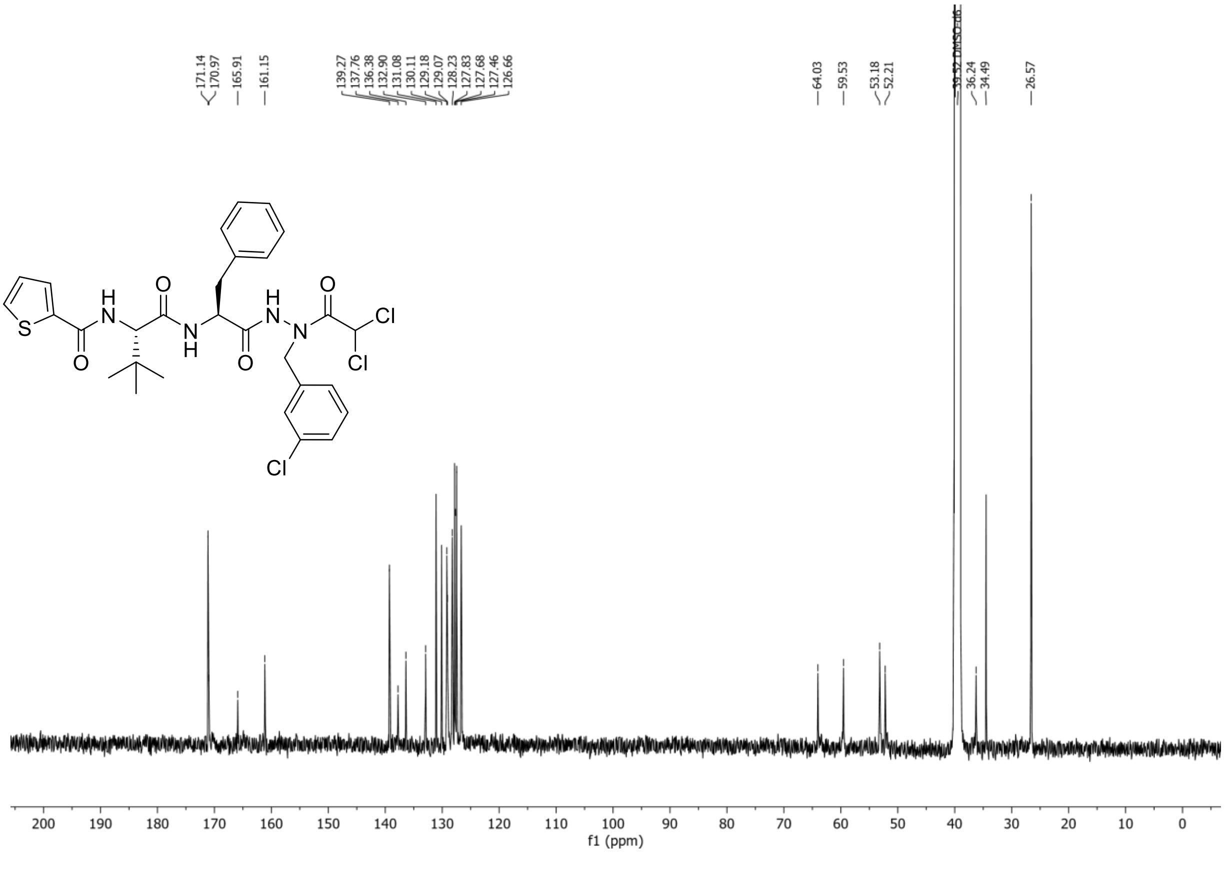

Compound 14

^{1}H NMR (600 MHz, DMSO-d_6)

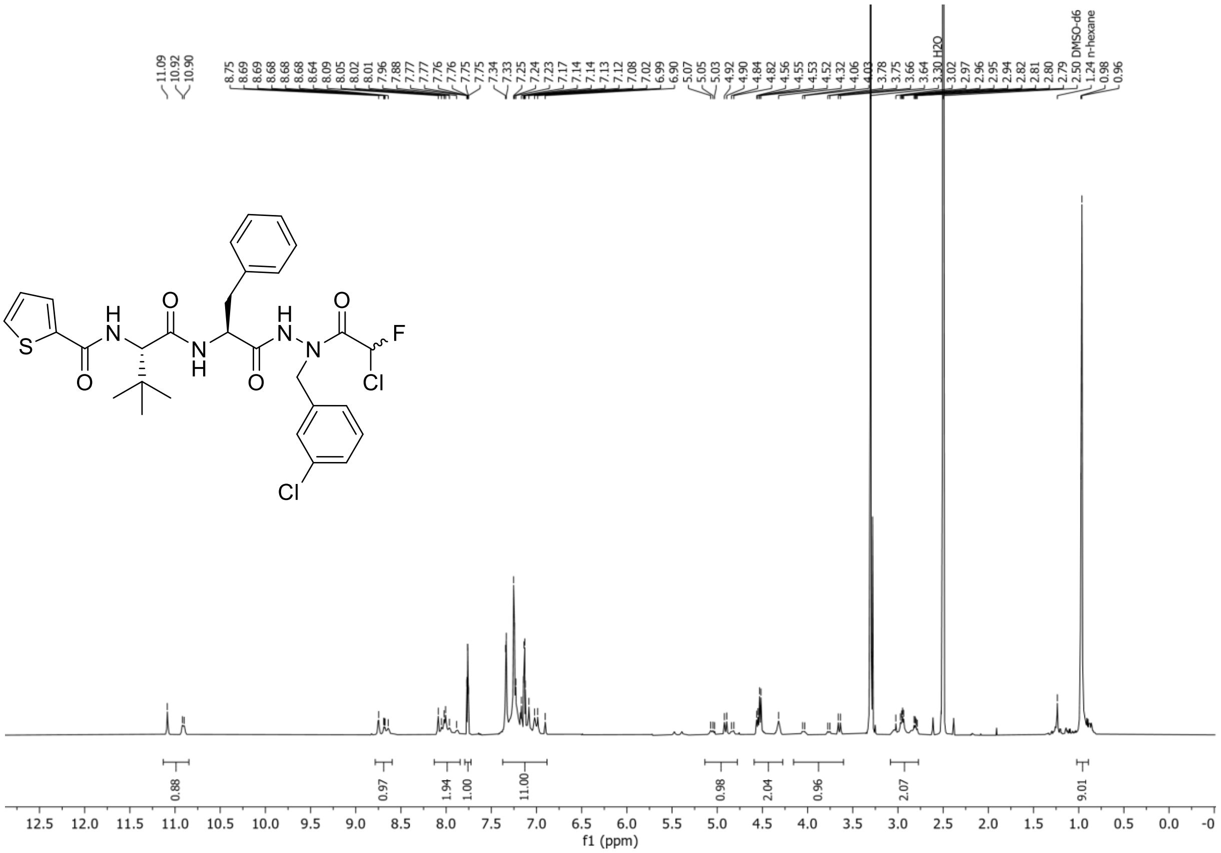

^{13}C NMR {^{1}H} (151 MHz, DMSO-d_6)

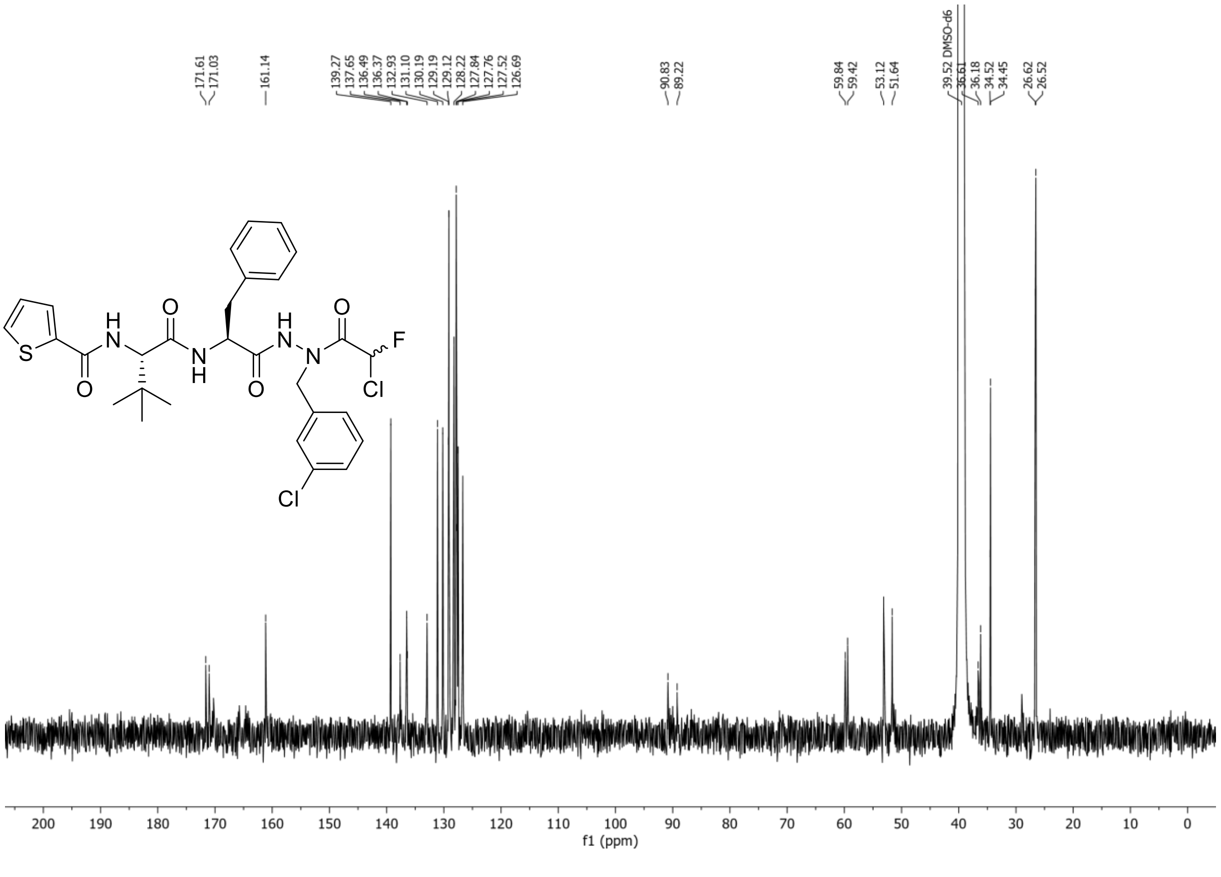

Compound 15

^{1}H NMR (500 MHz, DMSO-d_6)

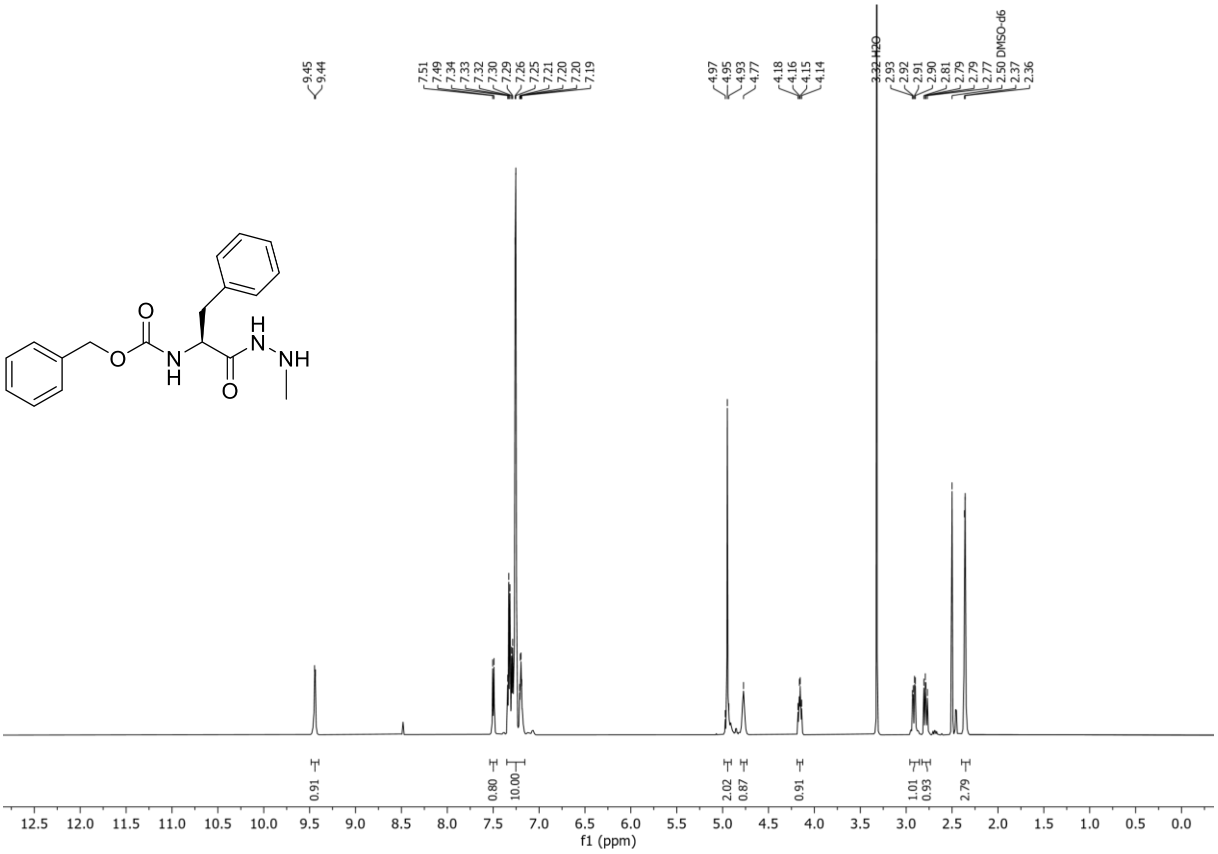

^{13}C NMR {^{1}H} (151 MHz, DMSO-d_6)

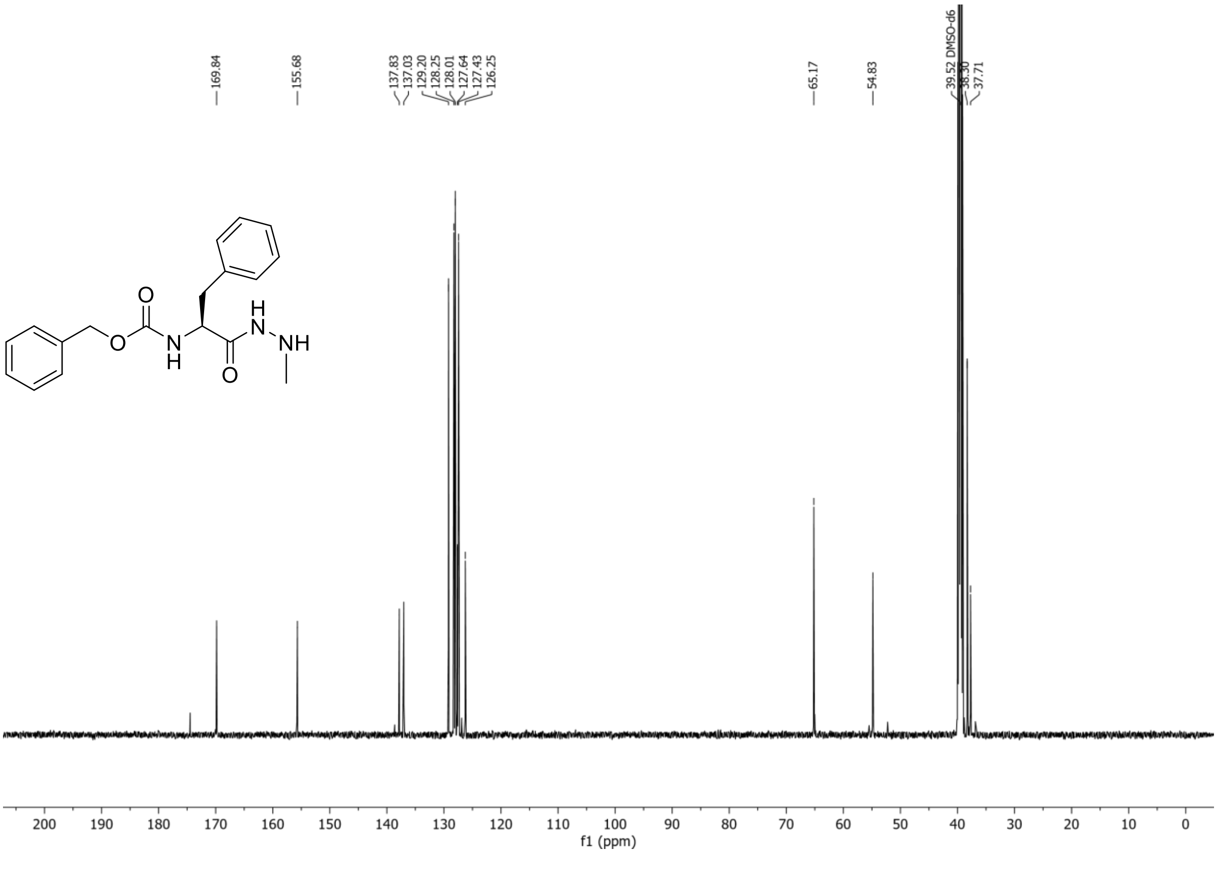

Compound 16

^{1}H NMR (500 MHz, DMSO-d_6)

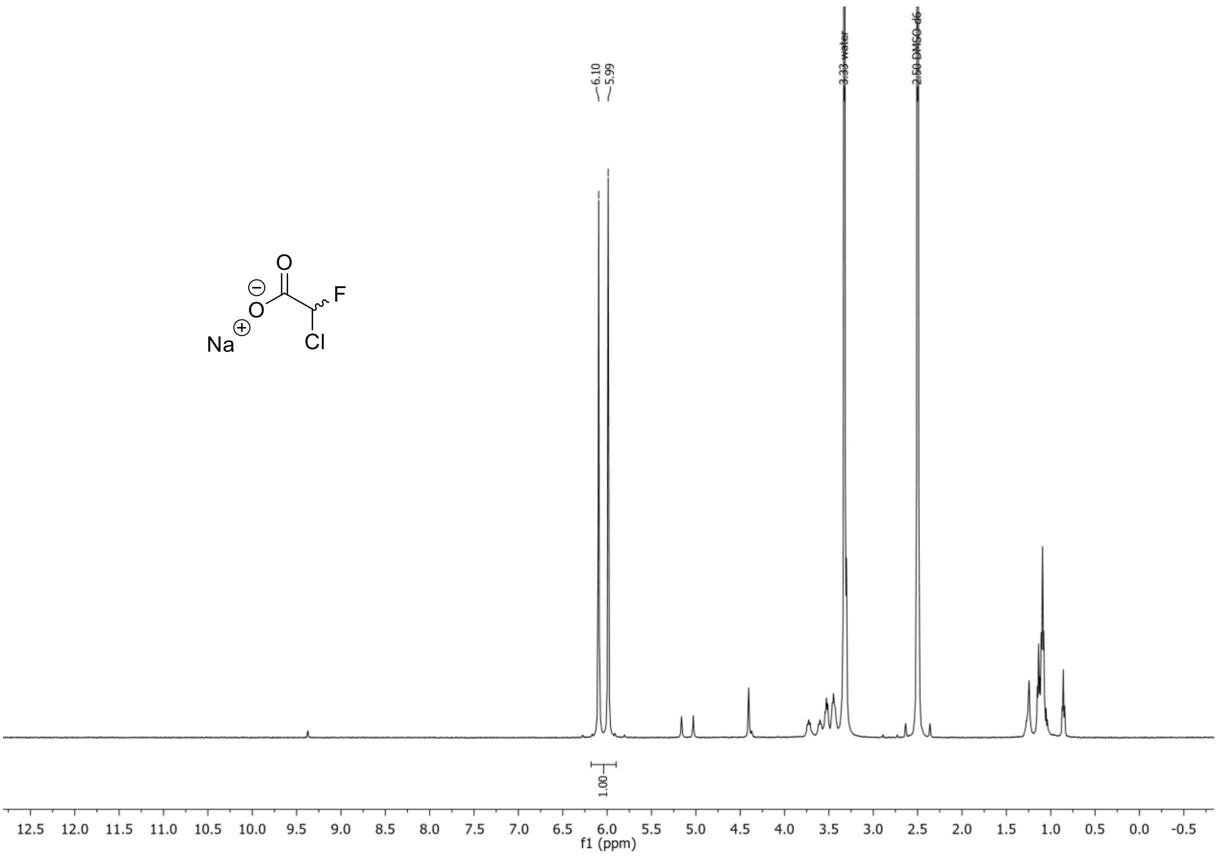

^{13}C NMR {^{1}H} (126 MHz, DMSO-d_6)

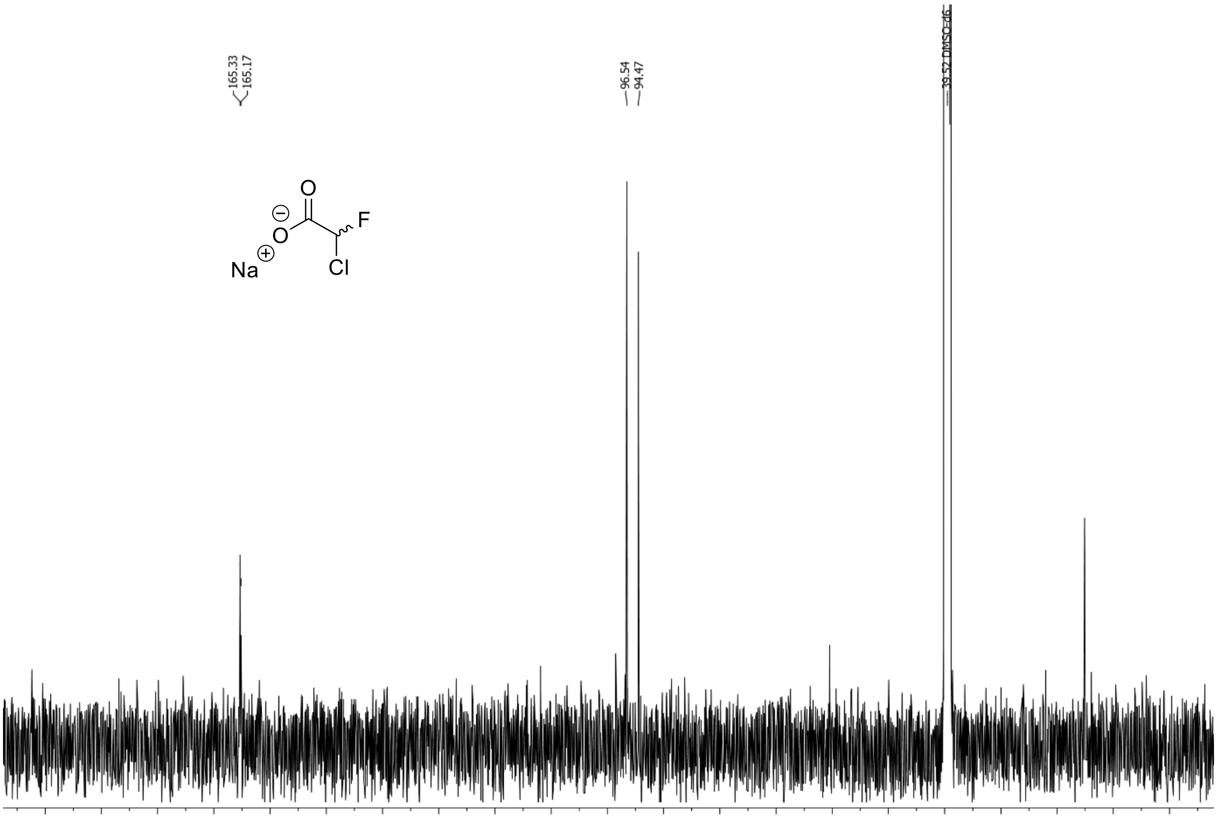

^{19}F NMR (471 MHz, DMSO-d_6)

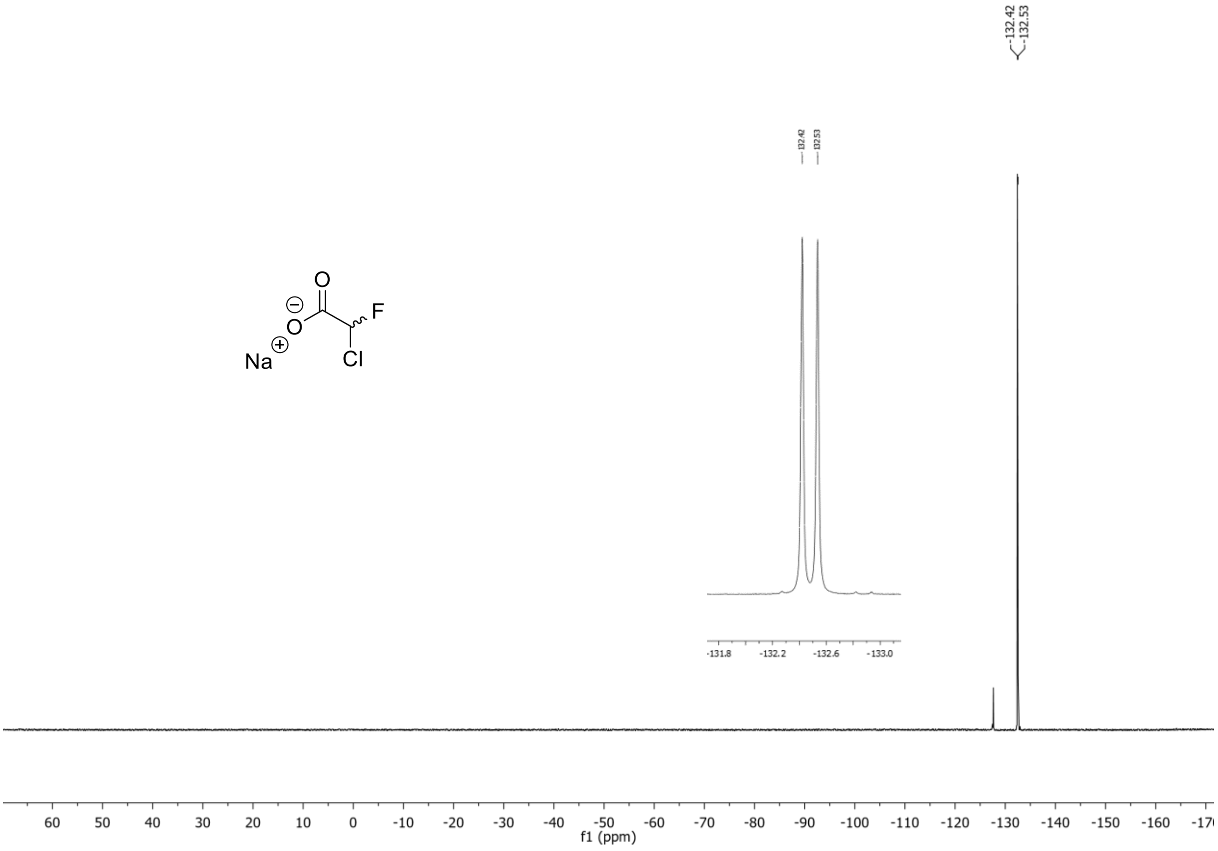

Compound 17

^{1}H NMR (500 MHz, DMSO-d_6)

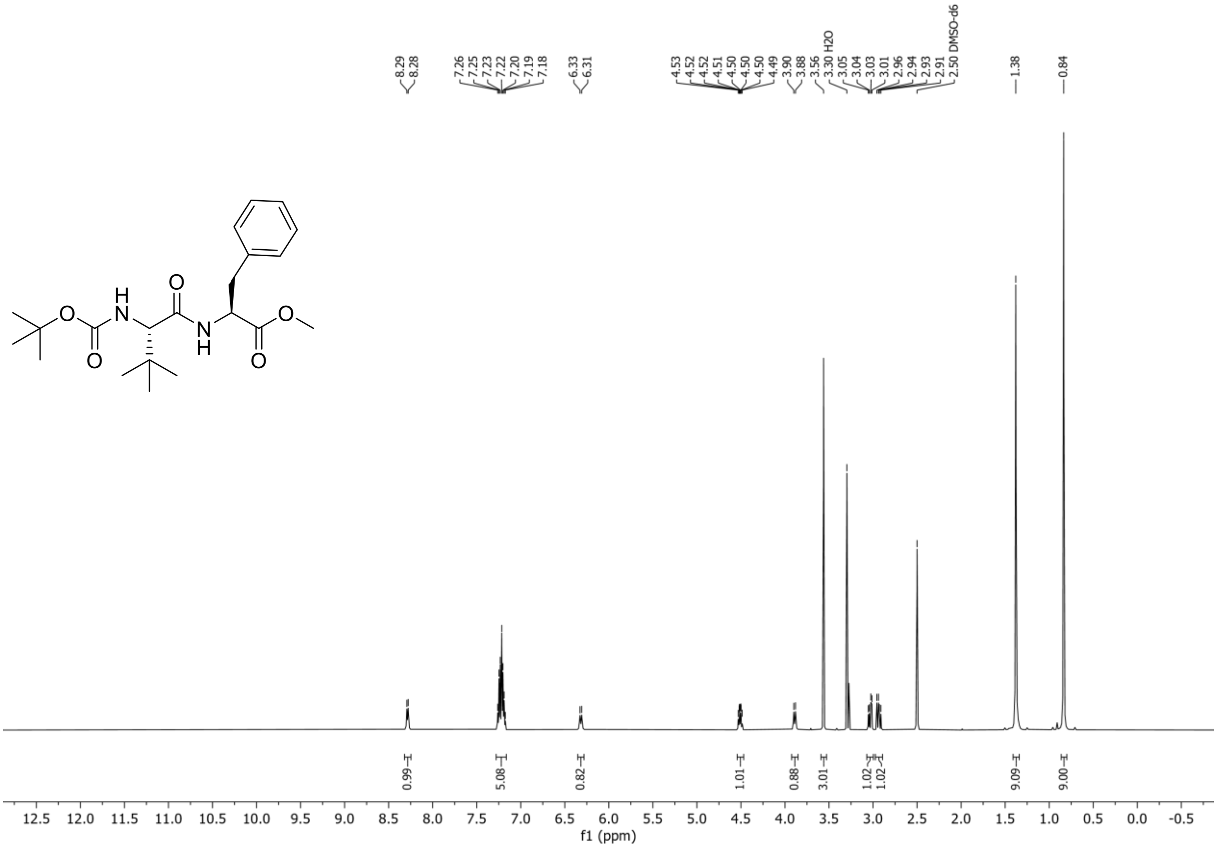

^{13}C NMR {^{1}H} (126 MHz, DMSO-d_6)

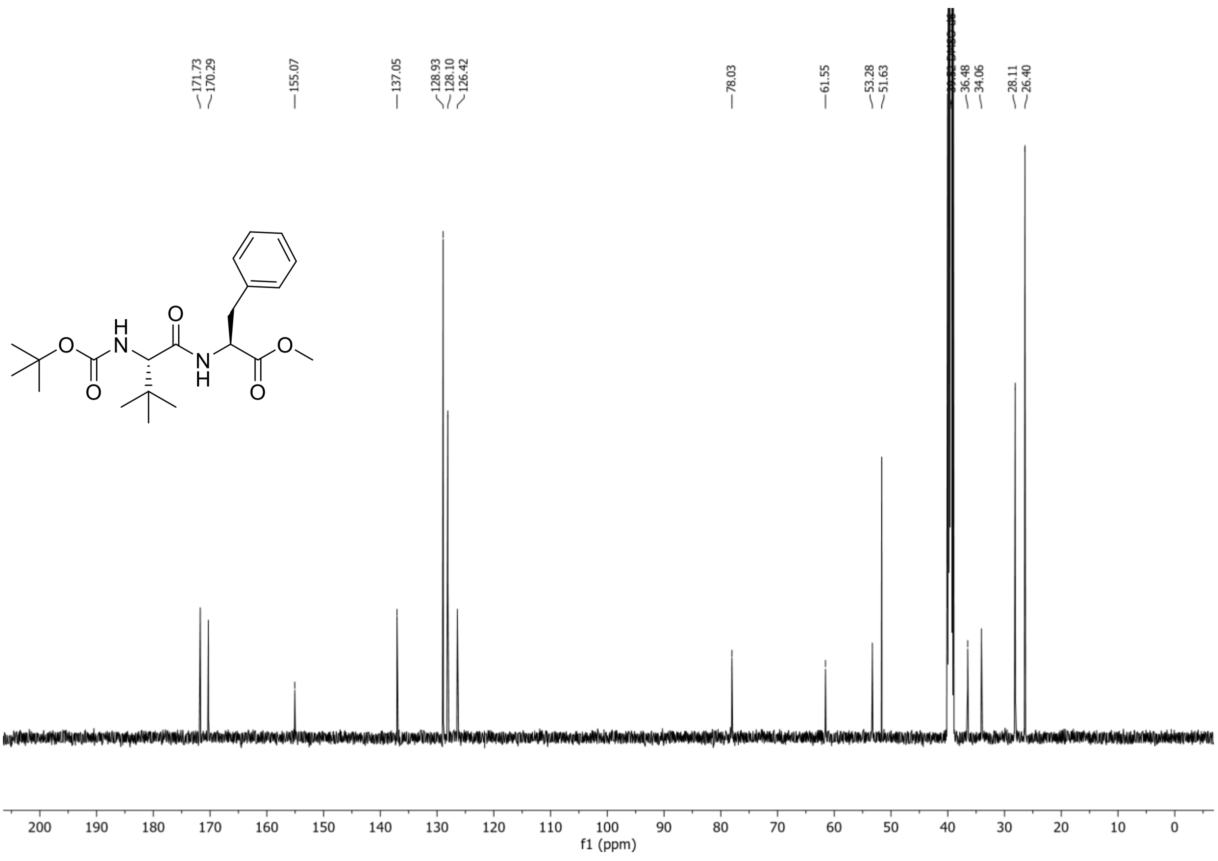

Compound 18

^{1}H NMR (600 MHz, DMSO-d_6)

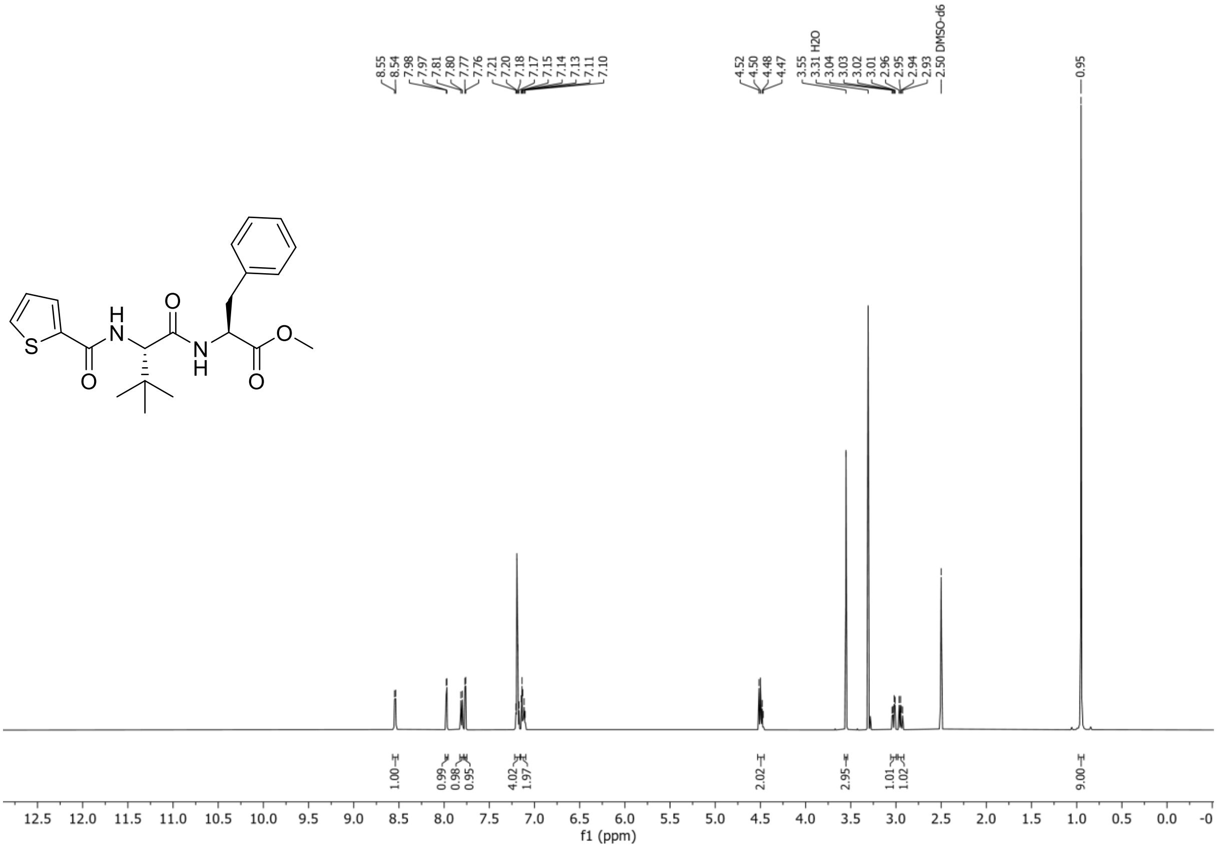

^{13}C NMR {^{1}H} (151 MHz, DMSO-d_6)

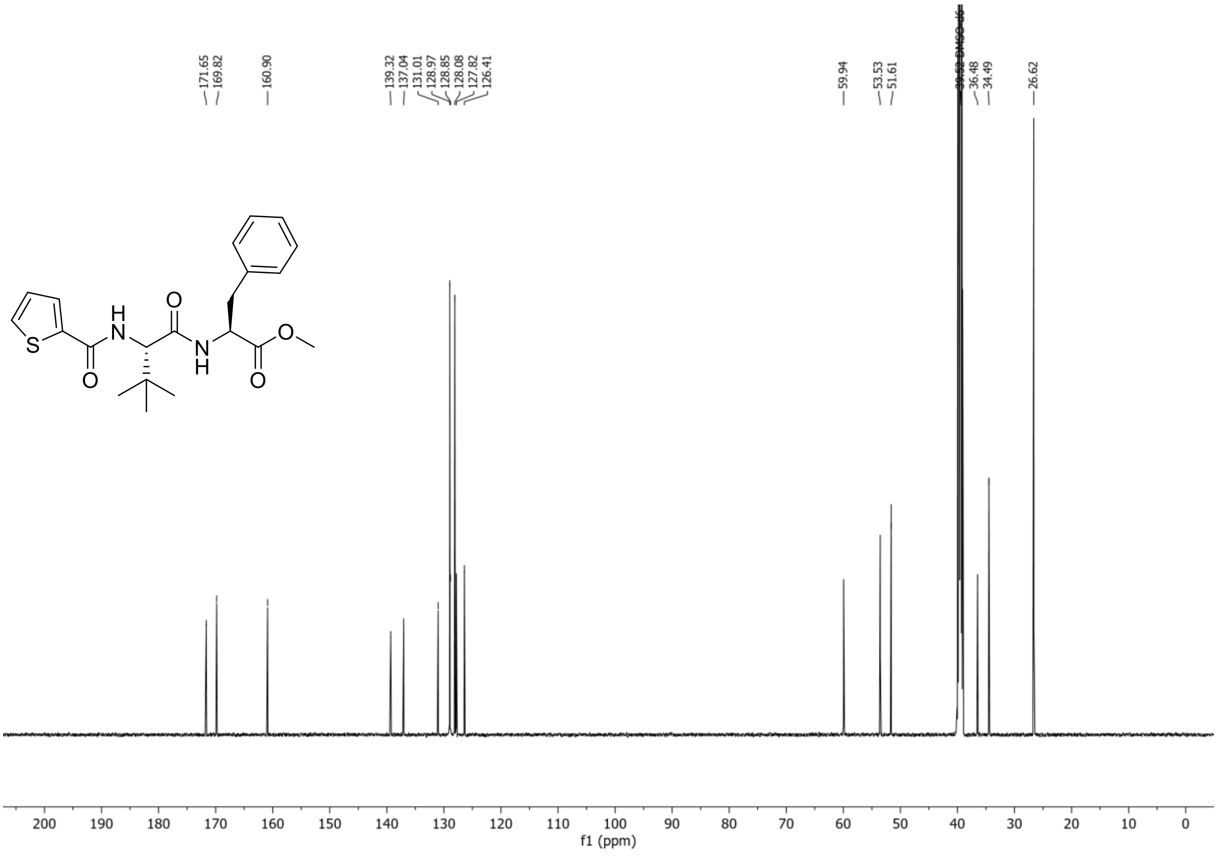

Compound 19

^{1}H NMR (600 MHz, DMSO-d_6)

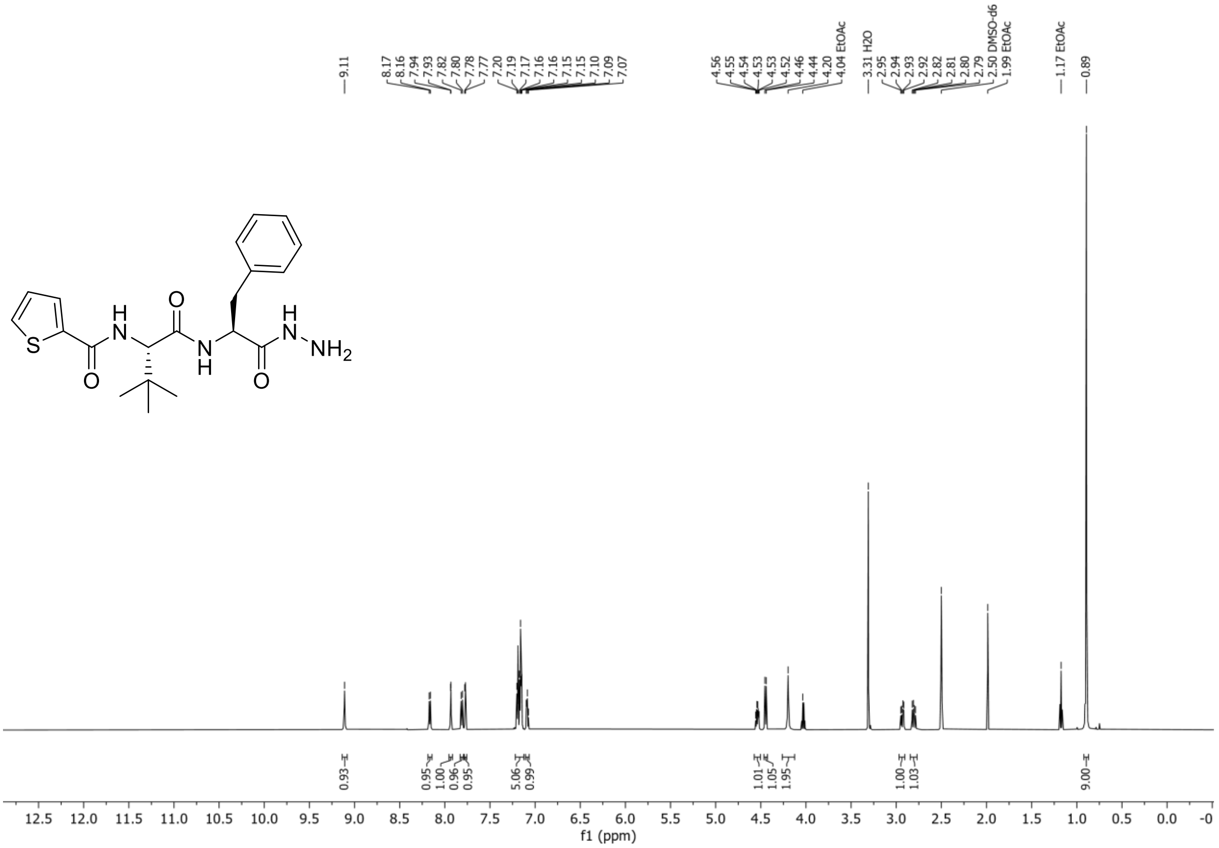

^{13}C NMR {^{1}H} (151 MHz, DMSO-d_6)

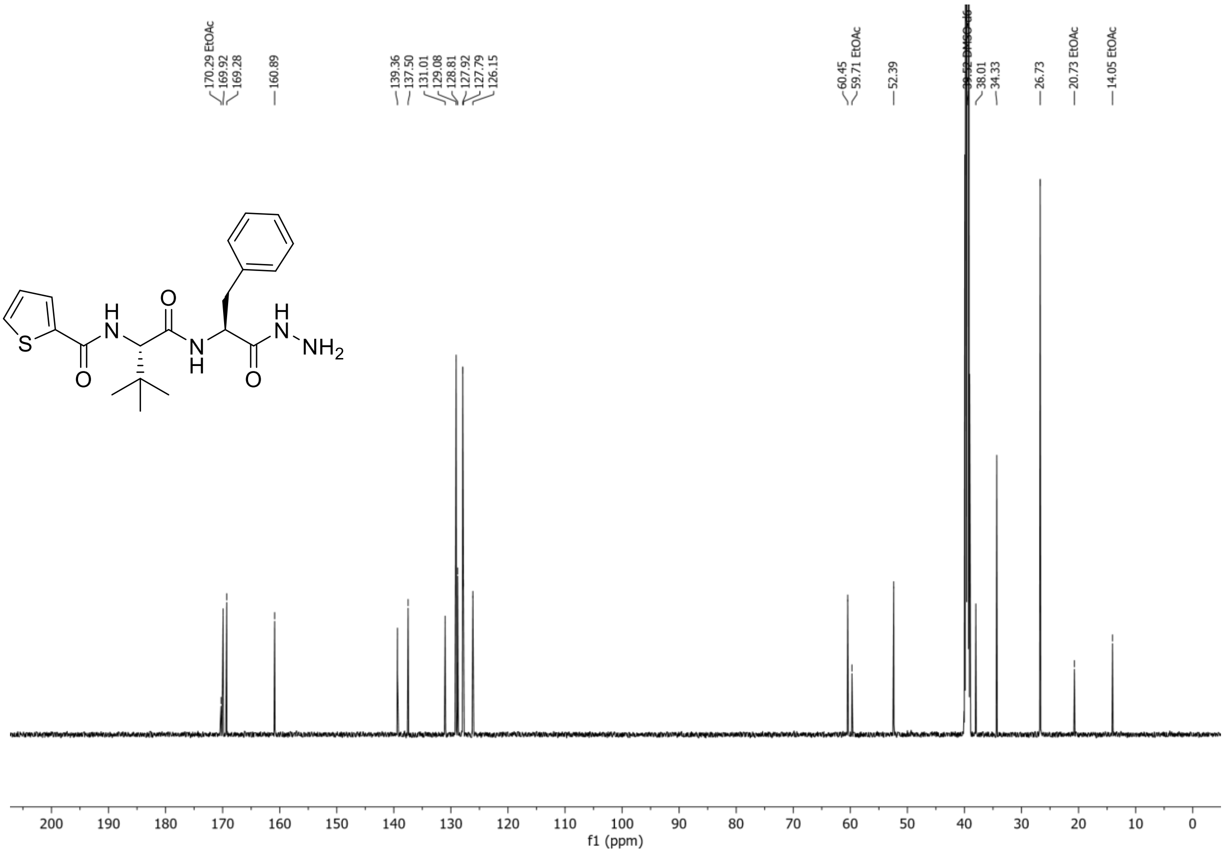

Compound 20

^{1}H NMR (500 MHz, DMSO-d_6)

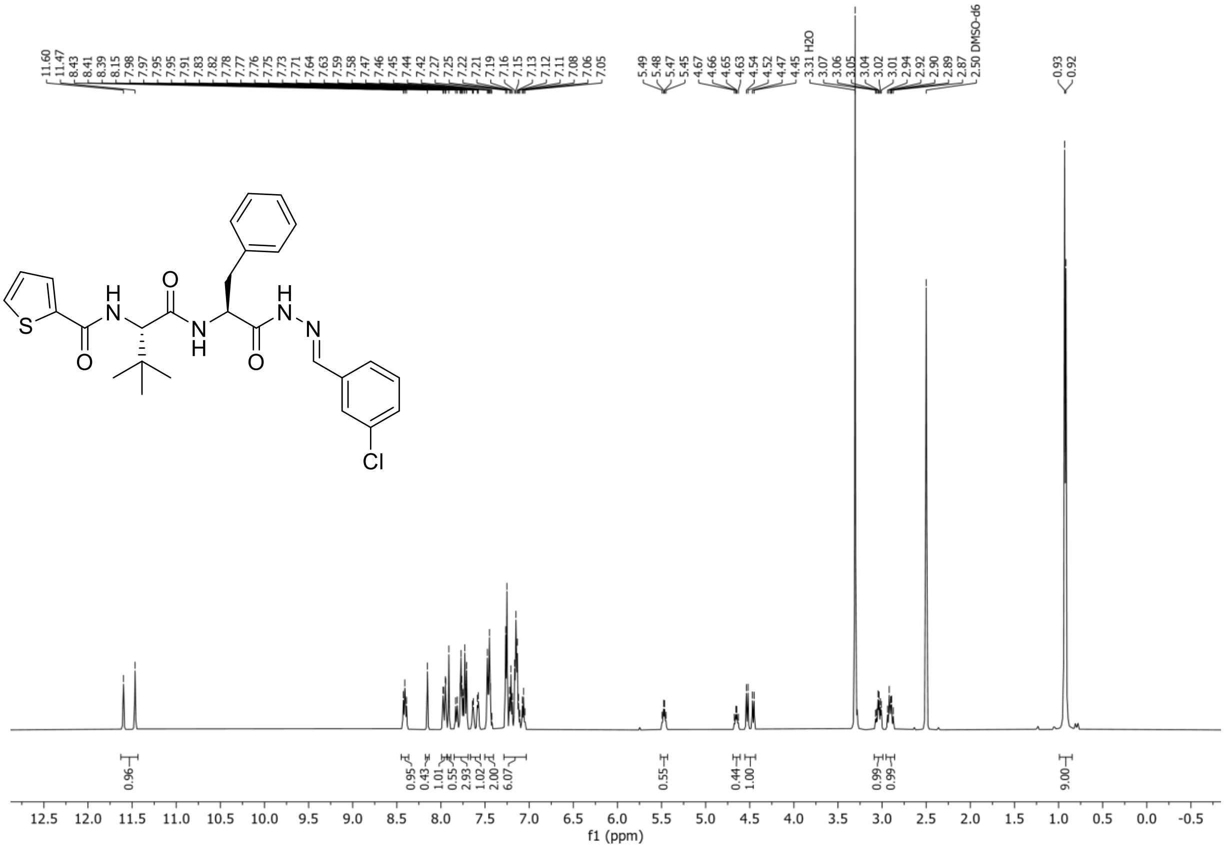

^{13}C NMR {^{1}H} (126 MHz, DMSO-d_6)

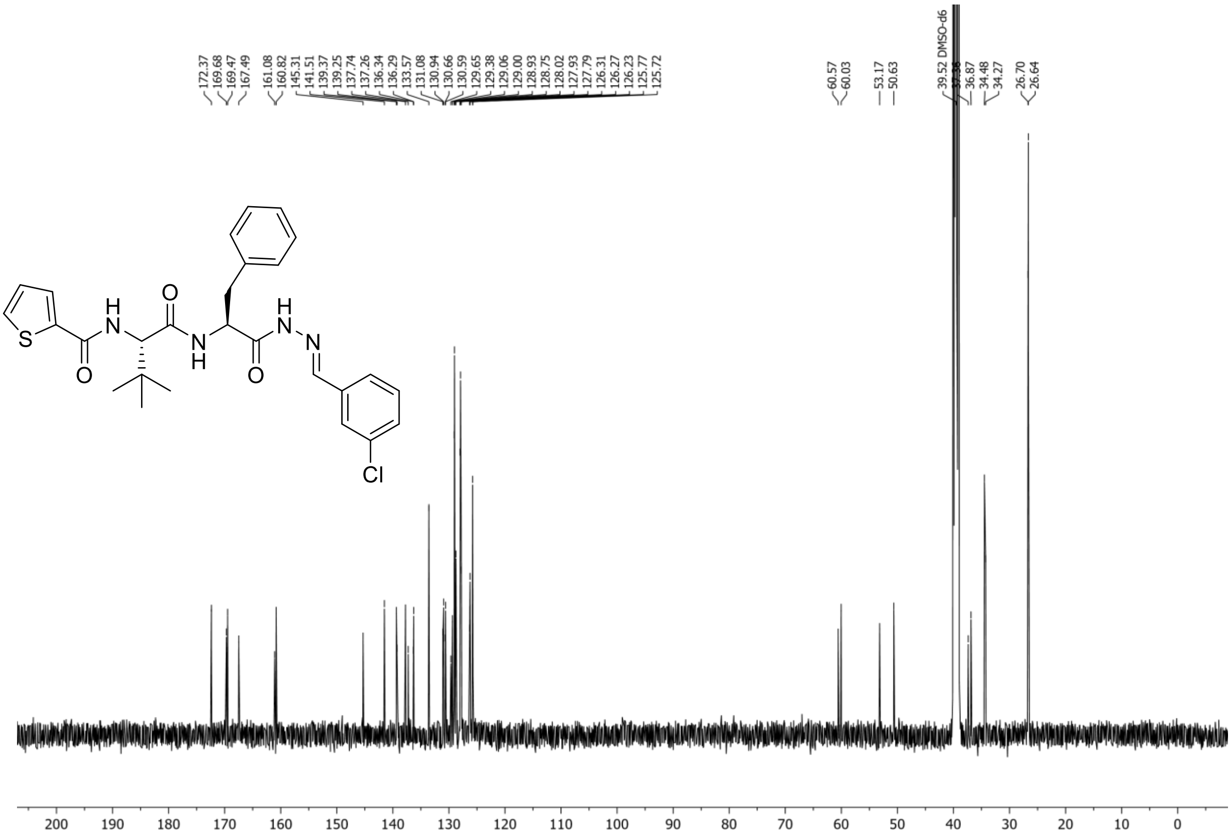

Compound 21

^{1}H NMR (500 MHz, DMSO-d_6)

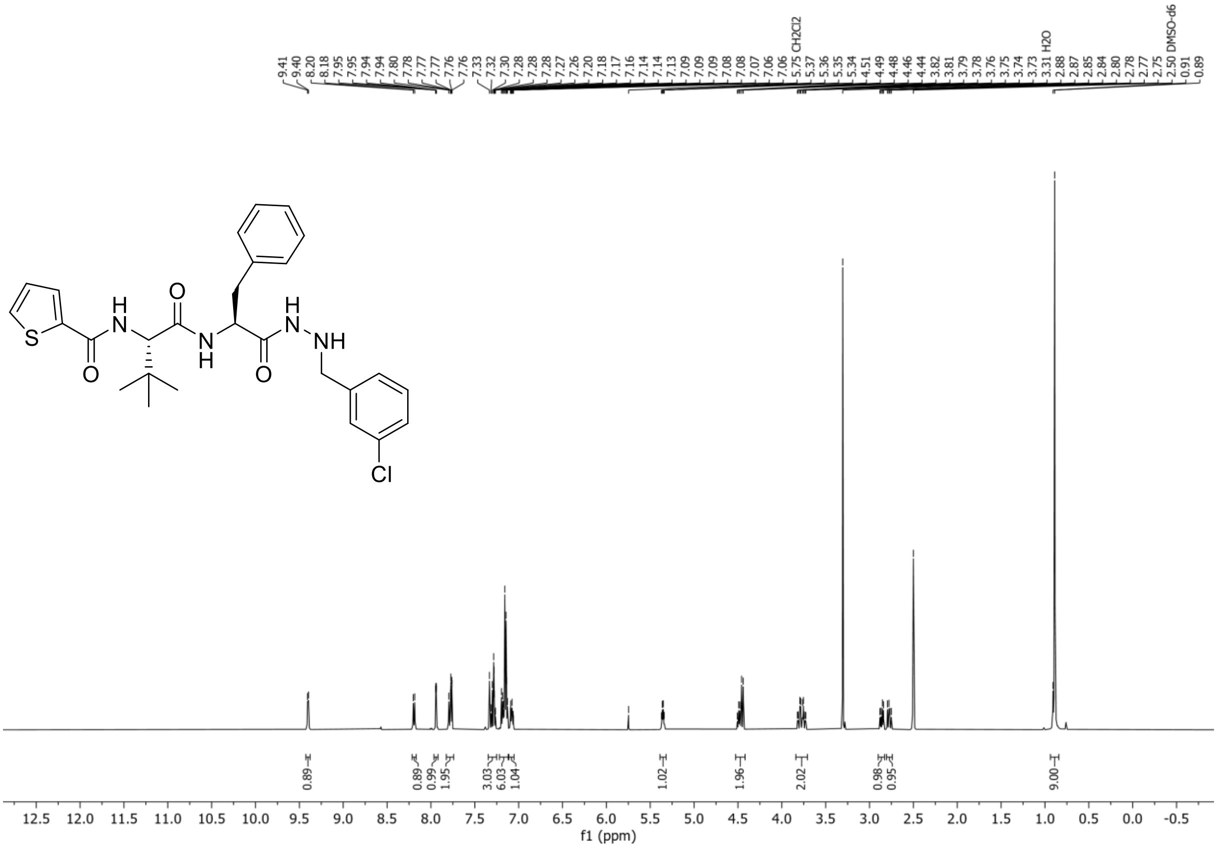

^{13}C NMR {^{1}H} (126 MHz, DMSO-d_6)

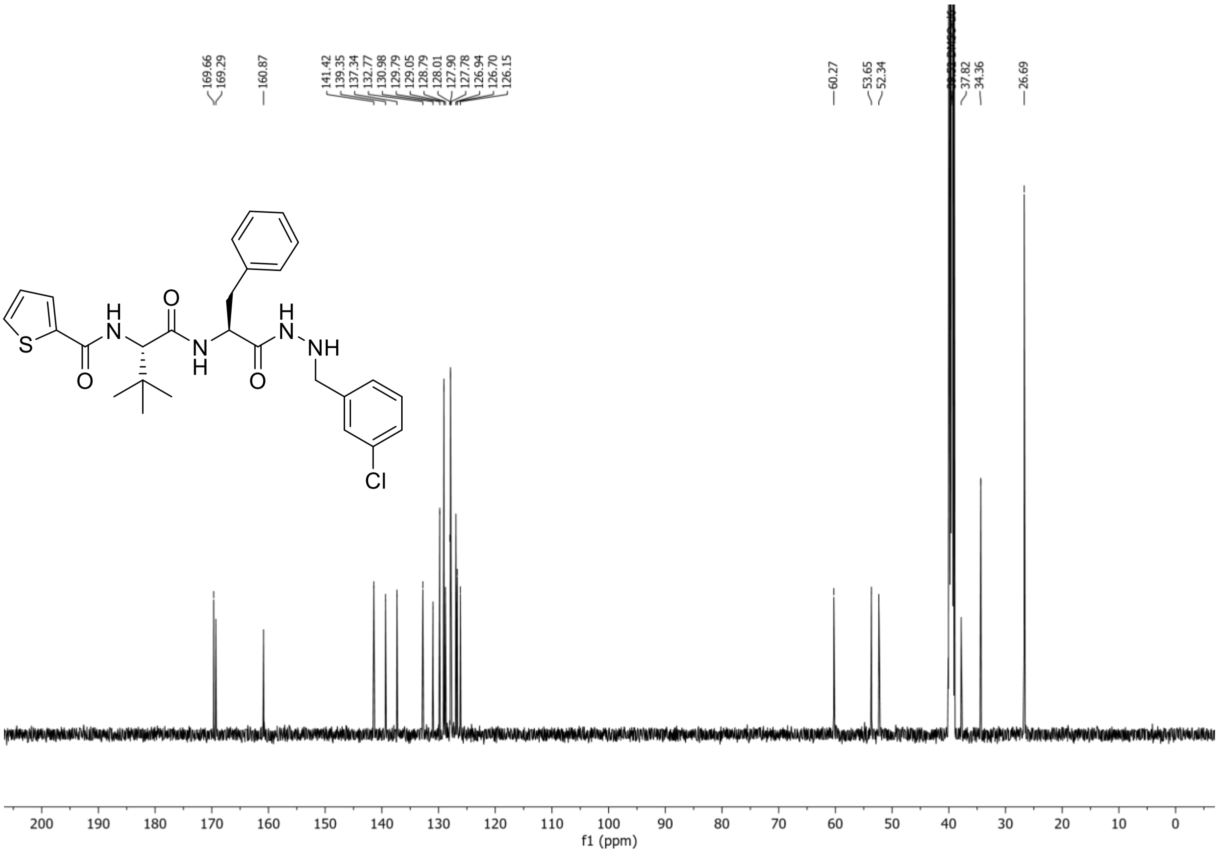

3. HRMS Spectra

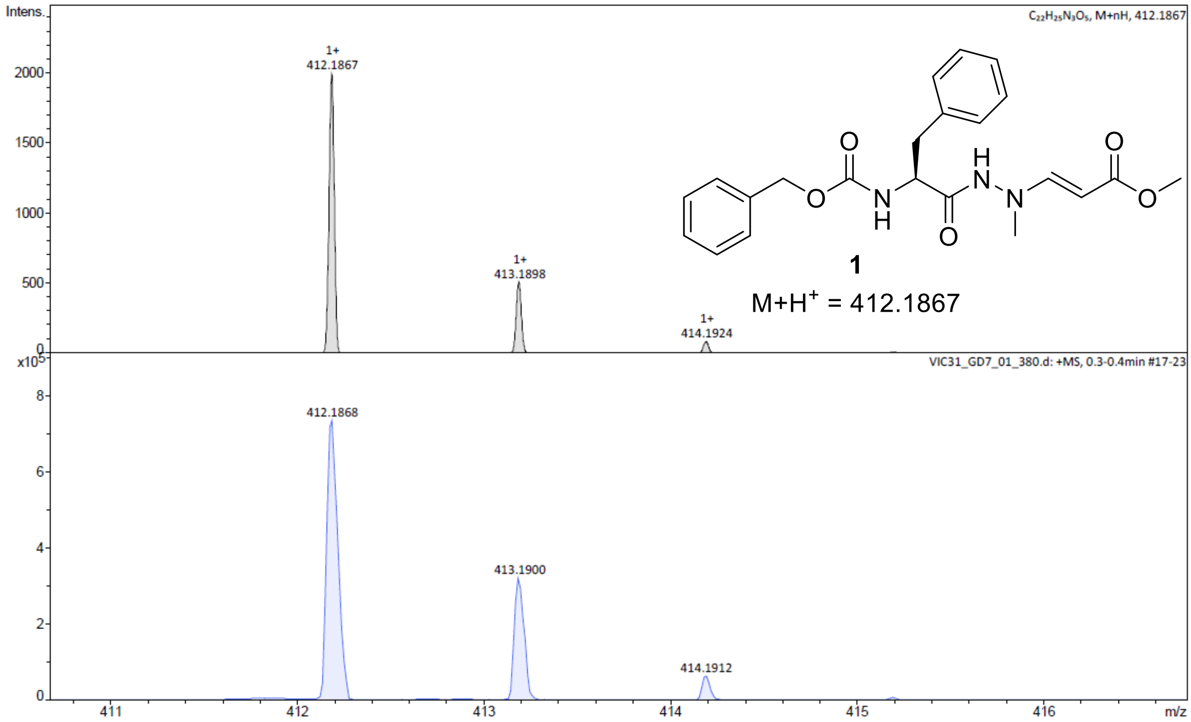

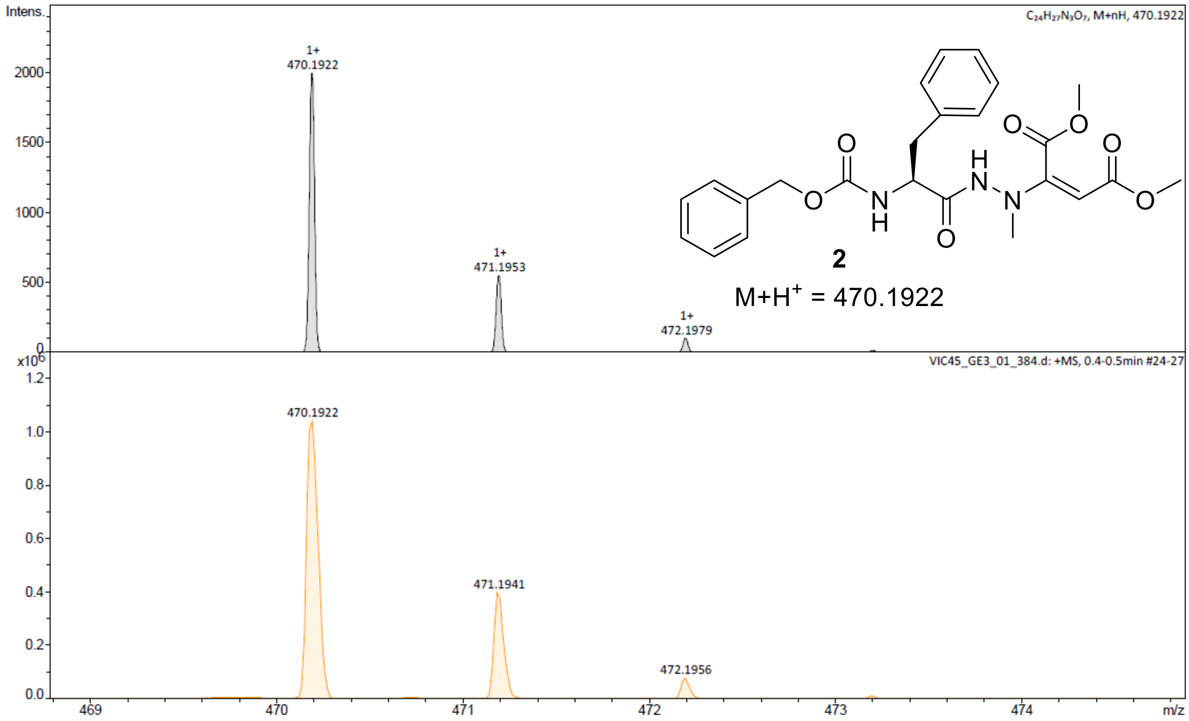

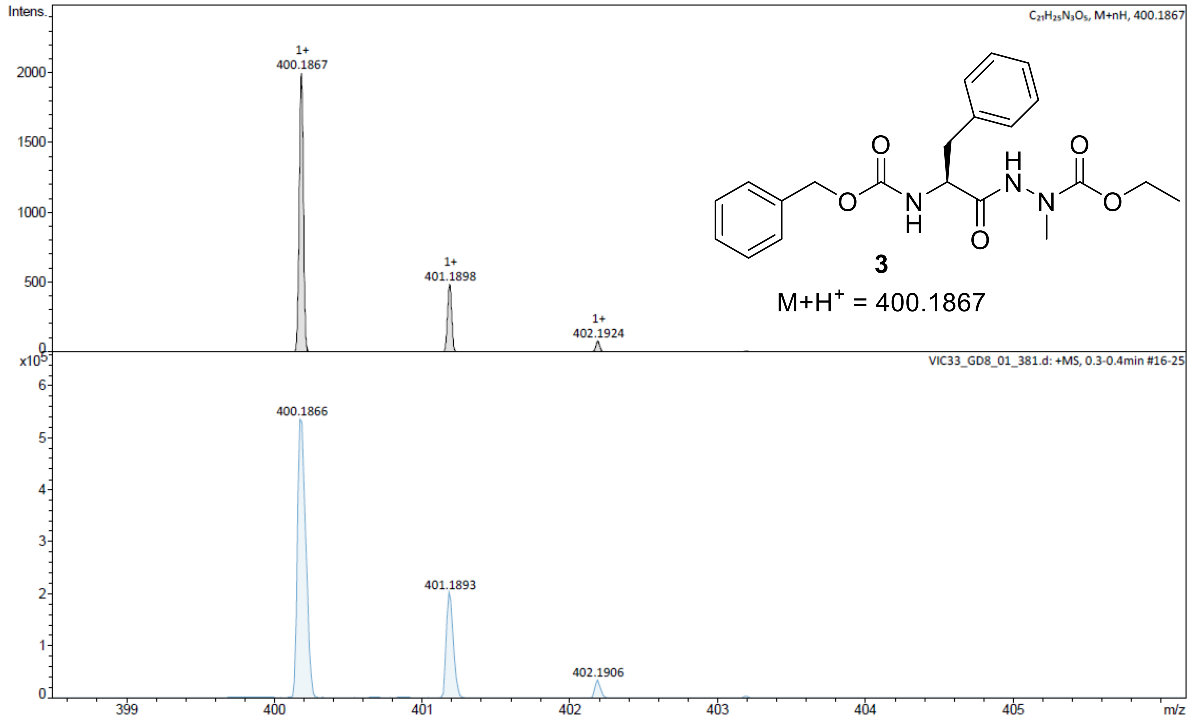
Intens.
C21H25N3O5, M+nH, 400.1867
1+
400.1867
1+
401.1898
1+
402.1924
3
M+H+ = 400.1867
x10^5
VIC33_GD8_01_381.d: +MS, 0.3-0.4min #16-25
400.1866
401.1893
402.1906
m/z

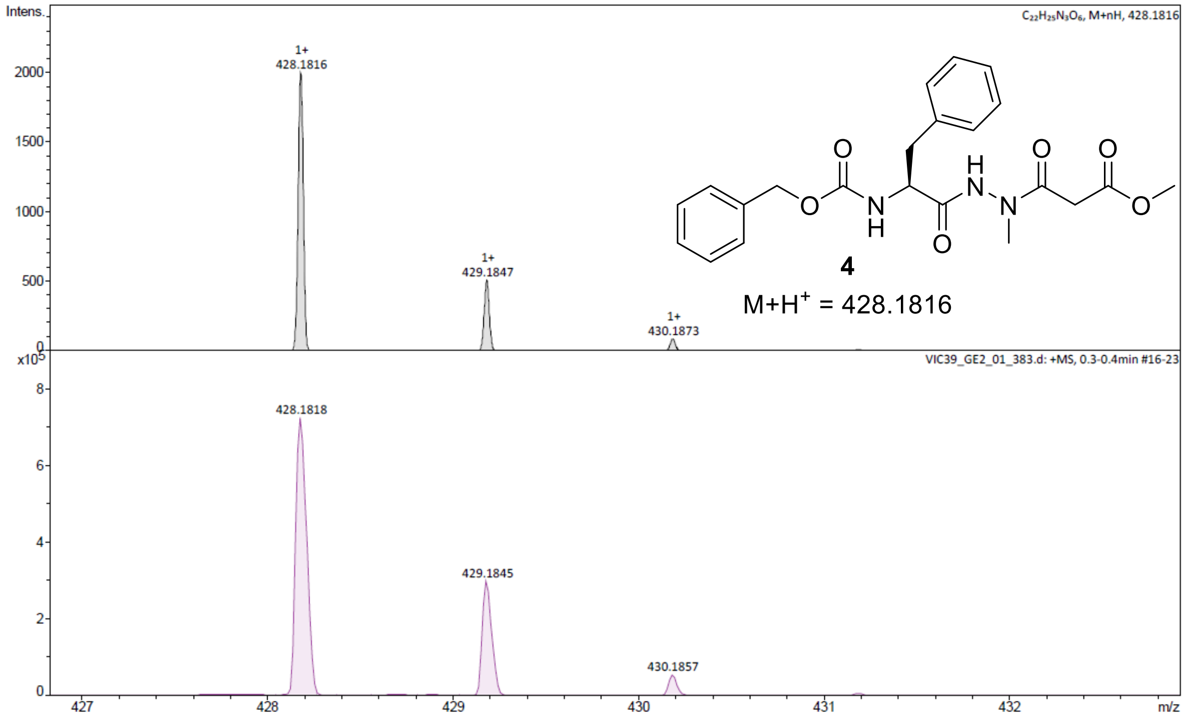
Intens.
C22H25N3O6, M+nH, 428.1816
1+
428.1816
1+
429.1847
1+
430.1873
4
M+H+ = 428.1816
x10^5
VIC39_GE2_01_383.d: +MS, 0.3-0.4min #16-23
428.1818
429.1845
430.1857
m/z

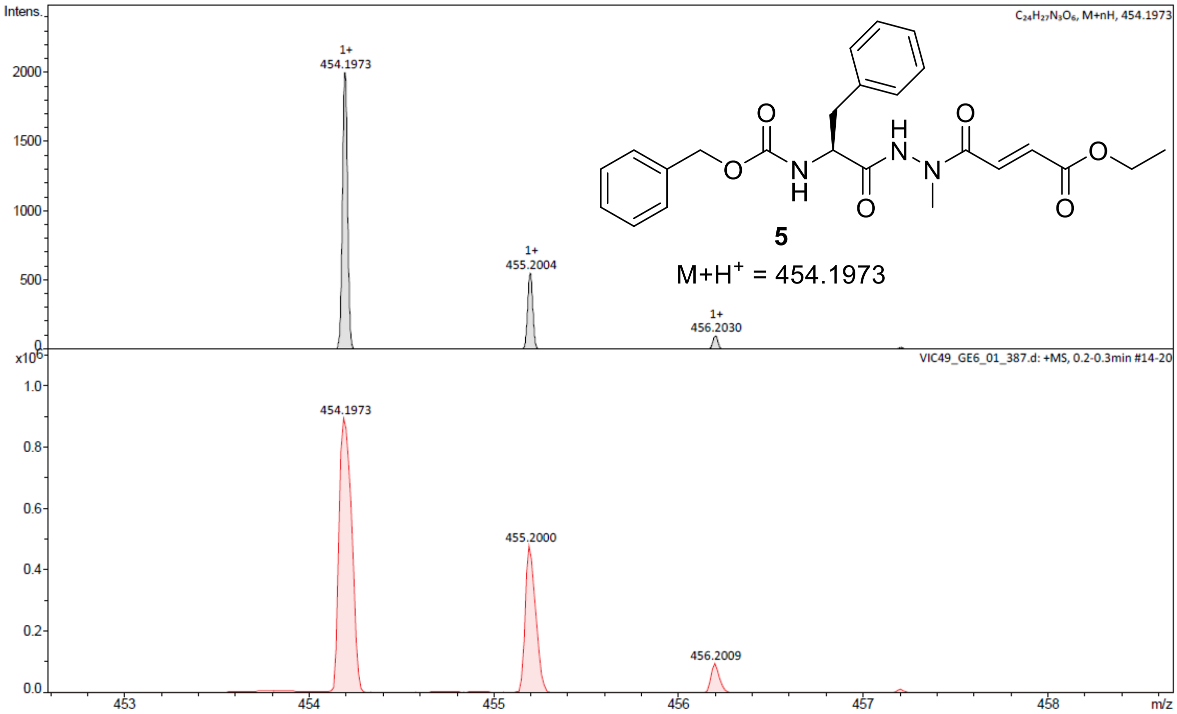

C24H27N3O6, M+nH, 454.1973
VIC49_GE6_01_387.d: +MS, 0.2-0.3min #14-20
Intens.
x10^6
454.1973
455.2004
456.2030
455.2000
456.2009
m/z
5
M+H+ = 454.1973

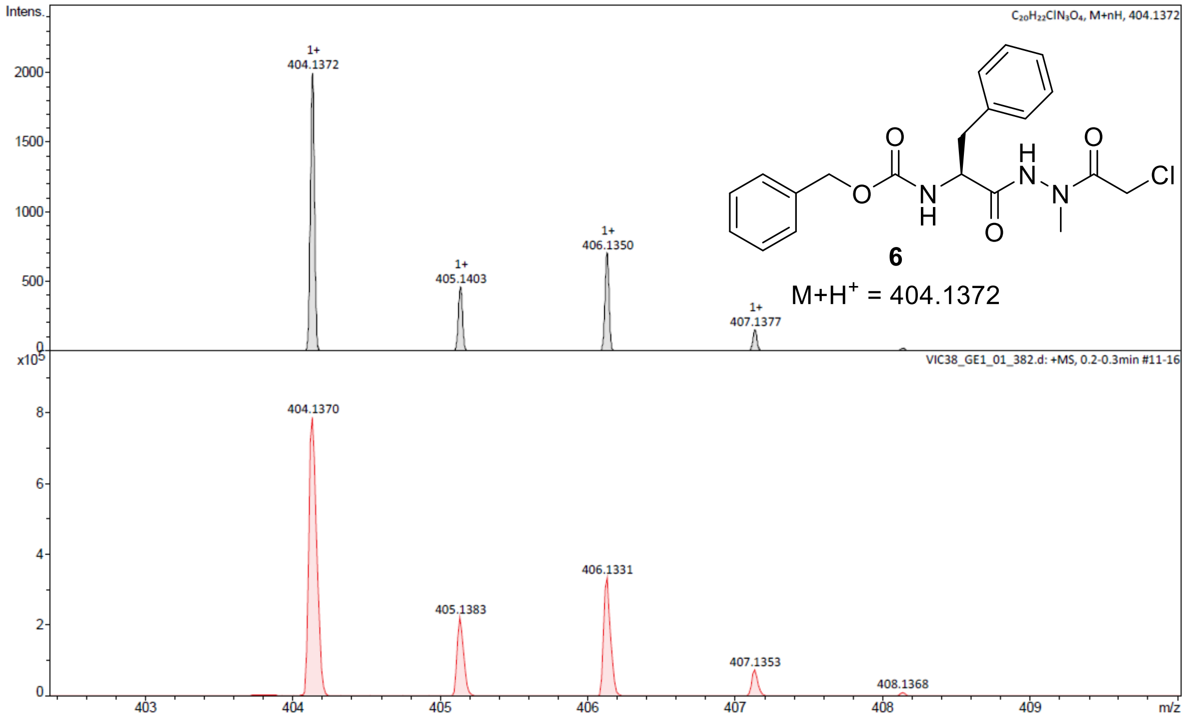

C20H22ClN3O4, M+nH, 404.1372
VIC38_GE1_01_382.d: +MS, 0.2-0.3min #11-16
Intens.
x10^5
404.1372
405.1403
406.1350
407.1377
404.1370
405.1383
406.1331
407.1353
408.1368
m/z
6
M+H+ = 404.1372

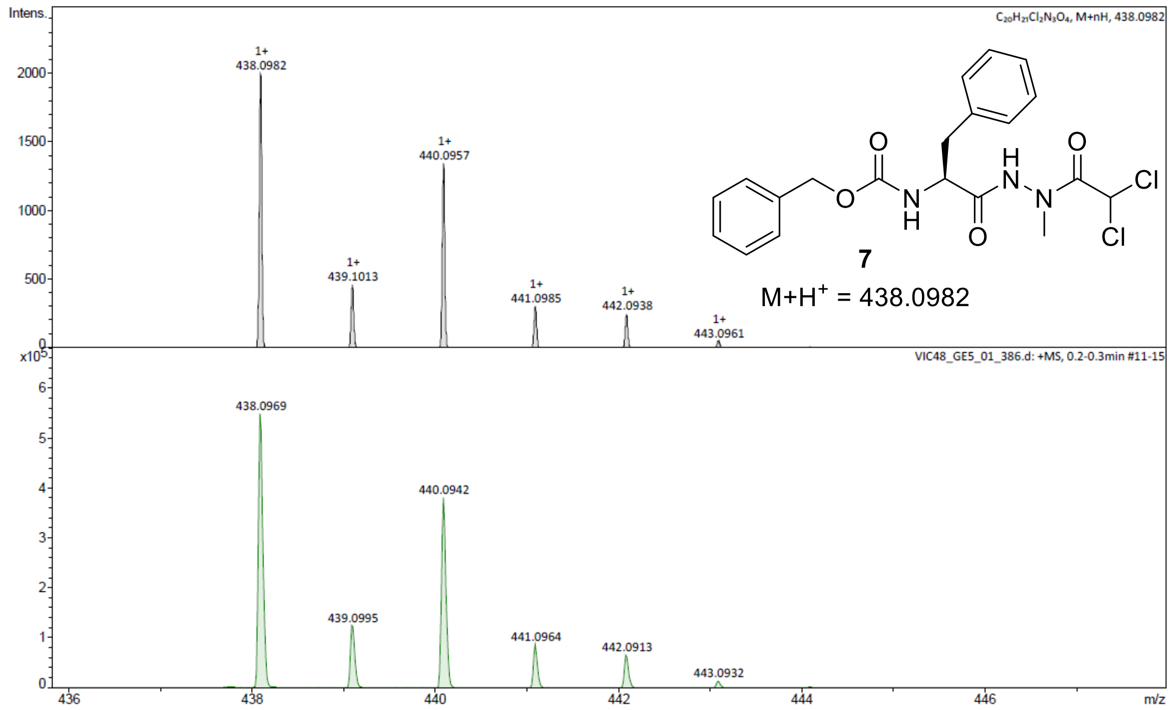

Intens.
C20H21Cl2N3O4, M+nH, 438.0982
1+ 438.0982
1+ 439.1013
1+ 440.0957
1+ 441.0985
1+ 442.0938
1+ 443.0961
7
M+H+ = 438.0982
VIC48_GE5_01_386.d: +MS, 0.2-0.3min #11-15
438.0969
439.0995
440.0942
441.0964
442.0913
443.0932
m/z

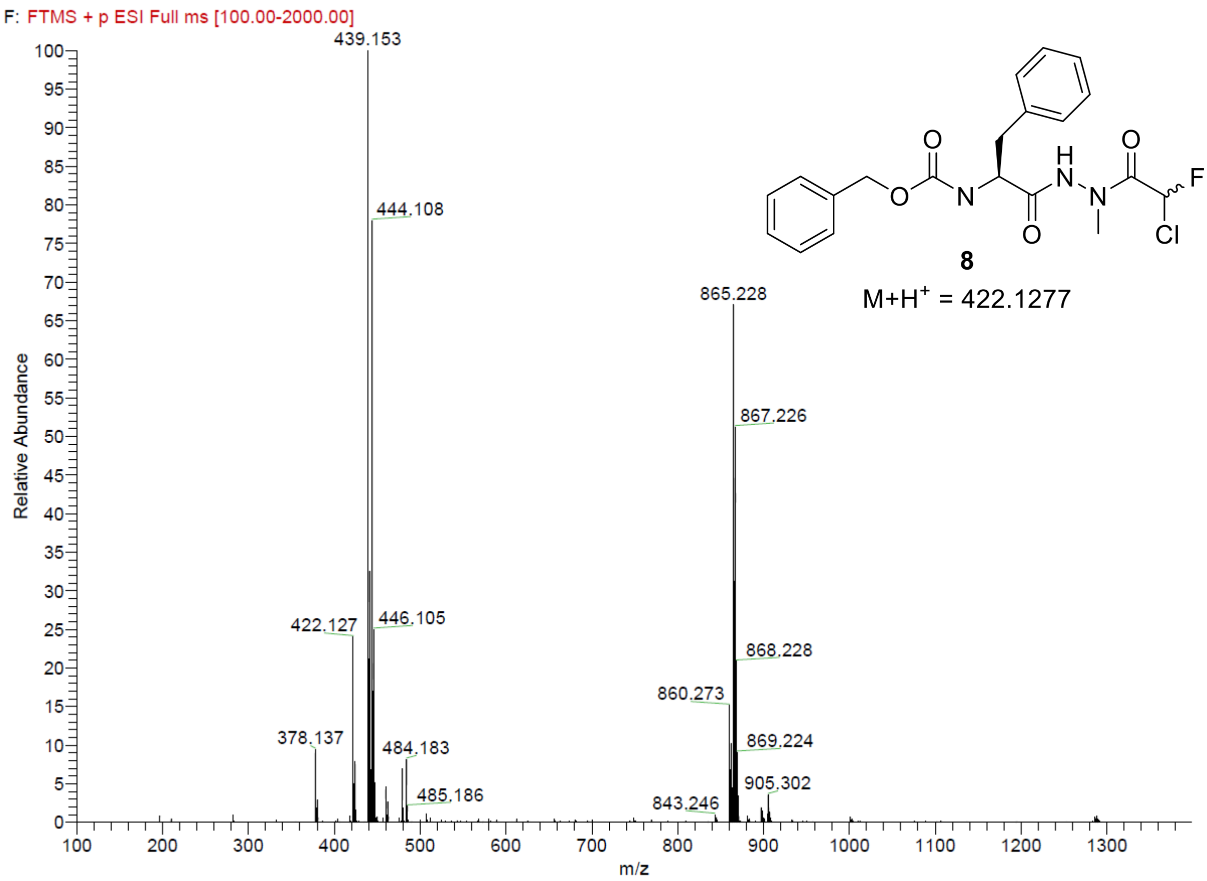
F: FTMS + p ESI Full ms [100.00-2000.00]
Relative Abundance
m/z
439.153
444.108
446.105
422.127
378.137
484.183
485.186
865.228
867.226
868.228
860.273
869.224
905.302
843.246
8
M+H+ = 422.1277

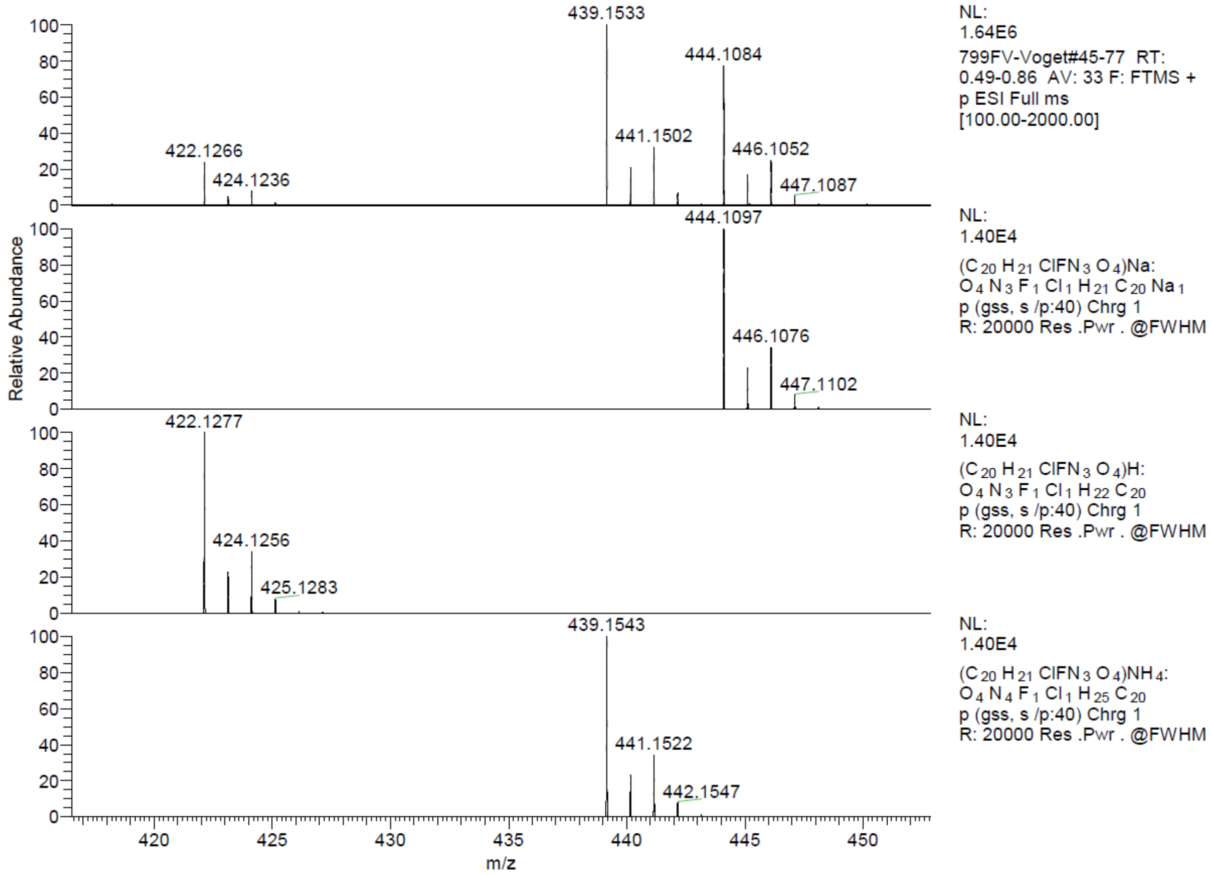
439.1533
444.1084
441.1502
422.1266
446.1052
424.1236
447.1087
NL: 1.64E6
799FV-Voget#45-77 RT: 0.49-0.86 AV: 33 F: FTMS + p ESI Full ms [100.00-2000.00]
444.1097
446.1076
447.1102
NL: 1.40E4
(C20 H21 ClFN3 O4)Na: O4 N3 F1 Cl1 H21 C20 Na1 p (gss, s /p:40) Chrg 1 R: 20000 Res .Pwr . @FWHM
422.1277
424.1256
425.1283
NL: 1.40E4
(C20 H21 ClFN3 O4)H: O4 N3 F1 Cl1 H22 C20 p (gss, s /p:40) Chrg 1 R: 20000 Res .Pwr . @FWHM
439.1543
441.1522
442.1547
NL: 1.40E4
(C20 H21 ClFN3 O4)NH4: O4 N4 F1 Cl1 H25 C20 p (gss, s /p:40) Chrg 1 R: 20000 Res .Pwr . @FWHM
Relative Abundance
m/z

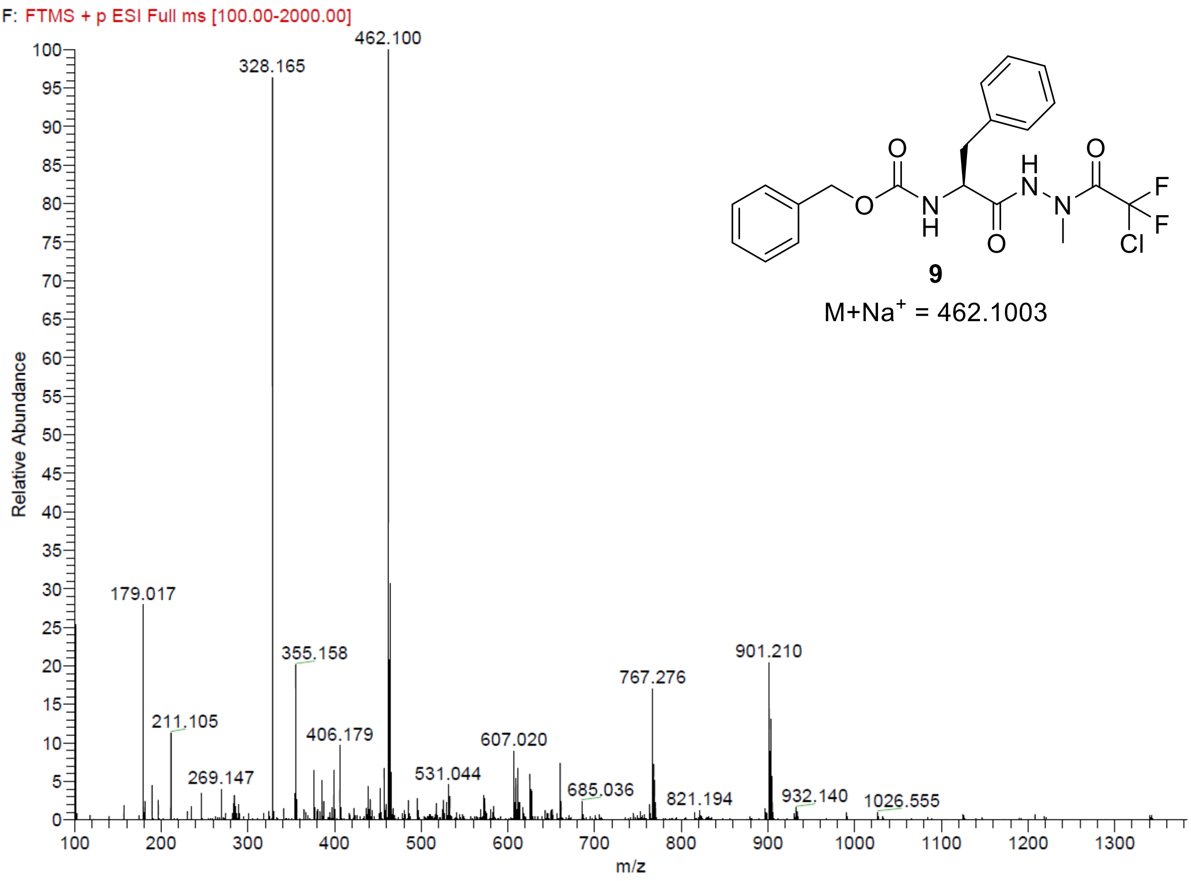

F: FTMS + p ESI Full ms [100.00-2000.00]
Relative Abundance
m/z
462.100
328.165
179.017
355.158
211.105
406.179
269.147
607.020
531.044
767.276
685.036
821.194
901.210
932.140
1026.555
9
M+Na+ = 462.1003

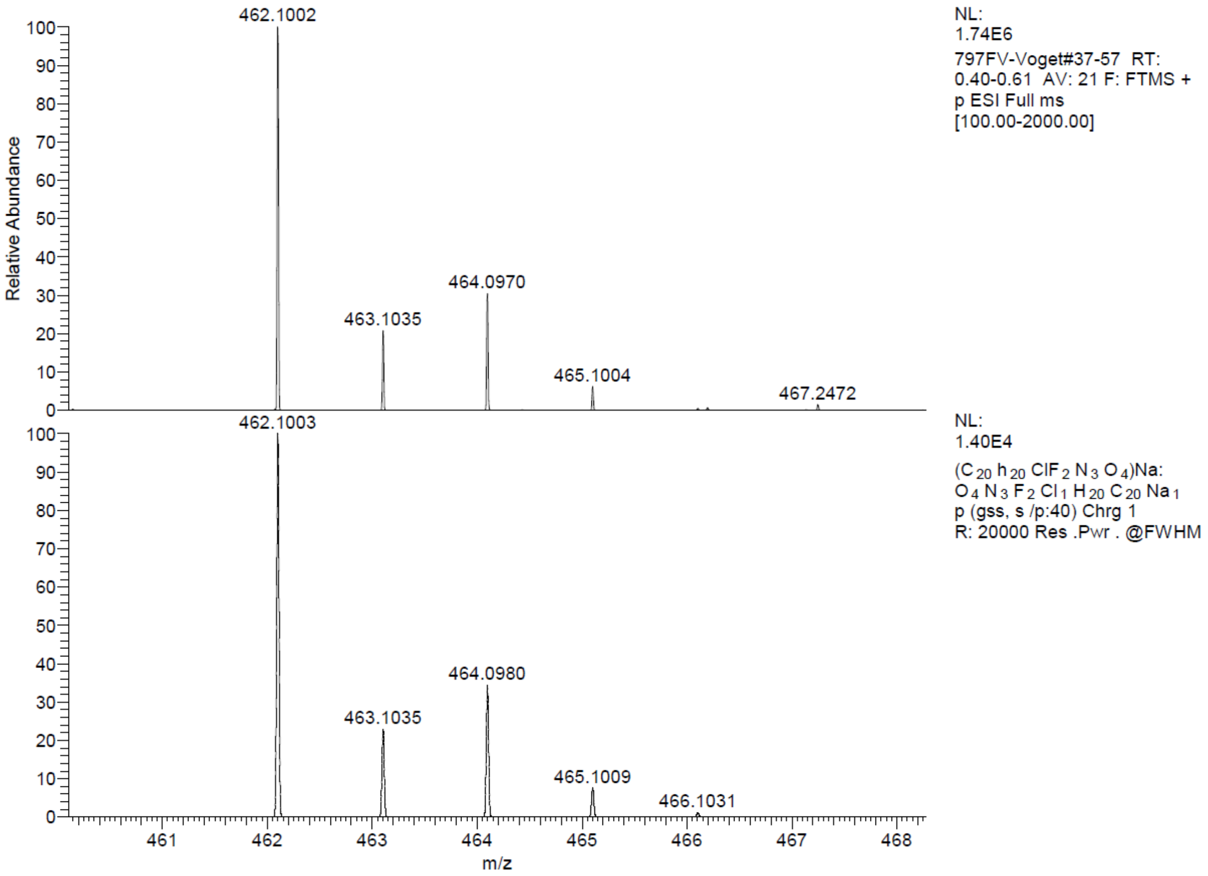

462.1002
463.1035
464.0970
465.1004
467.2472
NL:
1.74E6
797FV-Voget#37-57 RT:
0.40-0.61 AV: 21 F: FTMS +
p ESI Full ms
[100.00-2000.00]
462.1003
463.1035
464.0980
465.1009
466.1031
NL:
1.40E4
(C 20 h 20 ClF 2 N 3 O 4)Na:
O 4 N 3 F 2 Cl 1 H 20 C 20 Na 1
p (gss, s /p:40) Chrg 1
R: 20000 Res .Pwr . @FWHM
Relative Abundance
m/z

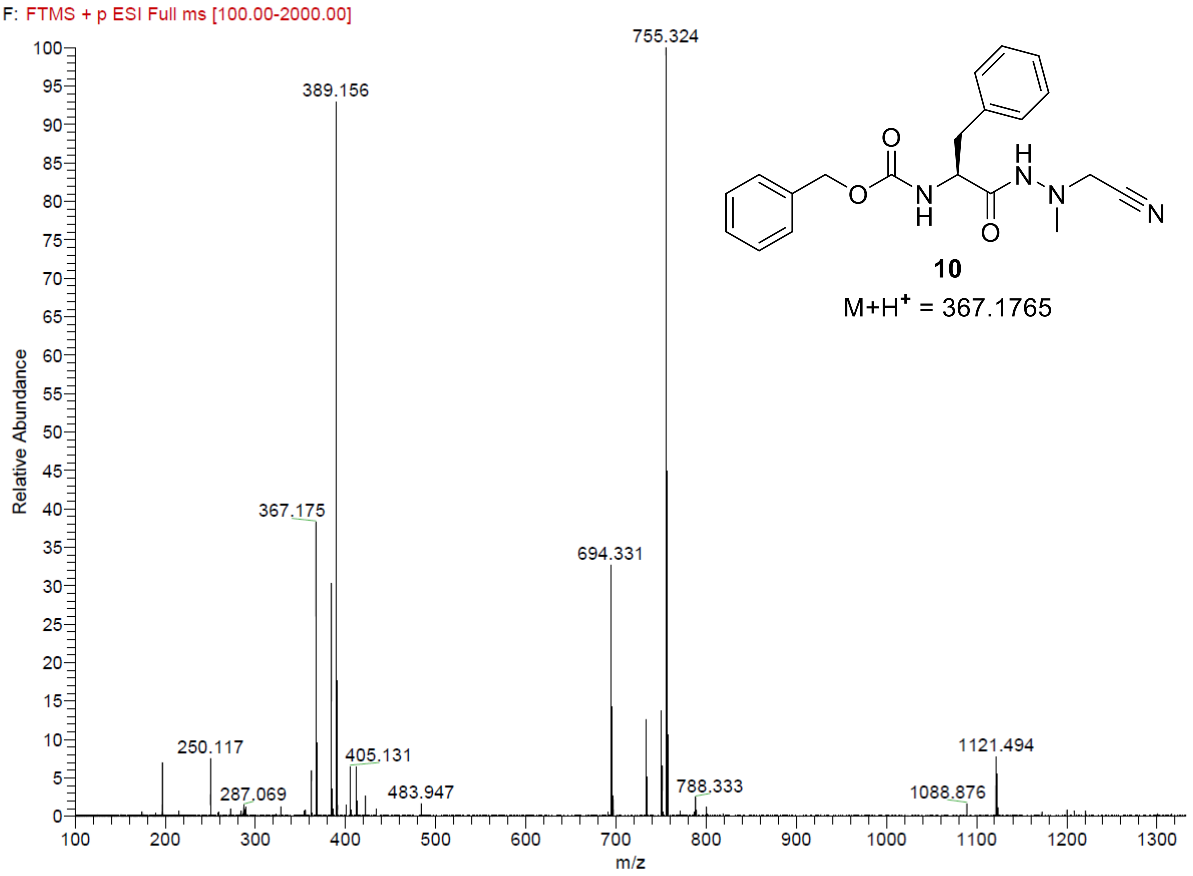
F: FTMS + p ESI Full ms [100.00-2000.00]
Relative Abundance
m/z
755.324
389.156
367.175
694.331
250.117
405.131
287.069
483.947
788.333
1088.876
1121.494
10
M+H+ = 367.1765

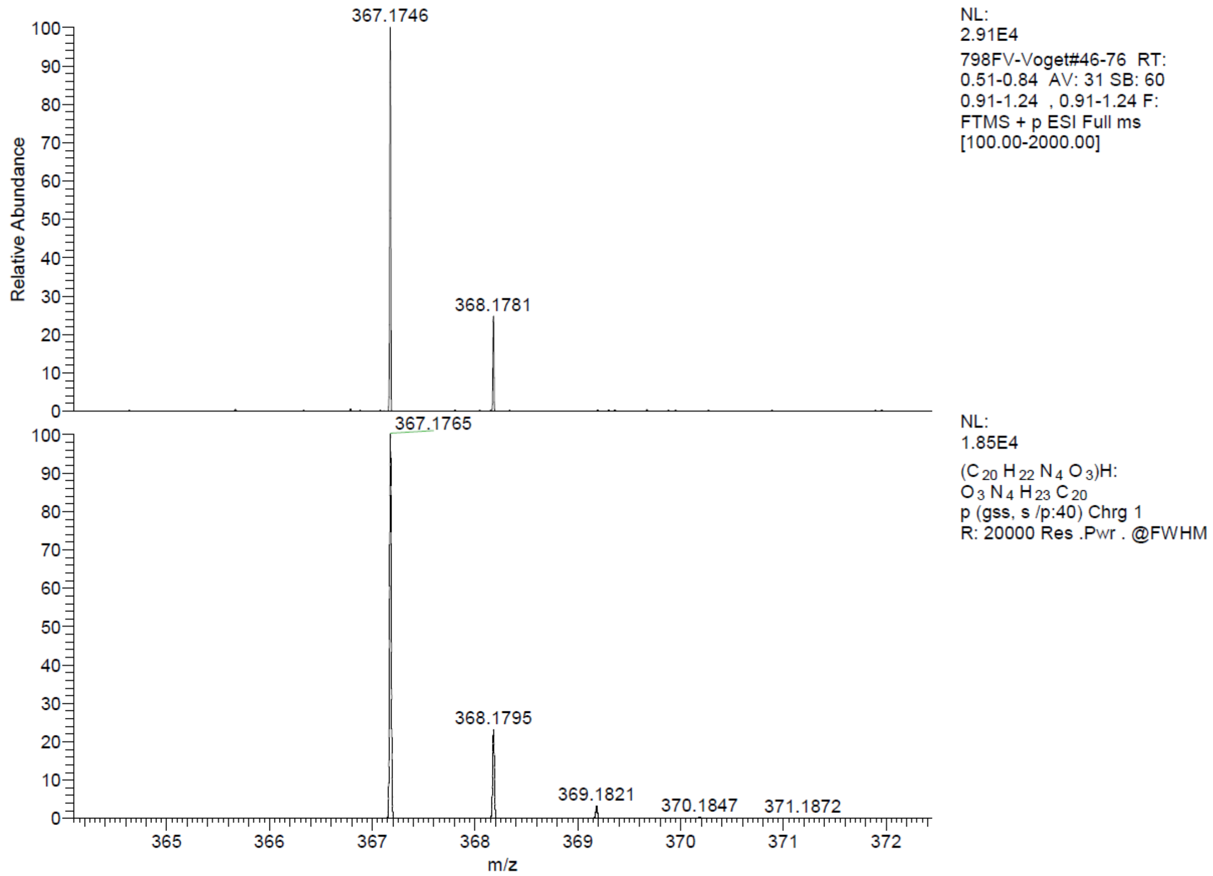
Relative Abundance
m/z
367.1746
368.1781
NL:
2.91E4
798FV-Voget#46-76 RT:
0.51-0.84 AV: 31 SB: 60
0.91-1.24 , 0.91-1.24 F:
FTMS + p ESI Full ms
[100.00-2000.00]
367.1765
368.1795
369.1821
370.1847
371.1872
NL:
1.85E4
(C20 H22 N4 O3)H:
O3 N4 H23 C20
p (gss, s /p:40) Chrg 1
R: 20000 Res .Pwr . @FWHM

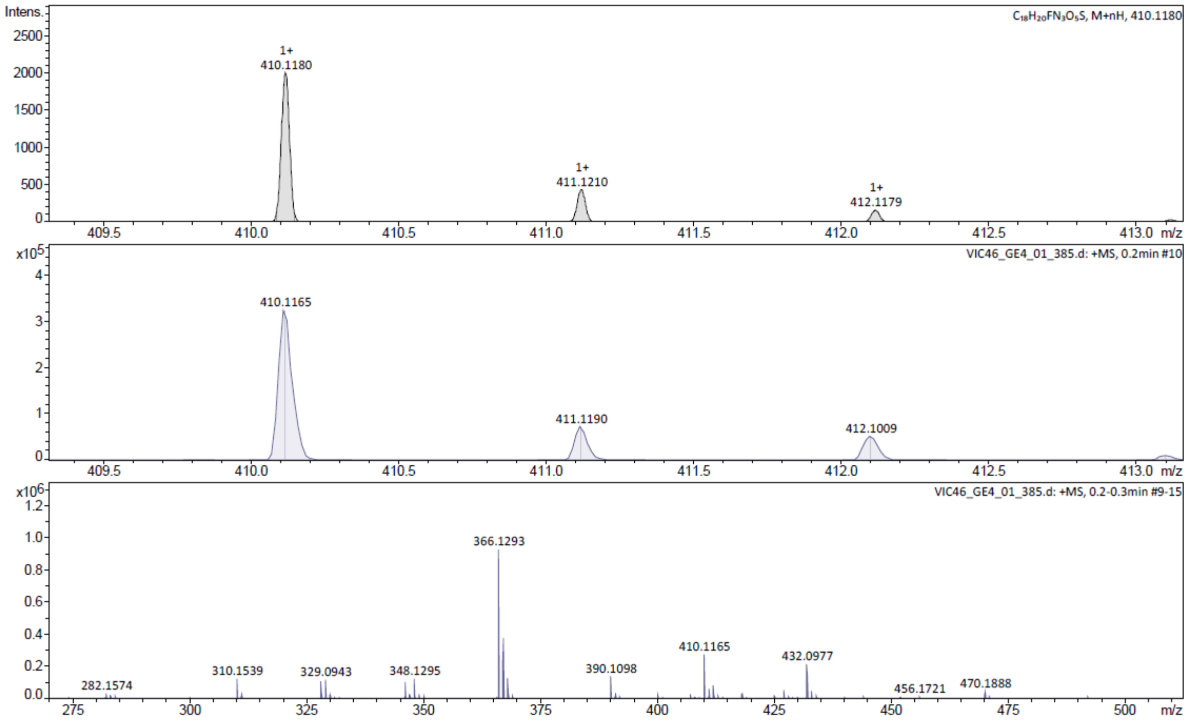

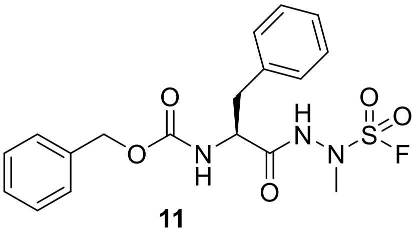

11

M+H$^+$ = 410.1180

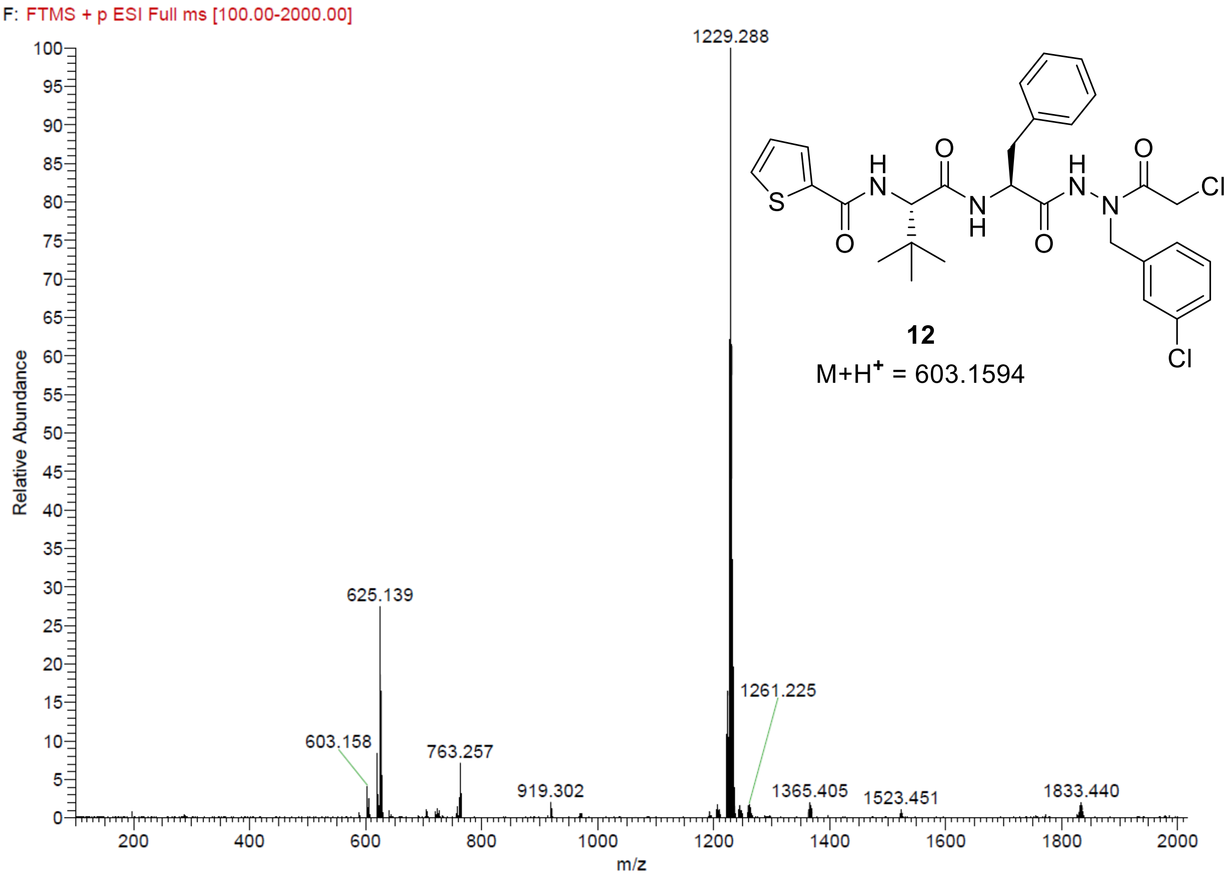
F: FTMS + p ESI Full ms [100.00-2000.00]
Relative Abundance
m/z
1229.288
625.139
603.158
763.257
919.302
1261.225
1365.405
1523.451
1833.440
12
M+H+ = 603.1594

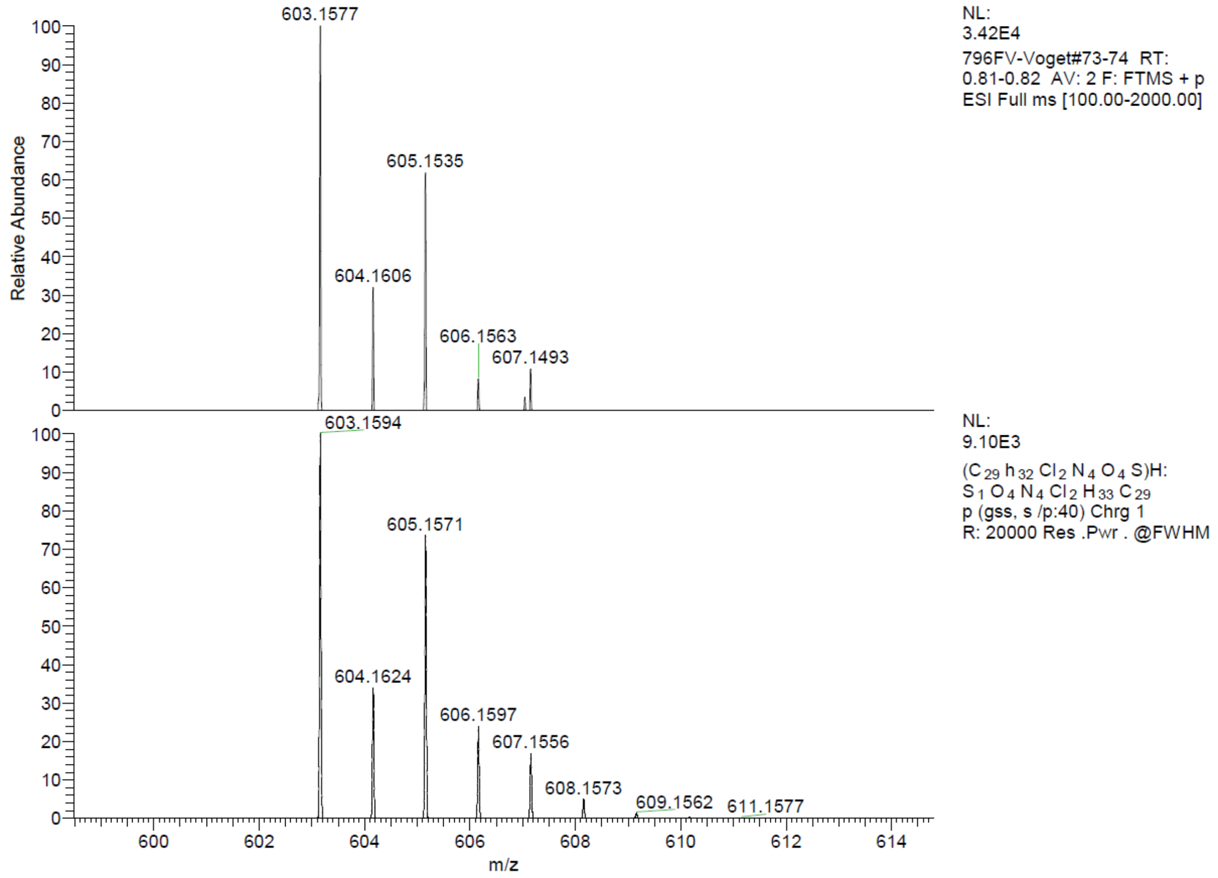
603.1577
605.1535
604.1606
606.1563
607.1493
NL:
3.42E4
796FV-Voget#73-74 RT:
0.81-0.82 AV: 2 F: FTMS + p
ESI Full ms [100.00-2000.00]
603.1594
605.1571
604.1624
606.1597
607.1556
608.1573
609.1562
611.1577
NL:
9.10E3
(C29 h32 Cl2 N4 O4 S)H:
S1 O4 N4 Cl2 H33 C29
p (gss, s /p:40) Chrg 1
R: 20000 Res .Pwr . @FWHM
Relative Abundance
m/z

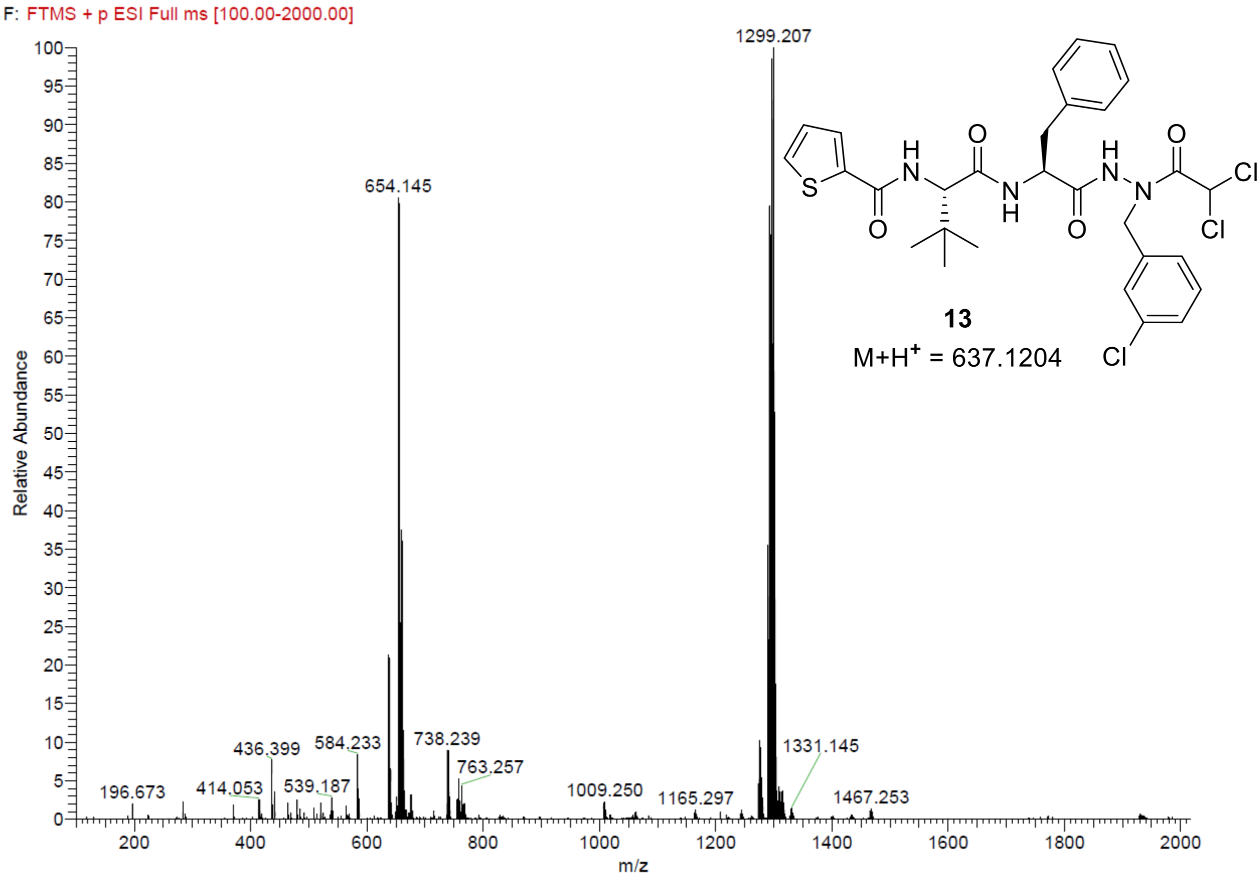
F: FTMS + p ESI Full ms [100.00-2000.00]
Relative Abundance
m/z
1299.207
654.145
436.399
584.233
738.239
763.257
196.673
414.053
539.187
1009.250
1165.297
1331.145
1467.253
13
M+H$^+$ = 637.1204

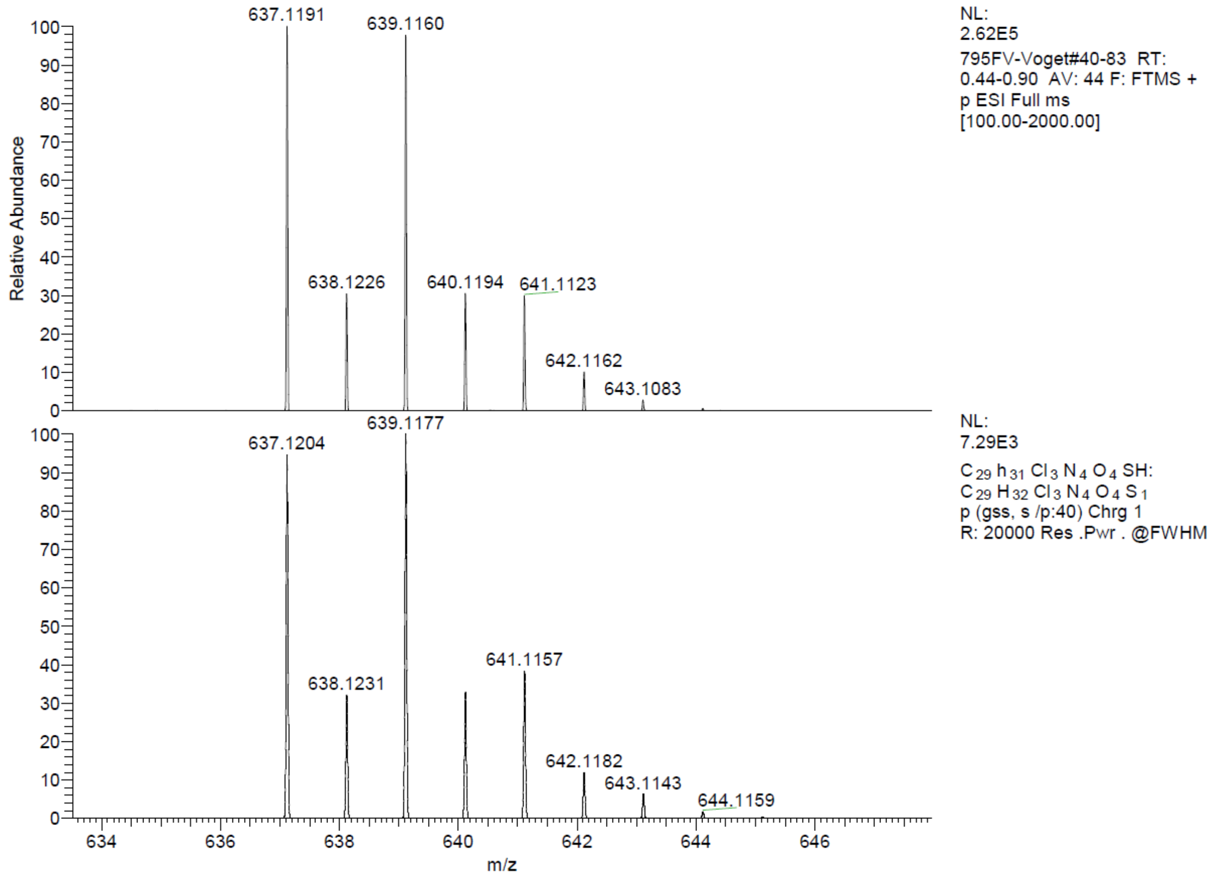
NL:
2.62E5
795FV-Voget#40-83 RT:
0.44-0.90 AV: 44 F: FTMS +
p ESI Full ms
[100.00-2000.00]
637.1191
639.1160
638.1226
640.1194
641.1123
642.1162
643.1083
NL:
7.29E3
C 29 h 31 Cl 3 N 4 O 4 SH:
C 29 H 32 Cl 3 N 4 O 4 S 1
p (gss, s /p:40) Chrg 1
R: 20000 Res .Pwr . @FWHM
637.1204
639.1177
638.1231
641.1157
642.1182
643.1143
644.1159
Relative Abundance
m/z

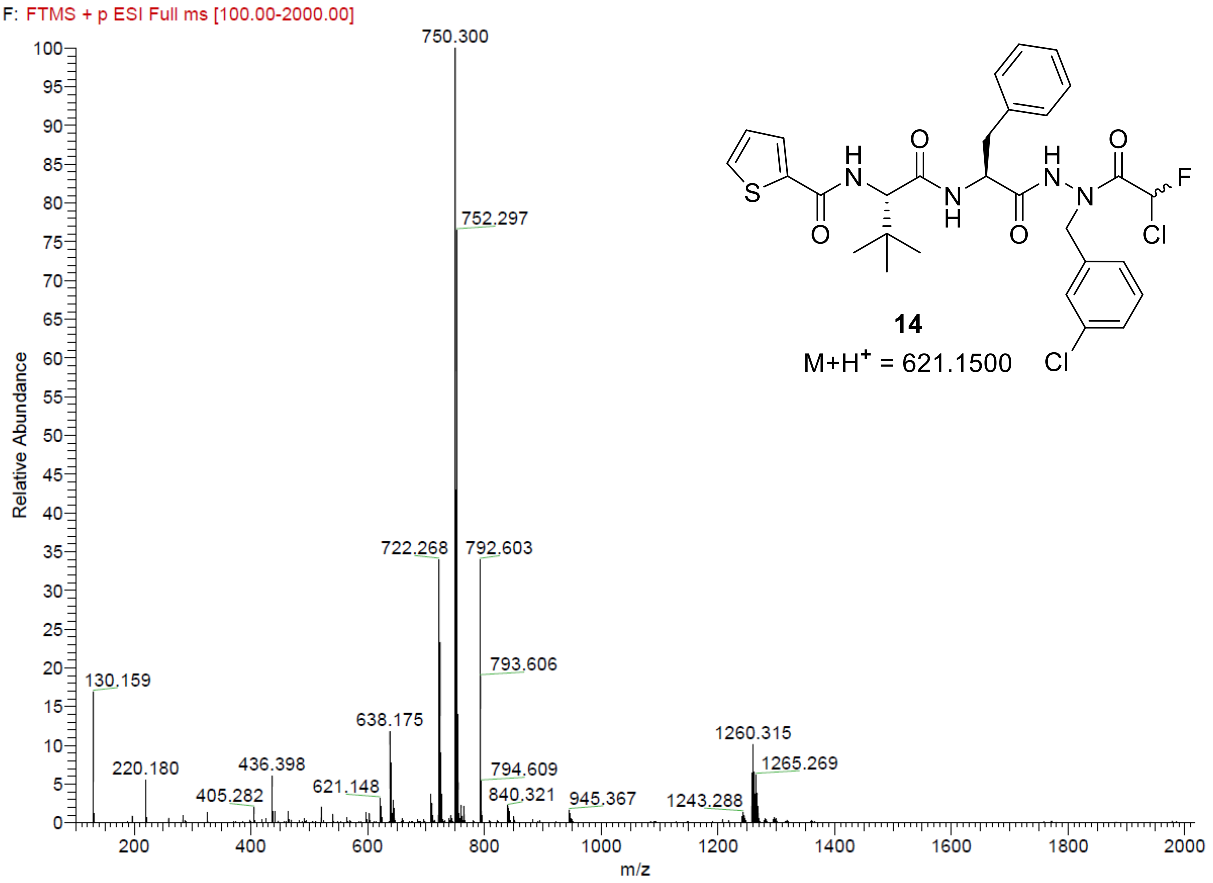
F: FTMS + p ESI Full ms [100.00-2000.00]
750.300
752.297
722.268
792.603
793.606
130.159
638.175
1260.315
220.180
436.398
794.609
1265.269
405.282
621.148
840.321
945.367
1243.288
Relative Abundance
m/z
14
M+H+ = 621.1500

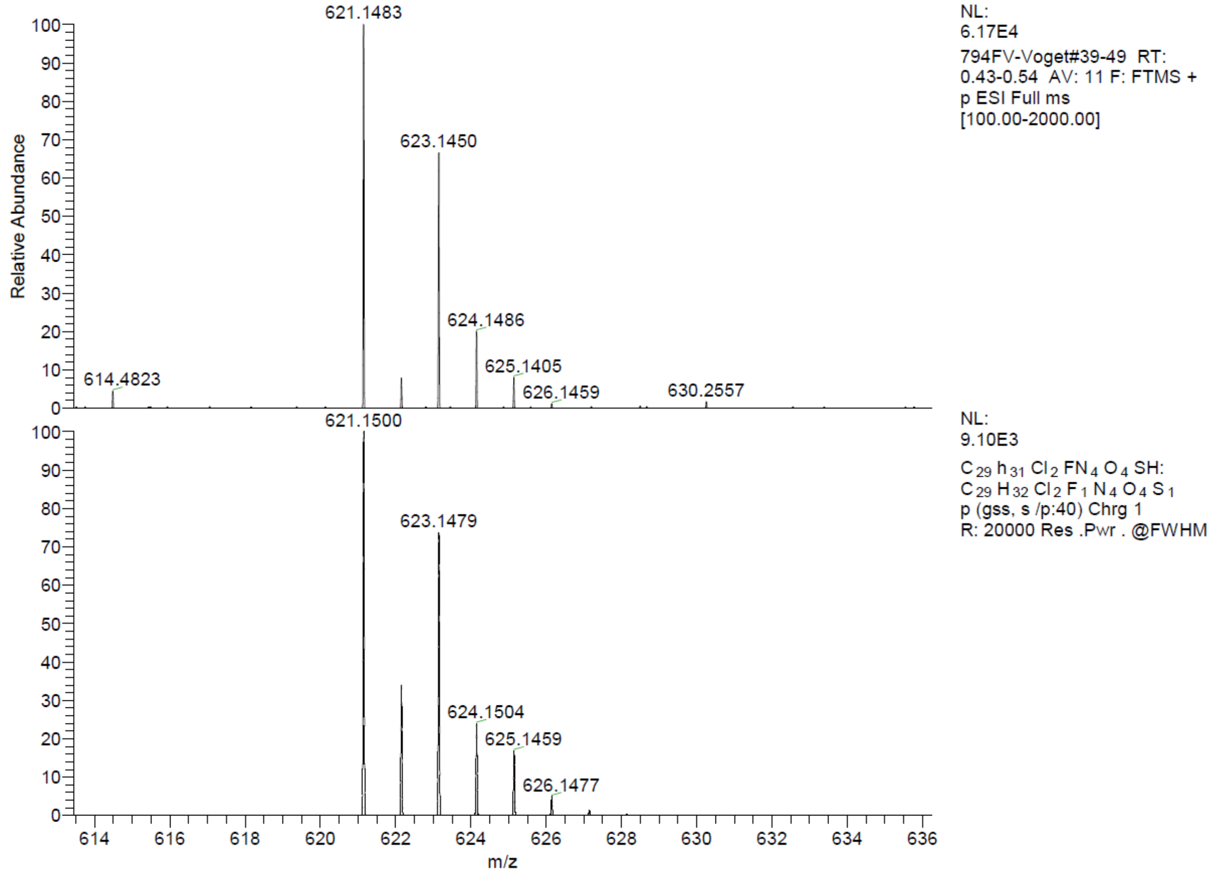
621.1483
623.1450
624.1486
625.1405
614.4823
626.1459
630.2557
NL:
6.17E4
794FV-Voget#39-49 RT: 0.43-0.54 AV: 11 F: FTMS + p ESI Full ms [100.00-2000.00]
621.1500
623.1479
624.1504
625.1459
626.1477
NL:
9.10E3
C 29 h 31 Cl 2 FN 4 O 4 SH: C 29 H 32 Cl 2 F 1 N 4 O 4 S 1
p (gss, s /p:40) Chrg 1
R: 20000 Res .Pwr . @FWHM
Relative Abundance
m/z

4. LC-MS Traces

Compound 1

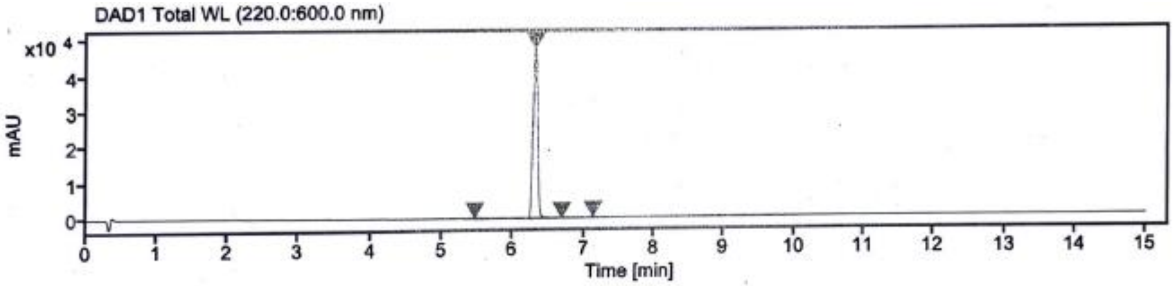

Signal:	DAD1 Total WL (220.0:600.0 nm)				
RT [min]	Peak MS Base Peak m/z	Area	Area%	Max Peak%	Height
5.457		1389.4719	0.6525	0.664	275.893
6.299		209164.5537	98.2170	100.000	48177.721
6.687		796.5496	0.3740	0.381	101.266
7.131		1611.1136	0.7565	0.770	392.709
	Sum	212961.6889			

Compound 2

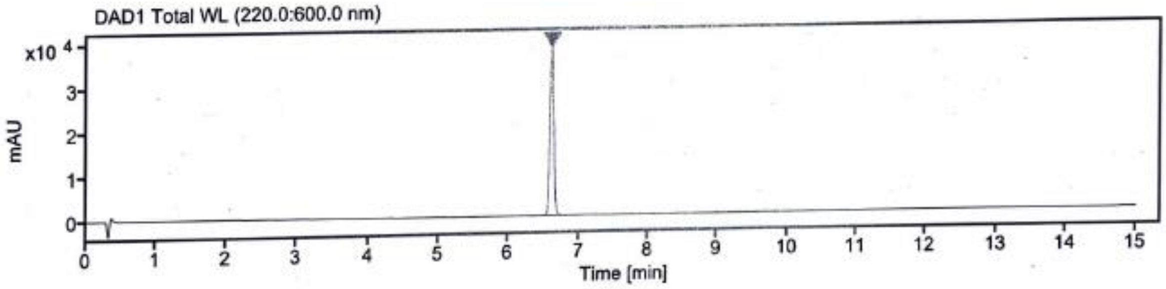

Signal:	DAD1 Total WL (220.0:600.0 nm)				
RT [min]	Peak MS Base Peak m/z	Area	Area%	Max Peak%	Height
6.605		150610.5612	100.0000	100.000	38936.148
	Sum	150610.5612			

Compound 3

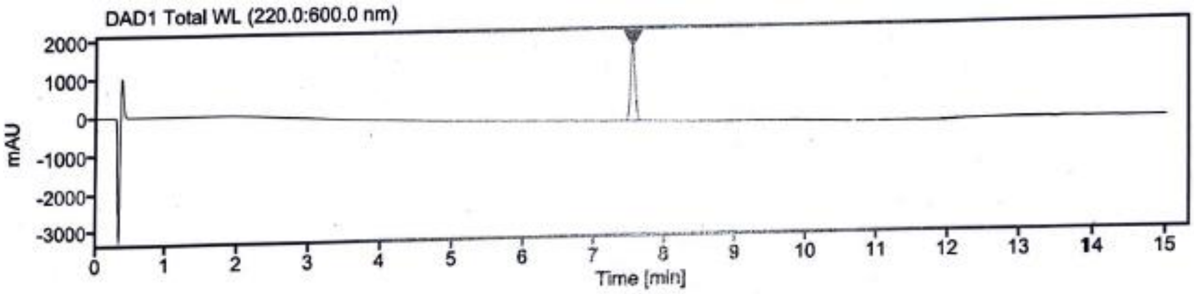

Signal: DAD1 Total WL (220.0:600.0 nm)

RT [min]	Peak MS Base Peak m/z	Area	Area%	Max Peak%	Height
7.518	400.600	9192.6238	100.0000	100.000	1993.916
	Sum	9192.6238			

Compound 4

A:

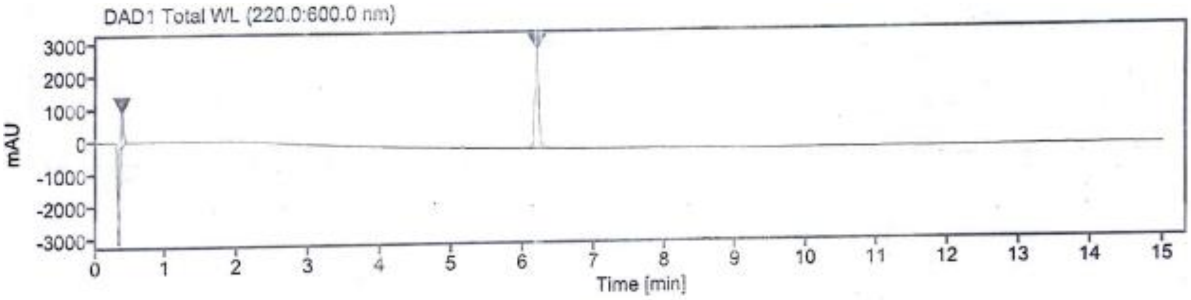

Signal: DAD1 Total WL (220.0:600.0 nm)

RT [min]	Peak MS Base Peak m/z	Area	Area%	Max Peak%	Height
0.378		2329.6098	16.3757	19.582	1049.953
6.171		11896.4017	83.6243	100.000	3083.264
	Sum	14226.0115			

B:

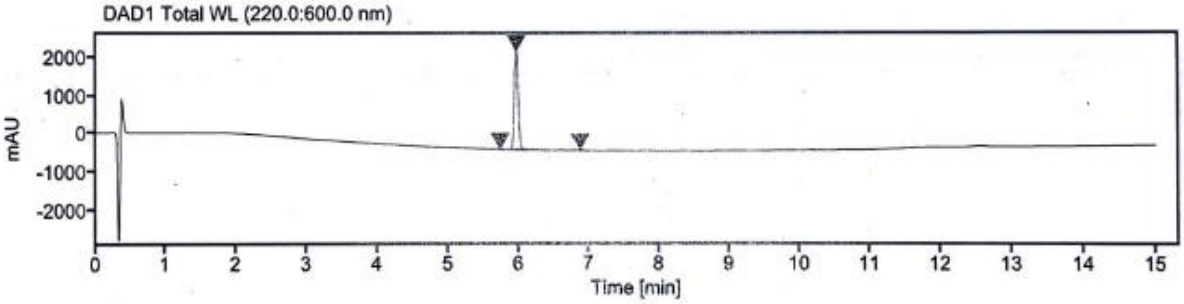

Signal: DAD1 Total WL (220.0:600.0 nm)

RT [min]	Peak MS Base Peak m/z	Area	Area%	Max Peak%	Height
5.717		41.5974	0.4253	0.431	13.035
5.947		9646.7051	98.6193	100.000	2590.622
6.860		93.4625	0.9555	0.969	21.406
	Sum	9781.7650			

Compound 5

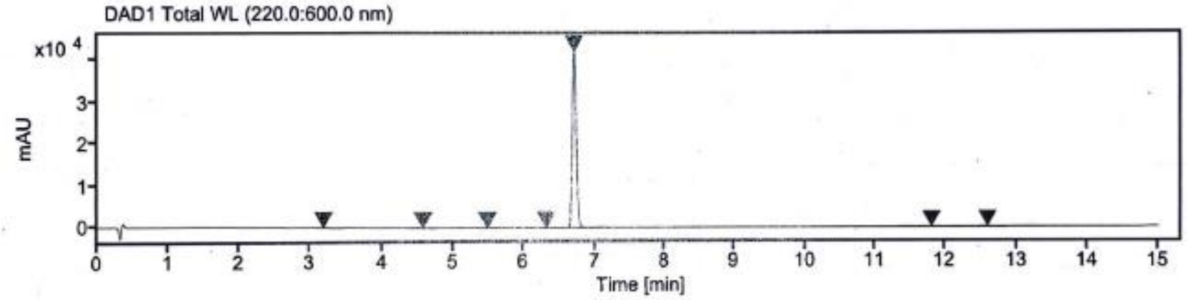

Signal:	DAD1 Total WL (220.0:600.0 nm)				
**RT [min]**	**Peak MS Base Peak m/z**	**Area**	**Area%**	**Max Peak%**	**Height**
3.180		408.0655	0.2374	0.240	38.983
4.572		117.7376	0.0685	0.069	48.958
5.480		228.6268	0.1330	0.135	32.494
6.316		101.9528	0.0593	0.060	22.030
6.711	452.300	169752.3965	98.7645	100.000	42398.804
11.810		511.7299	0.2977	0.301	17.670
12.602		755.5003	0.4396	0.445	56.766
	Sum	171876.0095			

Compound 6

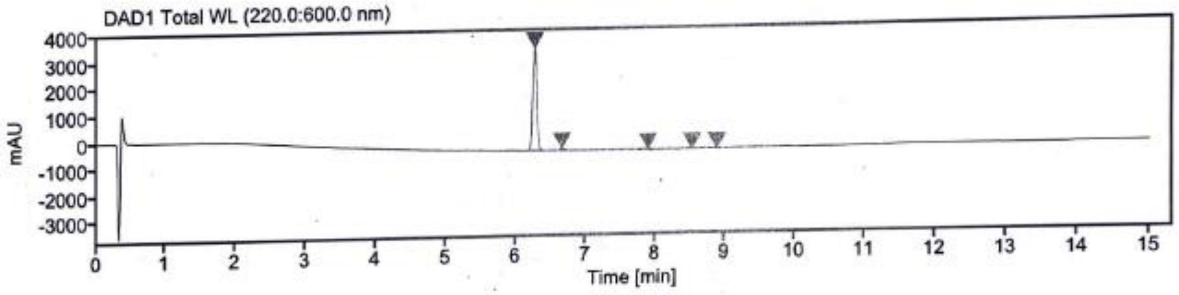

Signal:	DAD1 Total WL (220.0:600.0 nm)				
**RT [min]**	**Peak MS Base Peak m/z**	**Area**	**Area%**	**Max Peak%**	**Height**
6.258	421.400	14700.7607	96.1310	100.000	3875.847
6.648		394.1876	2.5777	2.681	90.312
7.889		72.0447	0.4711	0.490	22.124
8.521		87.0192	0.5690	0.592	6.335
8.878		38.4080	0.2512	0.261	6.767
	Sum	15292.4202			

Compound 7

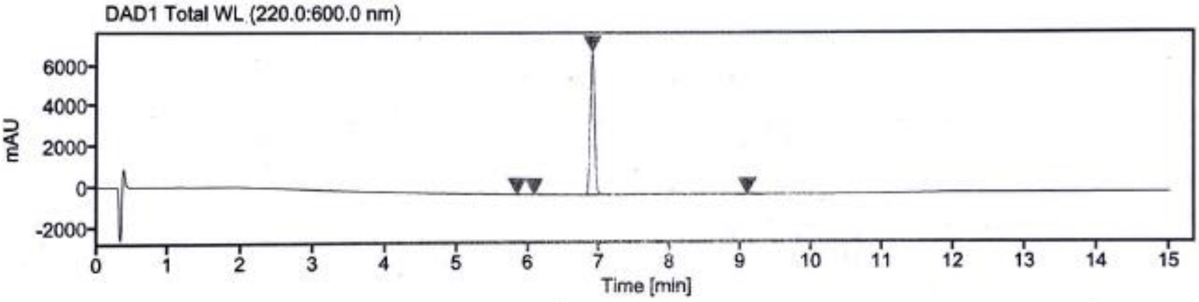

Signal:	DAD1 Total WL (220.0:600.0 nm)				
RT [min]	Peak MS Base Peak m/z	Area	Area%	Max Peak%	Height
5.826		51.7894	0.1732	0.175	13.881
6.063		115.6367	0.3867	0.390	12.638
6.886	455.500	29637.5998	99.1184	100.000	7121.706
9.078		96.1881	0.3217	0.325	12.098
	Sum	29901.2140			

Compound 8

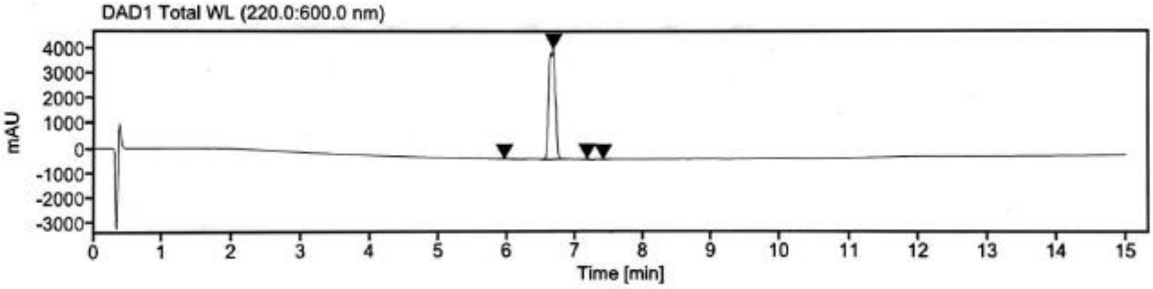

Signal:	DAD1 Total WL (220.0:600.0 nm)				
RT [min]	Peak MS Base Peak m/z	Area	Area%	Max Peak%	Height
5.942		82.0198	0.2753	0.278	8.460
6.660	439.300	29499.0506	99.0302	100.000	4426.085
7.157		163.5020	0.5489	0.554	44.928
7.397		43.3739	0.1456	0.147	10.362
	Sum	29787.9462			

Compound 9

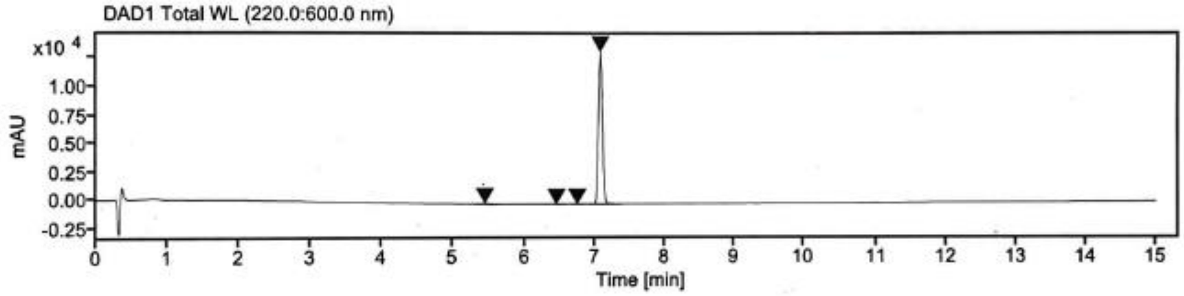

Signal: DAD1 Total WL (220.0:600.0 nm)

RT [min]	Peak MS Base Peak m/z	Area	Area%	Max Peak%	Height
5.444		369.8016	0.6762	0.682	80.995
6.459		14.1099	0.0258	0.026	5.373
6.754		64.8784	0.1186	0.120	18.653
7.083		54237.6387	99.1793	100.000	13514.334
	Sum	54686.4286			

Compound 10

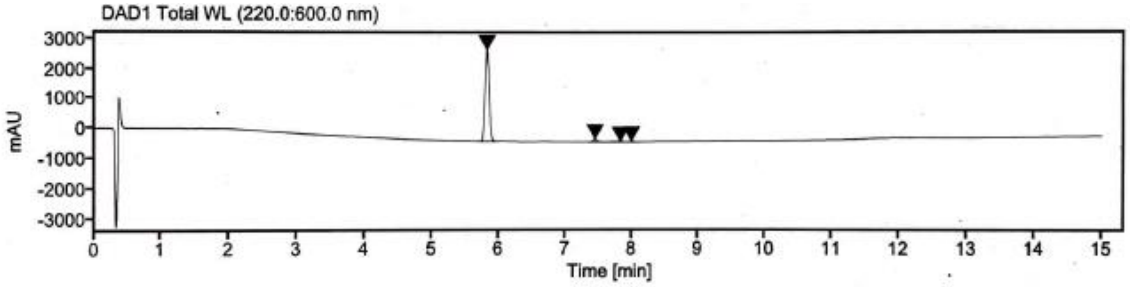

Signal: DAD1 Total WL (220.0:600.0 nm)

RT [min]	Peak MS Base Peak m/z	Area	Area%	Max Peak%	Height
5.807	367.200	13072.9326	96.2258	100.000	3046.893
7.433		370.3051	2.7257	2.833	91.560
7.812		82.7558	0.6091	0.633	9.746
7.981		59.6883	0.4393	0.457	15.960
	Sum	13585.6817			

Compound 12

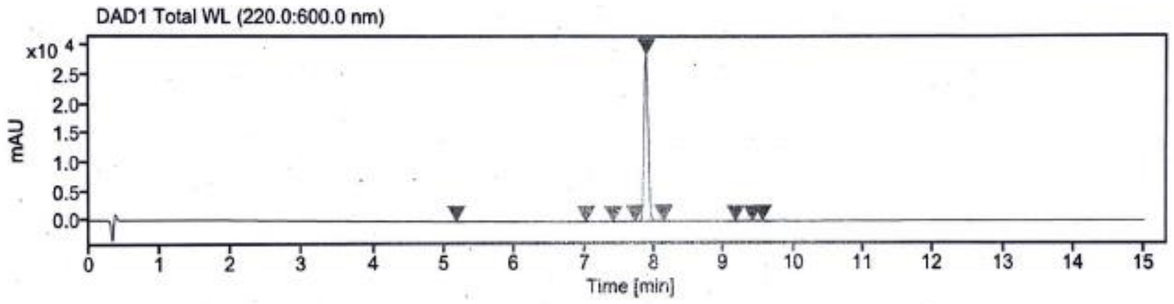

Signal: DAD1 Total WL (220.0:600.0 nm)

RT [min]	Peak MS Base Peak m/z	Area	Area%	Max Peak%	Height
5.165		114.7914	0.0979	0.100	31.727
7.018		273.6409	0.2334	0.238	27.916
7.406		82.7248	0.0706	0.072	15.426
7.721		328.7368	0.2804	0.285	89.771
7.879		115168.1127	98.2236	100.000	29081.826
8.130		562.2922	0.4796	0.488	126.834
9.164		142.9024	0.1219	0.124	34.184
9.408		289.4195	0.2468	0.251	34.921
9.558		288.3197	0.2459	0.250	57.774
	Sum	117250.9403			

Compound 13

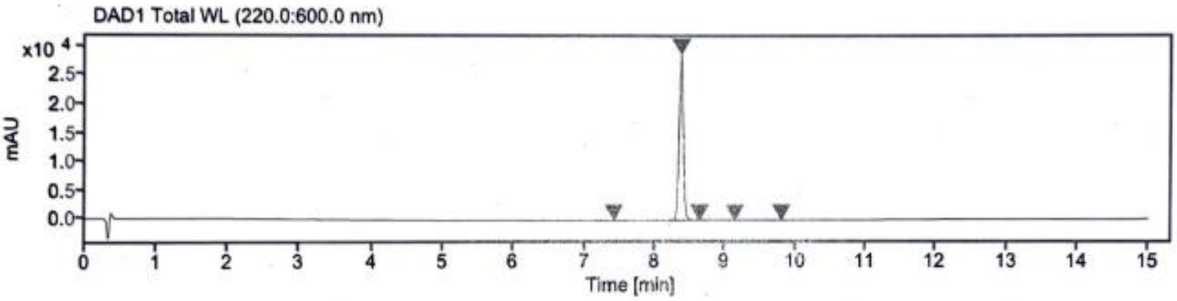

Signal: DAD1 Total WL (220.0:600.0 nm)

RT [min]	Peak MS Base Peak m/z	Area	Area%	Max Peak%	Height
7.415		376.1954	0.3145	0.317	83.825
8.365	639.100	118640.6964	99.1684	100.000	29127.882
8.632		187.3573	0.1566	0.158	50.514
9.141		173.4293	0.1450	0.146	31.930
9.802		257.9639	0.2156	0.217	58.756
	Sum	119635.6423			

Compound 14

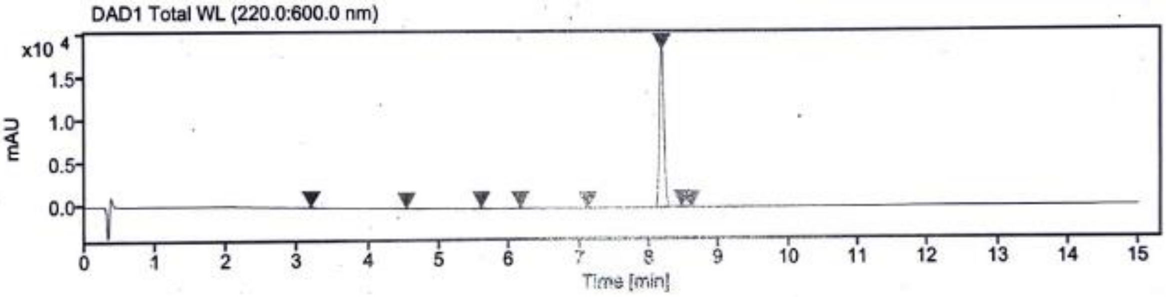

Signal: DAD1 Total WL (220.0:600.0 nm)

RT [min]	Peak MS Base Peak m/z	Area	Area%	Max Peak%	Height
3.187		786.0121	0.9185	0.955	150.976
4.529		295.7992	0.3456	0.359	72.222
5.591		704.5822	0.8233	0.856	179.218
6.151		570.2233	0.6663	0.693	170.089
7.116		239.2804	0.2796	0.291	65.795
8.177	619.200	82306.6233	96.1754	100.000	18627.704
8.463		511.1260	0.5973	0.621	160.538
8.595		166.0426	0.1940	0.202	68.564
	Sum	85579.6892			

Compound 15

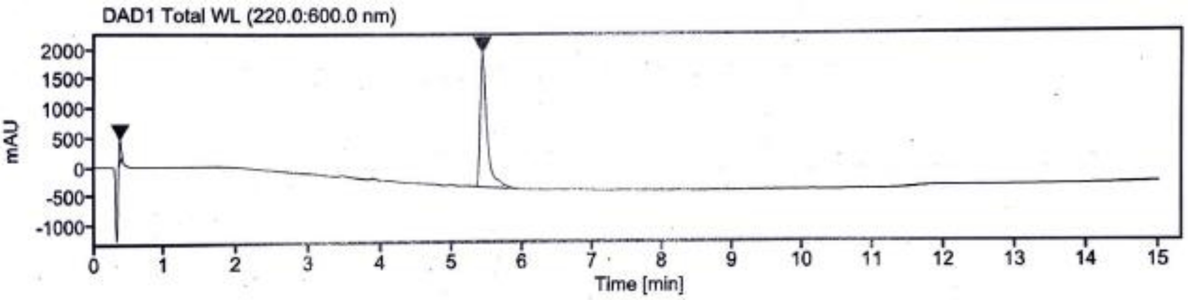

Signal: DAD1 Total WL (220.0:600.0 nm)

RT [min]	Peak MS Base Peak m/z	Area	Area%	Max Peak%	Height
0.374		648.8335	3.9658	4.130	373.861
5.413	217.900	15711.8875	96.0342	100.000	2318.744
	Sum	16360.7210			

Compound 17

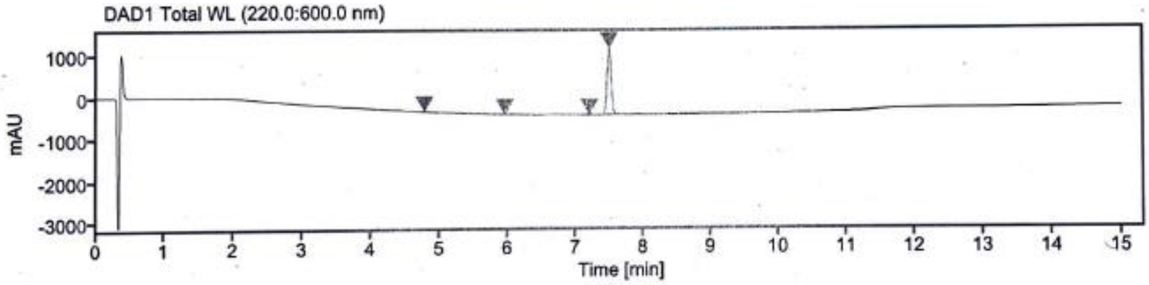

Signal: DAD1 Total WL (220.0:600.0 nm)

RT [min]	Peak MS Base Peak m/z	Area	Area%	Max Peak%	Height
4.767		109.1612	1.5989	1.684	15.301
5.945		153.0624	2.2420	2.362	8.423
7.188		84.1069	1.2320	1.298	19.950
7.479	317.200	6480.7891	94.9271	100.000	1616.280
	Sum	6827.1195			

Compound 18

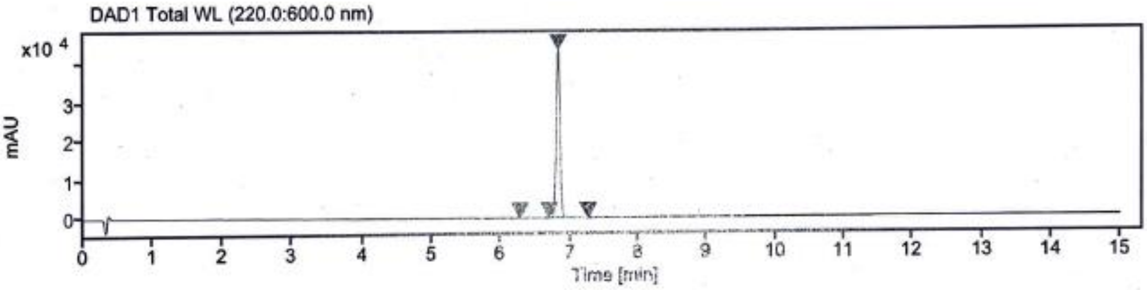

Signal:	DAD1 Total WL (220.0:600.0 nm)				
RT [min]	Peak MS Base Peak m/z	Area	Area%	Max Peak%	Height
6.271		262.8040	0.1550	0.156	67.782
6.692		300.4211	0.1772	0.178	105.421
6.822		168607.9010	99.4476	100.000	44262.609
7.262		373.3705	0.2202	0.221	100.374
	Sum	169544.4966			

Compound 19

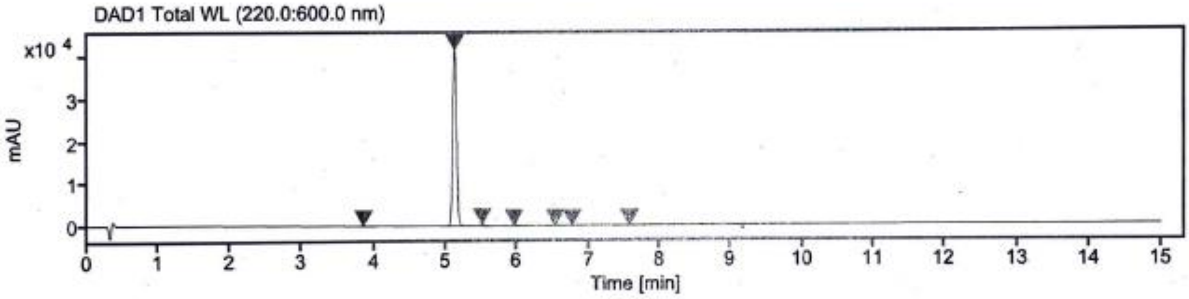

Signal:	DAD1 Total WL (220.0:600.0 nm)				
RT [min]	Peak MS Base Peak m/z	Area	Area%	Max Peak%	Height
3.853		370.1782	0.2421	0.248	89.098
5.118		149258.7314	97.6244	100.000	42246.062
5.502		1668.1439	1.0911	1.118	458.743
5.954		861.8057	0.5637	0.577	158.058
6.525		255.9275	0.1674	0.171	49.350
6.764		196.3462	0.1284	0.132	31.111
7.568		279.7087	0.1829	0.187	41.757
	Sum	152890.8416			

Compound 20

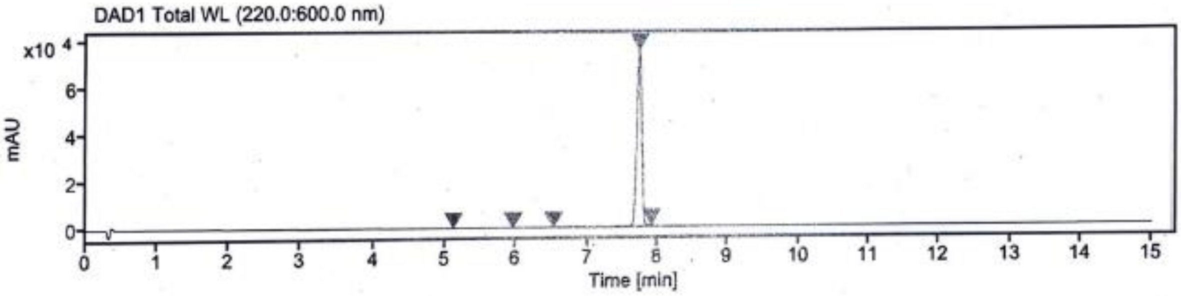

Signal:	DAD1 Total WL (220.0:600.0 nm)				
RT [min]	Peak MS Base Peak m/z	Area	Area%	Max Peak%	Height
5.118		441.1604	0.1178	0.120	131.444
5.954		341.4993	0.0912	0.093	66.152
6.528		556.2518	0.1486	0.151	81.842
7.739	523.500	367908.8736	98.2589	100.000	76379.766
7.914		5180.3229	1.3835	1.408	939.452
	Sum	374428.1080			

Compound 21

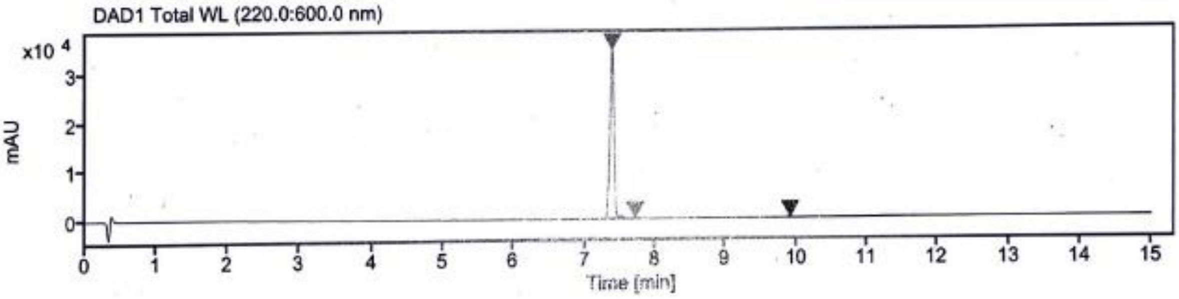

Signal:	DAD1 Total WL (220.0:600.0 nm)				
RT [min]	Peak MS Base Peak m/z	Area	Area%	Max Peak%	Height
7.384	527.600	143565.9671	99.2899	100.000	35227.566
7.714		625.5661	0.4326	0.436	162.412
9.915		401.2386	0.2775	0.279	89.170
	Sum	144592.7718			
