# Supplementary material for: Sequential Optimization Approach Toward an Azapeptide‐Based SARS‐CoV‐2 Main Protease Inhibitor
Source: Arch Pharm (Weinheim). 2025 Dec 23;358(12):e70175. doi: 10.1002/ardp.70175 (PMC12723578; doi:10.1002/ardp.70175)
Supplement: Supplementary file 2 — ArchPharm_SupplMat_InChI_2020. [file ARDP-358-e70175-s001.doc]

**Supplemental Material: Novel Compounds and Biological Screening Results**

Sequential Optimization Approach Towards an Azapeptide-Based SARS-CoV-2 Main Protease Inhibitor

Rabea Voget,^1^ Victoria Steiger,^1^ Julian Breidenbach,^1^ Katharina Sylvester,^1^ Christin Müller-Ruttloff,^2^ Chun-Chiao Yang,^3^ John Ziebuhr,^2^ Norbert Sträter,^3^ Christa E. Müller,^1^ Michael Gütschow^1,*^

1 Pharmaceutical Institute, Pharmaceutical & Medicinal Chemistry, University of Bonn, 53121 Bonn, Germany

2 Institute of Medical Virology, Justus Liebig University Giessen, 35392 Giessen, Germany

3 Institute of Bioanalytical Chemistry, Center for Biotechnology and Biomedicine, Leipzig University, 04103 Leipzig, Germany

Prof. Dr. Michael Gütschow

Pharmaceutical Institute, Pharmaceutical & Medicinal Chemistry, University of Bonn, 53121 Bonn, Germany

guetschow@uni-bonn.de

| **Compound No.** | **InChI** | **Biological Activity** |
| --- | --- | --- |
| 1 | InChI=1S/C22H25N3O5/c1-25(14-13-20(26)29-2)24-21(27)19(15-17-9-5-3-6-10-17)23-22(28)30-16-18-11-7-4-8-12-18/h3-14,19H,15-16H2,1-2H3,(H,23,28)(H,24,27)/b14-13+/t19-/m0/s1 | 0% inhibition @ 50 µM^a^ |
| 2 | InChI=1S/C24H27N3O7/c1-27(20(23(30)33-3)15-21(28)32-2)26-22(29)19(14-17-10-6-4-7-11-17)25-24(31)34-16-18-12-8-5-9-13-18/h4-13,15,19H,14,16H2,1-3H3,(H,25,31)(H,26,29)/b20-15+/t19-/m0/s1 | 2% inhibition @ 50 µM^a^ |
| 3 | InChI=1S/C21H25N3O5/c1-3-28-21(27)24(2)23-19(25)18(14-16-10-6-4-7-11-16)22-20(26)29-15-17-12-8-5-9-13-17/h4-13,18H,3,14-15H2,1-2H3,(H,22,26)(H,23,25)/t18-/m0/s1 | 3% inhibition @ 50 µM^a^ |
| 4 | InChI=1S/C22H25N3O6/c1-25(19(26)14-20(27)30-2)24-21(28)18(13-16-9-5-3-6-10-16)23-22(29)31-15-17-11-7-4-8-12-17/h3-12,18H,13-15H2,1-2H3,(H,23,29)(H,24,28)/t18-/m0/s1 | 0% inhibition @ 50 µM^a^ |
| 5 | InChI=1S/C24H27N3O6/c1-3-32-22(29)15-14-21(28)27(2)26-23(30)20(16-18-10-6-4-7-11-18)25-24(31)33-17-19-12-8-5-9-13-19/h4-15,20H,3,16-17H2,1-2H3,(H,25,31)(H,26,30)/b15-14+/t20-/m0/s1 | 13% inhibition @ 50 µM^a^ |
| 6 | InChI=1S/C20H22ClN3O4/c1-24(18(25)13-21)23-19(26)17(12-15-8-4-2-5-9-15)22-20(27)28-14-16-10-6-3-7-11-16/h2-11,17H,12-14H2,1H3,(H,22,27)(H,23,26)/t17-/m0/s1 | 55% inhibition @ 50 µM^a^  *K*_i_ = 14.8 µM^b^  *k*_inac_/*K*_i_ = 133 M^-1^s^-1 c^ |
| 7 | InChI=1S/C20H21Cl2N3O4/c1-25(19(27)17(21)22)24-18(26)16(12-14-8-4-2-5-9-14)23-20(28)29-13-15-10-6-3-7-11-15/h2-11,16-17H,12-13H2,1H3,(H,23,28)(H,24,26)/t16-/m0/s1 | 100% inhibition @ 50 µM^a^  *K*_i_ = 1.22 µM^b^ |
| 8 | InChI=1S/C20H21ClFN3O4/c1-25(19(27)17(21)22)24-18(26)16(12-14-8-4-2-5-9-14)23-20(28)29-13-15-10-6-3-7-11-15/h2-11,16-17H,12-13H2,1H3,(H,23,28)(H,24,26)/t16-,17?/m0/s1 | 16% inhibition @ 50 µM^a^ |
| 9 | InChI=1S/C20H20ClF2N3O4/c1-26(18(28)20(21,22)23)25-17(27)16(12-14-8-4-2-5-9-14)24-19(29)30-13-15-10-6-3-7-11-15/h2-11,16H,12-13H2,1H3,(H,24,29)(H,25,27)/t16-/m0/s1 | 15% inhibition @ 50 µM^a^ |
| 10 | InChI=1S/C20H22N4O3/c1-24(13-12-21)23-19(25)18(14-16-8-4-2-5-9-16)22-20(26)27-15-17-10-6-3-7-11-17/h2-11,18H,13-15H2,1H3,(H,22,26)(H,23,25)/t18-/m0/s1 | 11% inhibition @ 50 µM^a^ |
| 11 | InChI=1S/C18H20FN3O5S/c1-22(28(19,25)26)21-17(23)16(12-14-8-4-2-5-9-14)20-18(24)27-13-15-10-6-3-7-11-15/h2-11,16H,12-13H2,1H3,(H,20,24)(H,21,23)/t16-/m0/s1 | 11% inhibition @ 50 µM^a^  *K*_i_ = 7.24 µM^b^  *k*_inac_/*K*_i_ = 54.6 M^-1^s^-1 c^ |
| 12 | InChI=1S/C29H32Cl2N4O4S/c1-29(2,3)25(33-27(38)23-13-8-14-40-23)28(39)32-22(16-19-9-5-4-6-10-19)26(37)34-35(24(36)17-30)18-20-11-7-12-21(31)15-20/h4-15,22,25H,16-18H2,1-3H3,(H,32,39)(H,33,38)(H,34,37)/t22-,25+/m0/s1 | 100% inhibition @ 50 µM^a^  *K*_i_ = 0.0230 µM^b^  *k*_inac_/*K*_i_ = 78,900 M^-1^s^-1 c^  EC_50_ = 0.47 µM^d^ |
| 13 | InChI=1S/C29H31Cl3N4O4S/c1-29(2,3)23(34-26(38)22-13-8-14-41-22)27(39)33-21(16-18-9-5-4-6-10-18)25(37)35-36(28(40)24(31)32)17-19-11-7-12-20(30)15-19/h4-15,21,23-24H,16-17H2,1-3H3,(H,33,39)(H,34,38)(H,35,37)/t21-,23+/m0/s1 | 42% inhibition @ 50 µM^a^ |
| 14 | InChI=1S/C29H31Cl2FN4O4S.2CH4/c1-29(2,3)23(34-26(38)22-13-8-14-41-22)27(39)33-21(16-18-9-5-4-6-10-18)25(37)35-36(28(40)24(31)32)17-19-11-7-12-20(30)15-19;;/h4-15,21,23-24H,16-17H2,1-3H3,(H,33,39)(H,34,38)(H,35,37);2*1H4/t21-,23+,24?;;/m0../s1 | 69% inhibition @ 50 µM^a^  *K*_i_ = 4.34 µM^b^  *k*_inac_/*K*_i_ = 1,580 M^-1^s^-1 c^ |

^a^ Screening of M^pro^ inhibition at 50 µM with the fluorogenic substrate Boc-Abu-Tle-Leu-Gln-AMC. Percentage Inhibition of the M^pro^ activity is noted.

^b^ Determination of *K*_i_ values with five different inhibitor concentrations. Ki values were calculation with the Cheng-Prusoff equation.

^c^ Product formation with five different inhibitor concentrations was monitored for 60 min. Non-linear regression of the progress curves gave *k*_obs_ values. These were plotted *versus* inhibitor concentrations. The equation *k*_obs_ = (*k*_inac_ × [I]) / ([I] + *K*_i_ × (1 + [S]/*K*_m_)) was used to obtain *k*_inac_/*K*_i_.

^d^ A549 cells overexpressing human angiotensin-converting enzyme 2 were treated with the inhibitor at seven concentrations for 24 h. Plaque-forming units were quantified and normalized to the control. Non-linear regression yielded the EC_50_ value.
